# Changes in genome organization of parasite-specific gene families during the *Plasmodium* transmission stages

**DOI:** 10.1038/s41467-018-04295-5

**Published:** 2018-05-15

**Authors:** Evelien M. Bunnik, Kate B. Cook, Nelle Varoquaux, Gayani Batugedara, Jacques Prudhomme, Anthony Cort, Lirong Shi, Chiara Andolina, Leila S. Ross, Declan Brady, David A. Fidock, Francois Nosten, Rita Tewari, Photini Sinnis, Ferhat Ay, Jean-Philippe Vert, William Stafford Noble, Karine G. Le Roch

**Affiliations:** 1 Department of Microbiology, Immunology & Molecular Genetics, The University of Texas Health Science Center at San Antonio, 7703 Floyd Curl Drive, San Antonio, TX 78229 USA; 2 Department of Molecular, Cell and Systems Biology, University of California Riverside, 900 University Ave, Riverside, CA 92521 USA; 3 Department of Genome Sciences, University of Washington, 3720 15th Ave NE, Seattle, WA 98195 USA; 4 Department of Statistics, University of California, 367 Evans Hall, Berkeley, CA 94720 USA; 5 Berkeley Institute for Data Science, 190 Doe Library, Berkeley, CA 94720 USA; 6 MINES ParisTech, PSL Research University, CBIO-Centre for Computational Biology, 60 boulevard Saint-Michel, 75006 Paris, France; 7 Institut Curie, 75248 Paris, France; 8 U900, INSERM, Paris, 75248 France; 9 Department of Molecular Microbiology and Immunology, Johns Hopkins Bloomberg School of Public Health, 615N. Wolfe Street, E5132, Baltimore, MD 21205 USA; 10 Centre for Tropical Medicine and Global Health, Nuffield Department of Medicine Research building, University of Oxford, Old Road campus, Roosevelt Drive, Headington, Oxford, OX3 7FZ UK; 11 Shoklo Malaria Research Unit, Mahidol-Oxford Tropical Medicine Research Unit, Faculty of Tropical Medicine, Mahidol University, Mae Sot, Tak, 63110 Thailand; 12 Department of Microbiology and Immunology, Columbia University Medical Center, 701W. 168 St., HHSC 1208, New York, NY 10032 USA; 13 School of Life Sciences, Queens Medical Centre, University of Nottingham, Nottingham, NG7 2UH UK; 14 Division of Infectious Diseases, Department of Medicine, Columbia University, New York, NY 10032 USA; 15 La Jolla Institute for Allergy & Immunology, 9420 Athena Cir, La Jolla, CA 92037 USA; 16 Département de mathématiques et applications, École normale supérieure, CNRS, PSL Research University, Paris, 75005 France; 17 Department of Computer Science and Engineering, University of Washington, Seattle, WA 98195 USA

## Abstract

The development of malaria parasites throughout their various life cycle stages is coordinated by changes in gene expression. We previously showed that the three-dimensional organization of the *Plasmodium falciparum* genome is strongly associated with gene expression during its replication cycle inside red blood cells. Here, we analyze genome organization in the *P. falciparum* and *P. vivax* transmission stages. Major changes occur in the localization and interactions of genes involved in pathogenesis and immune evasion, host cell invasion, sexual differentiation, and master regulation of gene expression. Furthermore, we observe reorganization of subtelomeric heterochromatin around genes involved in host cell remodeling. Depletion of heterochromatin protein 1 (PfHP1) resulted in loss of interactions between virulence genes, confirming that PfHP1 is essential for maintenance of the repressive center. Our results suggest that the three-dimensional genome structure of human malaria parasites is strongly connected with transcriptional activity of specific gene families throughout the life cycle.

## Abstract (web summary)

The development of malaria parasites is controlled by coordinated changes in gene expression. Here, the authors show that the three-dimensional genome structure of human malaria parasites is strongly connected with transcriptional activity of specific gene families throughout the life cycles of *Plasmodium falciparum* and *Plasmodium vivax* parasites.

## Introduction

With an estimated 438,000 deaths per year, malaria is still one of the most deadly infectious diseases, mostly targeting young children in sub-Saharan Africa^1^. The disease is caused by one of five parasites of the *Plasmodium* genus, of which *P. falciparum* is the most common and deadliest. *P. vivax* is also responsible for significant disease, mostly in Southeast Asia^1^.

*Plasmodium* parasites have complex life cycles that involve a human host and a mosquito vector. Infection in humans starts when an infected female *Anopheles* mosquito takes a blood meal and transmits parasites that are present in the form of sporozoites in her salivary glands. These sporozoites are inoculated into the skin, travel to the liver and establish an infection in hepatocytes. Over a period of several days, the parasite replicates and eventually releases thousands of merozoites into the bloodstream. Alternatively, *P. vivax* can survive in the liver for weeks or years in dormant forms called hypnozoites, which can be reactivated and cause malaria relapses. Merozoites that emerge from the liver start a 48-h replication cycle in red blood cells. During this asexual intraerythrocytic development cycle (IDC), the parasite progresses through three main developmental stages: ring, trophozoite, and schizont, to produce 8–24 daughter parasites, which burst from the cell and invade new erythrocytes. During the IDC, the parasite can commit to differentiation into male and female gametocytes, which can be taken up by another mosquito. Inside the mosquito, the parasite undergoes sexual reproduction and further develops through several stages into the salivary gland sporozoites that can be transmitted to a new human host.

Understanding how the transitions between the various life cycle stages of the *Plasmodium* parasite are regulated remains an important goal in malaria research. Stage transitions are regulated by coordinated changes in gene expression, but it is still largely unknown how these changes in transcriptional profiles are controlled at the transcriptional level. Only a single family of ApiAP2 transcription factors (TFs) with 27 members has been identified, while approximately two-thirds of the TFs expected based on the size of the *Plasmodium* genome seem to be missing^2^. Several of these ApiAP2 TFs are involved in stage transitions, such as PfAP2-G, which is thought to be the main driver for gametocyte differentiation^3–5^. Our understanding of how these TFs are controlled and how various TFs may act together to form transcriptional networks is still very limited.

In recent years, considerable insight has been gained into the role of epigenetics, chromatin structure, and genome organization in gene regulation, mostly during the IDC of *P. falciparum*. Studies show that the parasite genome is largely in an active, euchromatic state^6–8^, while members of several parasite-specific gene families involved in virulence (*var*, *rifin*, *stevor*, and *pfmc-2tm*), erythrocyte remodeling (*phist*, *hyp*, *fikk*, and others) and solute transport (*clag3)* are organized into heterochromatin^7,9–11^. In particular, the family of *var* genes has received much attention, since these genes are key to pathogenesis and immune escape. Out of a total of 60 *var* genes, only a single variant is expressed within an individual parasite, while the other 59 genes are tightly repressed by a combination of isolation into a perinuclear compartment, repressive histone marks, and repressive long non-coding RNAs^7,12–15^.

Previously, we assessed genome organization at the ring, trophozoite, and schizont stages of the IDC in *P. falciparum* using Hi-C experiments (chromosome conformation capture coupled with next-generation sequencing)^16^ and compared our findings to an earlier Hi-C study in ring-stage *P. falciparum*^17^. We observed a strong association between genome architecture and gene expression, suggesting that the three-dimensional organization of the genome is very important for gene regulation. Here, we analyze the genome organization in the transmission stages of the *P. falciparum* life cycle (gametocytes and sporozoites), as well as for *P. vivax* sporozoites, and present a comparative analysis of genome organization throughout the different life cycle stages. Finally, to meaningfully compare changes in genome organization throughout the *Plasmodium* life cycle, we developed a novel statistical test to detect loci that differ significantly in their intrachromosomal contact count numbers between stages. While large-scale features of chromosome organization are preserved in the sexually differentiated stages, we observe several stage-specific specific changes, including reorganization of genes encoding rDNA, invasion proteins, and transcription factors. During gametocytogenesis, heterochromatic regions at the ends of chromosomes expand to include genes involved in host cell remodeling and a broad superdomain is created on chromosome 14. A prominent feature of genome organization in the sporozoite stage is the establishment of long-range DNA interactions for genes involved in sporozoite migration and hepatocyte invasion. Our results provide important novel insights into the connection between genome organization, heterochromatin, and stage-specific gene expression.

## Results

### Capturing genome conformation of *Plasmodium* transmission stages

**Fig. 1:**
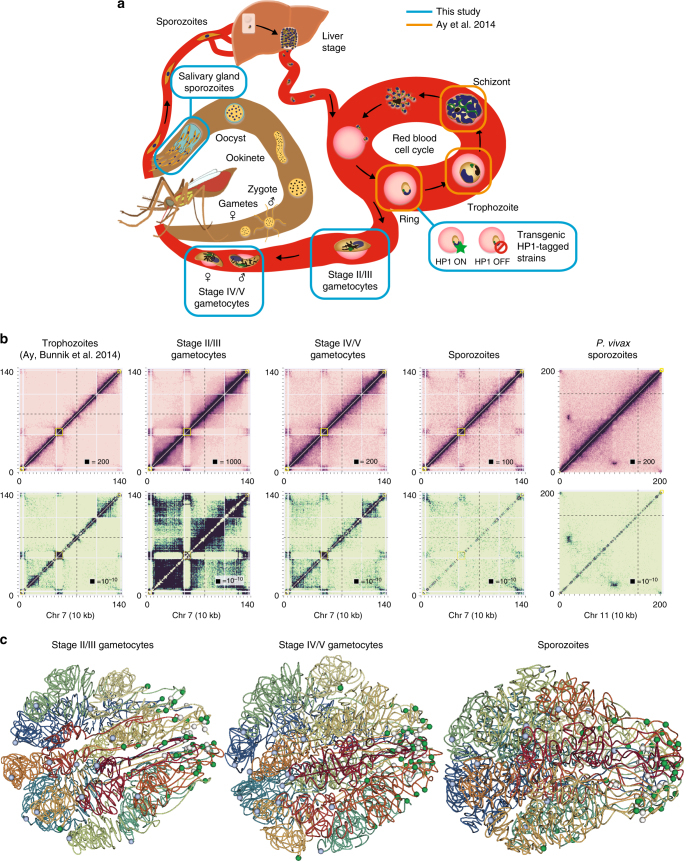
Genome organization in *Plasmodium* parasites. **a** Schematic overview of the parasite life cycle, with the samples generated in this study highlighted in blue and stages available from a previous study^16^ shown in orange. **b** ICE-normalized contact count matrices (top row) and fit-hi-c *p*-value matrices (bottom row) at 10 kb resolution of chromosome 7 for *P. falciparum* stages and chromosome 11 for *P. vivax* sporozoites. The boxed value indicates the maximum contact count (top row) or minimum *p*-value (bottom row). In all other figures comparing different stages, the contact counts were subsampled to the same total. Virulence clusters are indicated by yellow boxes, the centromere location by a dashed black line, and unmappable regions by gray in these and all other heatmaps. **c** Models of the consensus three-dimensional organization of the *P. falciparum* genome in stage II/III gametocytes, stage IV/V gametocytes, and salivary gland sporozoites, with light blue spheres indicating centromeres, white spheres indicating telomeres and green spheres indicating the location of virulence gene clusters

To complement our previous study describing the genome architecture of *P. falciparum* during the intraerythrocytic developmental cycle (IDC)^16^, we performed Hi-C experiments on three additional stages of the *P. falciparum* life cycle: early gametocytes (stage II/III), late gametocytes (stage IV/V), and salivary gland sporozoites (Fig. 1a, Supplementary Table 1, and Supplementary Fig. 1) using the tethered conformation capture methodology. In addition, to evaluate similarities and differences in genome organization between the highly pathogenic *P. falciparum* and the less virulent *P. vivax*, we generated Hi-C data for *P. vivax* salivary gland sporozoites. For each stage, we obtained high-quality data, evidenced by a log-linear relationship between contact probability and genomic distance (Supplementary Fig. 2A), as well as interchromosomal contact probability (ICP) and percentage of long-range contacts (PLRC) values in agreement with previous studies (Supplementary Table 2). Biological replicates of both *P. falciparum* and *P. vivax* sporozoite stage parasites showed a high degree of similarity (Supplementary Fig. 2B, C), demonstrating the robustness of our methodology. The data from these replicates were combined to obtain higher resolution for subsequent analyses.

Next, the observed intrachromosomal and interchromosomal contacts were aggregated into contact count matrices at 10-kb resolution and were normalized using the ICE method to correct for experimental and technical biases^18^ (Fig. 1b, Supplementary Data 1, and Supplementary Data 2). In addition, we identified significant contacts using fit-hi-c, which controls for the propensity of adjacent loci to have more contacts and calculates a *p*-value reflecting the probability that the number of contacts in that bin is larger than expected by chance^19^. We inferred a consensus 3D genome structure for each of the transmission stages and the three IDC stages using Pastis^20^ (Fig. 1c). The stability of these consensus structures was assessed by generating 5000 possible structures from varying initial starting points. A principal component analysis showed strong clustering of structures from the same stage, and clear separation between structures of different stages, except for early and late gametocytes (Supplementary Fig. 2D). These results indicate that the genome organization of early and late gametocytes is similar, while there are distinct differences between all other stages that are captured using a single representative structure for each stage.

### Universal and stage-specific features of genome organization

From the contact count matrices and the consensus 3D structures, it became apparent that large-scale features of genome organization at the gametocyte stage were comparable to those of the IDC stages, including colocalization of centromeres and clustering of telomeres and virulence genes (all *p*-values < 0.00001, Witten–Noble colocalization test^21^). However, we observed significant intrachromosomal rearrangements, including increased interactions among virulence genes and exported proteins, repression of invasion genes, change in organization of ribosomal DNA genes, as well as the formation of large domains on chromosome 14 in close proximity to a female gametocyte-specific *pfap2* transcription factor locus. These changes will be addressed in more detail in the next sections. At the sporozoite stage of *P. falciparum*, the clustering of telomeres was conserved, but the colocalization of the centromeres was completely lost (*p*-value = 0.49, Witten–Noble colocalization test), Fig. 1c, rightmost panel, and Supplementary Fig. 3A). In contrast, in *P. vivax* sporozoites, the centromeres colocalized significantly (*p*-value < 0.0001, Witten-–Noble colocalization test), but these interactions were only observed at the centromere itself and did not involve any of the surrounding regions (Supplementary Fig. 3B). While our result will need to be validated by an independent approach, our Hi-C experiment is so far the only successful technique that has been able to monitor a reduction in centromere clustering in the sporozoite stage. To better visualize the large-scale differences between the various stages of the *P. falciparum* life cycle, we generated an animation of the changes in genome organization during the stage transitions, which highlights that chromosomes undergo dramatic rearrangement in sporozoites as compared to the blood stages (Supplementary Movie 1).

The groups of genes described in the previous sections were examined independently and were selected based on our prior knowledge of the function of these genes, rather than as the result of a systematic screen for changes in contact counts. To systematically identify changes in genome conformation between the various stages, we designed a statistical test that analyzes differences in the number of intrachromosomal contacts for each 10-kb bin in the normalized contact count matrices between pairs of stages. Loci that show a two-fold or larger difference in contacts, after normalizing for the effect of genomic distance, with a false discovery rate of less than 1% are listed in Supplementary Data 3. As expected, interactions at the sporozoite stages were most different from those in other stages of the life cycle. Several chromosomes showed large rearrangements towards the chromosome ends, for example the right arm of chromosome 4 (Supplementary Fig. 4), involving gene families coding for exported proteins involved in virulence and erythrocyte remodeling. The fold-change heatmaps (accessible at http://noble.gs.washington.edu/proj/plasmo3d_sexualstages/), the contact count heatmaps, and the confidence score heatmaps showed many additional differences in chromosome conformation during stage transitions, as detailed below. The interaction patterns were similar in two distinct field isolates for sporozoites and in two laboratory strains for gametocytes, suggesting that genetic variations did not influence our results. Furthermore, the Hi-C methodology has recently been used to detect translocations in genomes and to correct genome assemblies based on Illumina and/or PacBio sequencing in many organisms, including *Plasmodium knowlesi*, *Arabidopsis thaliana*, and *Aedes aegypti*^22–25^. It is therefore unlikely that the changes that we observed between life cycle stages are artifacts caused by genomic recombination during in vitro parasite culture, since none of the interchromosomal heatmaps (Supplementary Fig. 3A) showed any evidence of such recombination events. To further validate our results, we have introduced interchromosomal and intrachromosomal translocations in the *P. vivax* genome to visualize the aberrant patterns that such recombination events would produce (Supplementary Fig. 5). In addition, we scanned our samples using a recently published metric developed to detect genome assembly errors in Hi-C data^24^ and did not detect any signs of misassembly or translocations (Supplementary Fig. 6). Additional simulations showed that translocations of a single 10-kb bin are not easily detectable, but larger regions are detectable when there is a separation of a few bins between them (Supplementary Fig. 7).

### *pfap2-g* leaves the repressive center during gametocytogenesis

**Fig. 2:**
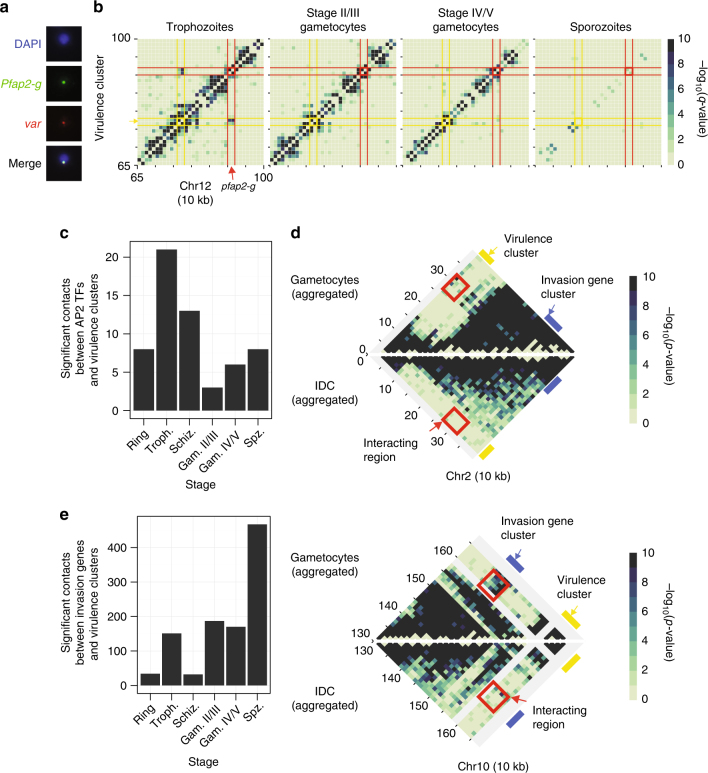
Changes in interaction of *pfap2* genes and invasion genes with the repressive center. **a** Colocalization of *pfap2-g* and *var* gene PF3D7_0800300 by DNA-FISH. Additional images are presented in Supplementary Fig. 8. **b** Dissociation of the gametocyte-specific transcription factor locus *pfap2-g* (red) from the nearby internal virulence gene cluster (yellow) in stage II/III gametocytes. **c** Overall reduced number of intrachromosomal and interchromosomal interactions between *pfap2* TF genes and virulence genes in gametocytes and sporozoites as compared to the IDC stages. **d** Invasion gene clusters (blue bar) on chromosomes 2 (top) and 10 (bottom) interact with subtelomeric virulence genes (yellow bar) in gametocytes, but not during the IDC. In each plot, the top triangle shows the aggregated data of both gametocyte stages, while the bottom triangle shows the aggregated data from the three IDC stages. Bins that depict interactions between virulence genes and invasion genes are highlighted by a red box. **e** Increased number of intrachromosomal and interchromosomal interactions between invasion genes and virulence genes in gametocytes and sporozoites as compared to the IDC stages

Previous work has shown that knockdown of heterochromatin protein 1 (PfHP1) during the IDC results in activation of the gametocyte-specific transcription factor locus *pfap2-g* and an increased formation of gametocytes^26^. PfHP1 interacts with the repressive histone mark H3K9me3 on silenced *var* genes that are colocalized in a perinuclear heterochromatic compartment. Using DNA-FISH, we observed that the *pfap2-g* locus was located in close proximity to a subtelomeric *var* gene on chr8 in >90% of the cells observed (Fig. 2a and Supplementary Fig. 8A), suggesting that *pfap2-g* is associated with the repressive cluster during the IDC. In agreement with this observation, the trophozoite and schizont stage fit-hi-c *p*-value heatmaps showed a significant interaction between *pfap2-g* and the nearest internal virulence gene cluster (Fig. 2b and Supplementary Fig. 9A). The virulence cluster and *pfap2-g* locus each straddle two ten kilobase bins, and in trophozoites, each of the four possible pairwise interactions were significant (fit-hi-c *q*-value < 0.05). We performed virtual 4C at MboI restriction site resolution to demonstrate that this interaction was specific for *pfap2-g* and did not involve the nearby *pfap2* PF3D7_1222400 (Supplementary Fig. 10), although these two *ap2 TF* genes are located in neighboring 10 kb bins and therefore fall outside our limits to detect potential translocations. In addition, *pfap2-g* significantly interacted with virulence clusters on chromosomes 6 and 8 in trophozoites (fit-hi-c *q*-value < 0.05; Supplementary Data 4). In stage II/III gametocytes, no significant interactions between *pfap2-g* and virulence clusters were observed (*q*-values = 1.0; Fig. 2b; Supplementary Data 4; Supplementary Table 3), indicating that *pfap2-g* dissociates from the repressive cluster in the transition from the IDC to early gametocytes. Interactions between *pfap2-g* and virulence clusters were partially regained in the late gametocyte stage (Supplementary Data 4, Supplementary Table 3). Unfortunately, DNA-FISH experiments were unsuccessful for the gametocyte stage. An improved DNA-FISH methodology for the gametocyte stages will need to be developed to further confirm these results by an independent approach.

Significant contacts with virulence gene clusters at any parasite stage were observed for a total of 9 *pfap2* loci (Supplementary Table 3). The dissociation of *pfap2* genes from the repressive center in other stages than the IDC seemed to be a general trend: fewer significant contacts (*q*-value < 0.05) between *pfap2* genes and virulence gene clusters were observed in gametocytes and sporozoites as compared to the IDC stages (*p*-value = 0.0027, one sided sign test; Fig. 2c and Supplementary Fig. 9B). We used a resampling procedure (see Supplementary Information) to compare this result to randomly-selected bins; the *p*-value was 0.021. Note that *pfap2* PF3D7_0420300 is located in close genomic proximity to a virulence cluster in chromosome 4. To ensure that the significant decrease in *pfap2* gene loci interacting with virulence clusters is not due to this gene, we repeated the *p*-value calculation without PF3D7_0420300 and confirmed that this remained significant (*p*-value = 1.192e−07, one-sided sign test). Such changes in interactions with virulence gene clusters between stages were not observed for an unrelated gene family (histone genes, data not shown). These results are indicative of an important connection between genome organization and the activity of *pfap2* genes that drive parasite life cycle progression.

### Relocation of invasion genes during gametocytogenesis

Distinct changes were observed in chromosomes 2 and 10 at loci that harbor invasion genes (Fig. 2d). These genes encode proteins that are expressed in merozoites and mediate attachment to and entry of red blood cells and include several merozoite surface proteins, S-antigen, and glutamate-rich protein. In contrast to several other invasion genes (*pfrh4*^27^, *clag*, and *eba*^28^, these genes are not known to undergo clonally variant expression, and we therefore consider their association with heterochromatin in gametocytes to be a different regulatory mechanism than the epigenetic mechanisms that control expression of *pfrh4*, *clag*, and *eba* during the IDC. In gametocytes, these loci showed strong interaction with the subtelomeric regions, while these contacts were not observed in the IDC. To quantify this observation, we assessed the number of significant contacts between invasion gene loci and virulence clusters in each life cycle stage. A larger number of significant contacts were observed between invasion genes and virulence clusters in the transmission stages than in IDC stages (*p*-value < 2.2e−16, sign test; Fig. 2e and Supplementary Fig. 9C). The lack of interactions between invasion gene GLURP on chromosome 10 (PF3D7_1035300) and *var* gene PF3D7_0800300 during the IDC was confirmed by DNA-FISH (Supplementary Fig. 8B). As mentioned earlier, DNA-FISH experiments were unfortunately not successful for the gametocyte stage. Collectively, our data indicate that, similar to the *pfap2* gene family, the expression of invasion genes during the life cycle is correlated with association with or dissociation from repressive heterochromatin.

### Expansion of subtelomeric heterochromatin in gametocytes

**Fig. 3:**
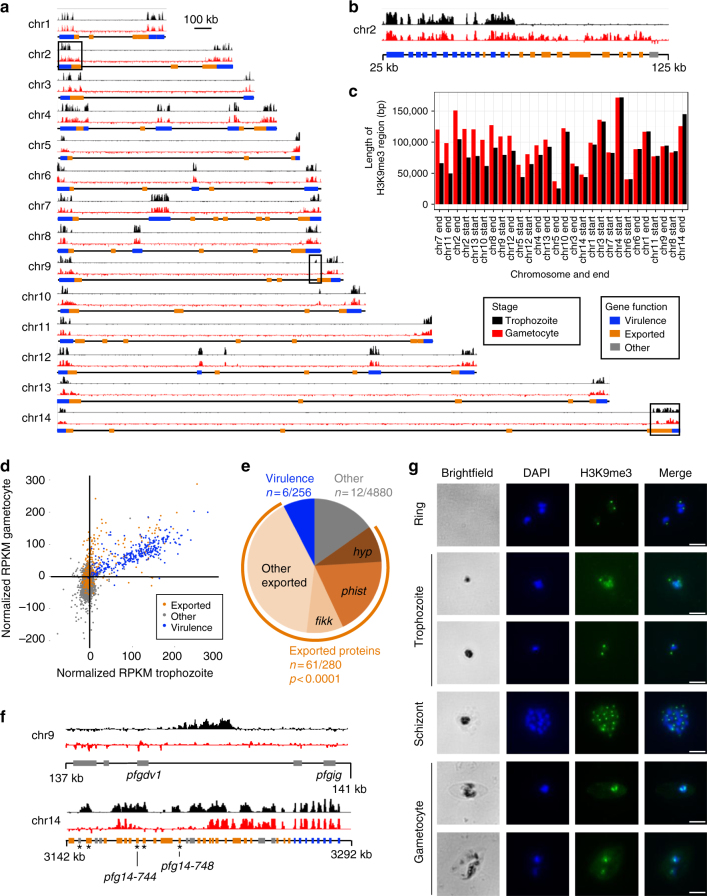
Silencing of genes encoding exported proteins in gametocytes through expansion of heterochromatin. **a** ChIP-seq analysis of genome-wide H3K9me3 localization in trophozoites (top tracks in black) and stage IV/V gametocytes (bottom tracks in red). Results of one representative biological replicate are shown for each stage. Results for a second biological replicate are shown in Supplementary Fig. 11. The regions depicted in panels B and F are indicated with black boxes. **b** Expansion of H3K9me3 heterochromatin in gametocytes as compared to trophozoites, predominantly to genes encoding exported proteins. **c** Length of each subtelomeric region in which the majority of genes is marked by H3K9me3, sorted by the difference in length between these regions in trophozoites and gametocytes. **d** H3K9me3 levels per gene at the trophozoite and gametocyte stages. **e** Enrichment of genes encoding for exported proteins among genes with increased levels of H3K9me3 in gametocytes (*p*-value from a two-tailed Fisher’s exact test). **f** Loss of H3K9me3 mark in gametocytes on chromosome 9 between gametocyte development genes *pfgdv1* and *pfgig*, as well as at gametocyte-specific genes encoding exported proteins on chromosome 14 (indicated with an asterisk). **g** Immunofluorescence analysis showing a single H3K9me3 focus in ring and schizont stages, and either one or two foci in gametocytes. Scale bar denotes 1 μm

To further study changes in chromatin organization during gametocytogenesis, we determined the distribution of repressive histone mark H3K9me3 in late ring/early trophozoites and stage IV/V gametocytes by performing ChIP-seq on two biological replicates for each stage (Supplementary Fig. 11A and Supplementary Data 5) that were combined for downstream analyses. We used two different commercially available anti-H3K9me3 antibodies to rule out that our ChIP-seq results were influenced by the antibody used. In trophozoites, H3K9me3 marking was restricted to subtelomeric regions, internal virulence gene clusters and a few additional loci (including *pfap2-g* and *dblmsp2*), as previously described for both H3K9me3^7,8^ and PfHP1^29^ (Fig. 3a and Supplementary Fig. 11B, C). These same regions were occupied by H3K9me3 in gametocytes. In addition, in several chromosomes, the subtelomeric heterochromatin marking expanded to more internally located genes in gametocytes (Fig. 3a–c and Supplementary Fig. 11B, C). The expansion of heterochromatin at the chromosome ends was also visible in the contact count heatmaps as larger subtelomeric domains that showed strong intra-domain interactions and were depleted of interactions with the internal region of the chromosome (Supplementary Data 1). While not all genes in these regions were marked by H3K9me3, a total of 79 genes showed increased H3K9me3 levels in gametocytes as compared to trophozoites, 61 of which were exported proteins that may play a role in erythrocyte remodeling (Supplementary Data 5). These genes included members of the *phist* (*n* = 15 out of a total of 68), *hyp* (*n* = 7 out of 34) and *fikk* (*n* = 7 out of 19) families, as well as 32 other genes annotated as exported proteins or containing a PEXEL export motif (Fig. 3d, e). Other members of gene families encoding exported proteins were marked with H3K9me3 in the IDC. However, 47 of these 61 genes have never been detected in a heterochromatic state in the IDC^7,8,29^. At two loci on chr9 and chr14, respectively, H3K9me3 was lost in gametocytes (Fig. 3f). The locus on chr9 is between two genes known to be involved in gametocyte differentiation (*pfgdv1* and *pfgig*) and is deleted in various gametocyte-defective *P. falciparum* strains^30^. On chromosome 14, the genes that were not marked by H3K9me3 in gametocytes encode exported proteins that have been implicated in gametocytogenesis: PF3D7_1476600, PF3D7_1477300 (*Pfg14-744*), PF3D7_1477400, and PF3D7_1477700 (*Pfg14-748*)^30^. These results imply that H3K9me3 and chromatin structure play important roles in gene activation and silencing during gametocyte formation. To validate the 3D modeling and heterochromatin clustering, we performed immunofluorescence imaging against repressive histone mark H3K9me3. We identified a single nuclear H3K9me3 focus per nuclei in rings and schizonts (Fig. 3g and Supplementary Fig. 12), corresponding to the single repressive center harboring all virulence genes as predicted by our 3D models. In trophozoites, the number of foci varied, in line with nuclear expansion^16,31^, and the progression of DNA replication that takes place at the end of this stage (Fig. 3g). Gametocytes showed either one or two H3K9me3 foci that did not seem to be associated with a male or female phenotype.

### Formation of superdomains on a *P. falciparum* chromosome

**Fig. 4:**
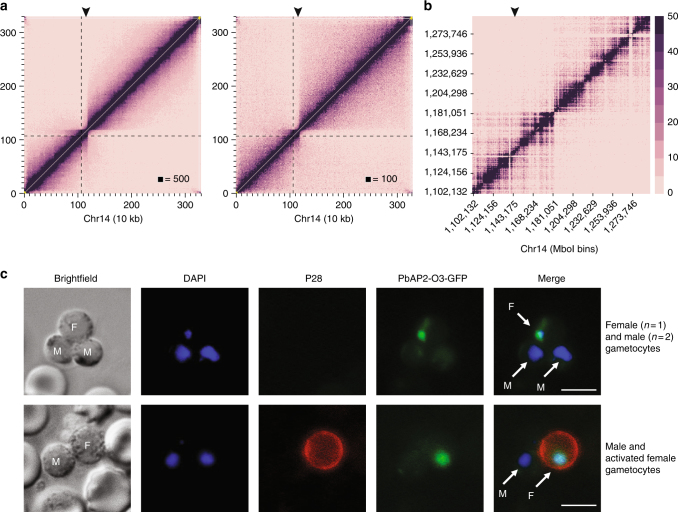
Formation of superdomains on chr14 in gametocytes. **a** ICE-normalized contact count heatmap at 10 kb resolution of early gametocyte (left) and late gametocyte (right) chromosome 14 showing the separation of the chromosome into two superdomains. The dashed line indicates the location of the centromere, and the arrowhead indicates the position of PF3D7_1429200. **b** Smaller region of chromosome 14 centered on the domain boundary that is located inside PF3D7_1430000, a conserved gene with unknown function. **c** The homolog of *pfap2* gene PF3D7_1429200 in *P. berghei* (PBANKA_1015500; *pbap2-o3*) has a nuclear localization in female gametocytes and gametes, but is not detected in male gametocytes. The top row shows male and female gametocytes. The bottom row shows a male and female gamete activated by mosquito ingestion, which triggers expression of the female-specific surface protein P28. Male (M) and female (F) parasite are indicated in the brightfield and merged images. Scale bar denotes 10 μm

Chromosome 14 showed the formation of a strong domain boundary in both early and late gametocytes, which was not observed in any of the other life cycle stages (Fig. 4a and Supplementary Data 1). This separation of the chromosome into two superdomains is reminiscent of the bipartite structure of the inactivated X chromosome (Xi) in human, rhesus macaque, and mouse^32–34^. Zooming in on the boundary region of *P. falciparum* chromosome 14 showed a sharp transition at the MboI restriction site at nucleotide position 1,187,169 (Fig. 4b and Supplementary Fig. 13). To demonstrate that this sharp boundary is not the result of a chromosomal translocation in the NF54 strain used for gametocyte isolations, we confirmed that the genomic region that spans the boundary region can be amplified by PCR and can be detected by Southern blot in both 3D7 and NF54 strains (Supplementary Fig. 14). In eukaryotic genomes, genes close to the domain boundary are often associated with higher levels of transcription^35,36^. The domain boundary is located inside or near PF3D7_1430100, which encodes serine/threonine protein phosphatase 2A activator (PTPA; Supplementary Fig. 15A). In humans, serine/threonine protein phosphatase 2A (PP2A) is one of the four major Ser/Thr phosphatases and is thought to play a complex, but mostly inhibitory role in the control of cell growth and division^37^. Gametocytes have a higher expression level of *pfptpa* than the IDC stages and express a different variant of *pfptpa* that does not contain exon 1 (Supplementary Fig. 16A). The sequence of intron 1 is unusual and contains many motifs that are repetitive (for example, 12 repeats of motif TGTACATACACTTAT and minor variations thereof, within the 705 nt intron; Supplementary Fig. 16B). These could be the binding sites for a lncRNA or protein involved in formation of the domain boundary.

The domain boundary is also relatively close to the *pfap2*-encoding locus PF3D7_1429200 (chr14:1,144,518–1,148,078) (Supplementary Fig. 15A). To evaluate whether this TF could be involved in sexual differentiation, we generated a transgenic *P. berghei* strain in which the homolog of PF3D7_1429200 (PBANKA_1015500) was expressed as a GFP-tagged protein (Supplementary Fig. 15B, C). *P. berghei* is widely used as a model for *P. falciparum*, in part because of the higher efficiency of genetic manipulations as compared to *P. falciparum*. Female gametocytes, activated female gametes, and zygotes all expressed the tagged ApiAP2 TF with nuclear localization of the protein, while the protein was completely absent in male gametocytes and gametes (Fig. 4c), as well as in IDC stages (data not shown). These results demonstrate that this ApiAP2 TF (named PfAP2-O3 hereafter in line with a recent publication^38^) is expressed in a strict sex-specific fashion.

### Rearrangement of chromosomes in sporozoites

Similar to the re-localization of invasion genes during the transition from the IDC to the gametocyte stage, the invasion genes also interacted more strongly with virulence genes in *P. falciparum* sporozoites than during the IDC (Fig. 2e and Supplementary Fig. 9C). In *P. vivax* sporozoites, a cluster of invasion genes on chromosome 10 showed depletion of interactions with other loci on the same chromosome as compared to surrounding genomic regions (Supplementary Fig. 17B). This observation may suggest that the invasion genes also have a distinct genome organization at this stage of the *P. vivax* life cycle. However, these results may also be caused by sequence variation in invasion genes in the field isolates used in this study as compared to the reference genome, resulting in lower mapping to this region.

**Fig. 5:**
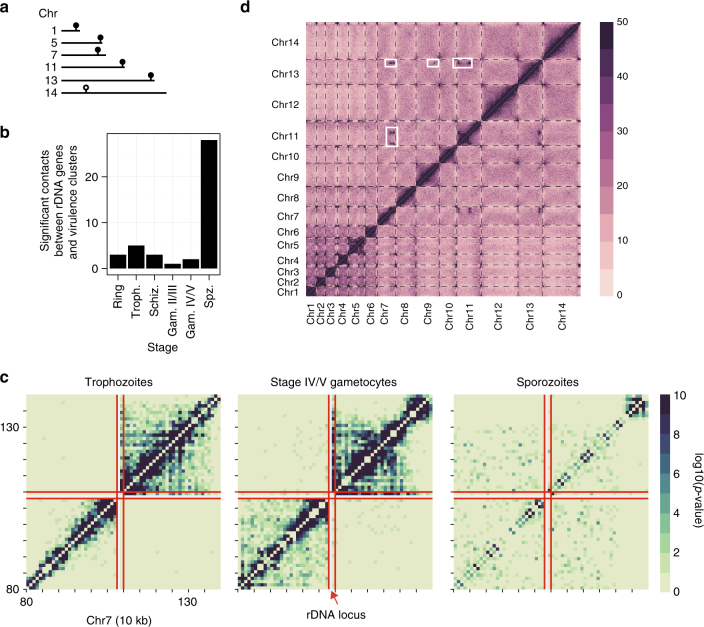
Changes in genome organization in salivary gland sporozoites. **a** Locations of rDNA genes in the *P. falciparum* genome. Units of 28S, 5.8S, and 18S genes on chromosomes 1, 5, 7, 11, and 13 are indicated with a filled symbol. Several additional rDNA genes are located on other chromosomes, including a unit of three 5S genes on chromosome 14, which is indicated with an open symbol. **b** Increased overall number of interactions between rDNA genes and virulence genes in *P. falciparum* sporozoites. **c** Loss of domain formation around the rDNA locus on chr7 in *P. falciparum* sporozoites as compared to other life cycle stages. The borders of the rDNA locus are indicated by red lines. **d** Strong interchromosomal interactions in *P. vivax* sporozoites, indicated by white rectangles. Dashed lines indicate chromosome boundaries

In addition, distinct changes were noticeable around rDNA loci. *P. falciparum* encodes four rDNA units containing single copies of the 28S, 5.8S, and 18S genes (Fig. 5a). The units on chromosomes 5 and 7 are active during the human blood stages, whereas the units on chromosomes 1 and 13 are active in the mosquito stages. In general, sporozoites showed a large increase in the number of contacts between rDNA genes and virulence genes as compared to the IDC stages and gametocytes (Fig. 5b and Supplementary Fig. 9D). These changes in conformation were most visible in chromosome 7, in which the rDNA unit is located at the boundary of two large domains in the IDC stages and gametocytes, which presumably contributes to its activation status. In sporozoites on the other hand, the separation of the chromosome into two large domains disappeared (Fig. 5c and Supplementary Fig. 4B). Similar changes in domain conformation can be observed around the rDNA locus on chromosome 5 (Supplementary Fig. 9E).

Prominent features of genome organization in *P. falciparum* and *P. vivax* sporozoites were strong long-range and interchromosomal contacts that involved other genes than virulence genes and that did not seem to be present in the IDC or gametocyte stages. In *P. vivax*, strong intrachromosomal contacts were present in chromosomes 7 and 11, which also formed strong interchromosomal interactions with each other and with additional loci on chromosomes 9 and 13 (Fig. 1b, rightmost panel, Fig. 5d, and Supplementary Table 4). For *P. falciparum*, intrachromosomal interactions were observed in chromosomes 3, 4, 8, 9, 11, 13, and 14 (Supplementary Data 1), although, interestingly, these are not homologous to the loci that participate in loops in *P. vivax*. Several of these loops involved *pfap2* loci and genes involved in sporozoite migration to the liver and in some cases in hepatocyte invasion, such as circumsporozoite protein (PfCSP), sporozoite micronemal protein essential for cell traversal (PfPLP1), thrombospondin-related anonymous protein (PfTRAP), sporozoite protein essential for cell traversal (PfSPECT1), and gamete egress and sporozoite traversal protein (PfGEST) (Table 1).

**Table 1:** Loci involved in long-range intrachromosomal interactions in *P. falciparum* sporozoites

Chr	Locus (kb)^a^	Gene	Description	Pv homolog
3	115	PF3D7_0302100	Ser/Thr protein kinase 1 (PfSTPK1)	PVX_119250
3	225	PF3D7_0304600	CS protein (PfCSP)	PVX_119355
4	245	PF3D7_0404500	6-cys protein (P52)	PVX_001020
4	375	PF3D7_0407600	Conserved, unknown function	n.a.
4	425	PF3D7_0408700	Perforin -Like Protein 1 (PfPLP1)	PVX_000810
8	135	PF3D7_0801900	Conserved, unknown function	PVX_093645
8	295	PF3D7_0805200 PF3D7_0805300	Gamete release protein (PfGAMER) Conserved, unknown function	PVX_093500 PVX_093495
9	125 135	PF3D7_0902800 PF3D7_0902900 PF3D7_0903000 PF3D7_0903100 PF3D7_0903200	Serine repeat antigen 9 (PfSERA9) Conserved, unknown function Conserved, unknown function PfRER1 PfRAB7	n.a. n.a. PVX_098595 PVX_098600 PVX_098605
9	325	PF3D7_0906600	Zinc finger protein	PVX_098775
9	535 545	PF3D7_0911700 PF3D7_0911800 PF3D7_0911900 PF3D7_0912000 PF3D7_0912100	GTP-binding protein Conserved, unknown function Falstatin (PfICP) Conserved, unknown function Zinc finger protein	PVX_099025 PVX_099030 PVX_099035 PVX_099040 PVX_099045
11	225	PF3D7_1105000 PF3D7_1105100 PF3D7_1105200	Histone H4 (PfH4) Histone H2B (PfH2B) WD repeat-containing protein (PfWRAP73)	PVX_090930 PVX_090935 PVX_090940
11	335	PF3D7_1107800	ApiAP2 TF	PVX_091065
13	1465	PF3D7_1335900	PfTRAP	PVX_082740
13	1675	PF3D7_1342500	PfSPECT1	PVX_083025
14	1875	PF3D7_1445600 PF3D7_1445700	RNA-binding protein Conserved, unknown function	PVX_118205 PVX_118200
14	2005	PF3D7_1449000	PfGEST	PVX_118040

Kb, kilobase; Pv, *Plasmodium vivax*; n.a., not available

^a^ All loci listed interact with all other listed loci in the same chromosome

### A putative clonally variant gene family in *P. vivax*

The genome annotation of *P. vivax* is less complete than that of *P. falciparum*, and many genes have been grouped into families based solely on sequence homology. An example is the *Pv-fam-e* (also named *rad*) gene family that is closely related to the *Plasmodium* helical interspersed sub-telomeric (*phist*) gene family^39^, encoding exported proteins involved in erythrocyte remodeling^40–42^. The *P. vivax* genome contains 45 *rad* genes, of which 10 and 27, respectively, are located in two separate clusters on chromosome 5. In the contact count matrix of *P. vivax* chromosome 5, the largest of the two clusters strongly interacted with the (sub-)telomeric regions and showed a depletion of interactions with all other intrachromosomal loci (Supplementary Fig. 17C). These results suggest that *P. vivax rad* genes may be regulated by organization into facultative heterochromatin.

### PfHP1 is essential for virulence gene colocalization

The clustering of virulence genes seems to be a general feature of the *P. falciparum* genome that is maintained throughout its life cycle. Recently, it was shown that depletion of PfHP1 results in loss of *var* gene repression and an arrest in parasite growth^26^, suggesting that this protein is essential for structural integrity of the repressive cluster. To study the effect of PfHP1 depletion on genome conformation, we performed Hi-C experiments on a transgenic *P. falciparum* strain expressing PfHP1 fused to GFP and a destabilization domain (DD), both in the presence and in the absence of Shield-1, resulting in expression or knockdown of the PfHP1 fusion protein, respectively^26^. Ring-stage parasites expressing the tagged PfHP1 protein showed a decrease in virulence gene clustering as compared to wild-type parasites (*p*-value = 0.026 and *p*-value ≤ 0.001, respectively, BH FDR-corrected Paulsen colocalization test^43^ (see Supplementary Information)). In particular, interactions between internal and subtelomeric *var* gene clusters were lost (Supplementary Fig. 18). This result is in agreement with an increase in internal *var* gene expression in this strain, as reported previously^26^. Virulence gene clustering was completely lost in the PfHP1-depleted strain (*p*-value = 0.129, BH FDR-corrected Paulsen colocalization test), in line with a generalized loss of *var* gene repression^26^. Accordingly, we observed more significantly changing virulence gene bins as compared with wild-type in the PfHP1-depleted strain (*n* = 71) than in the PfHP1-tagged strain (*n* = 18). These results confirm that PfHP1 is indeed essential for maintenance of the structure of the repressive cluster and thus for regulation of virulence gene expression.

### The 3D genome structure correlates with gene expression

**Fig. 6:**
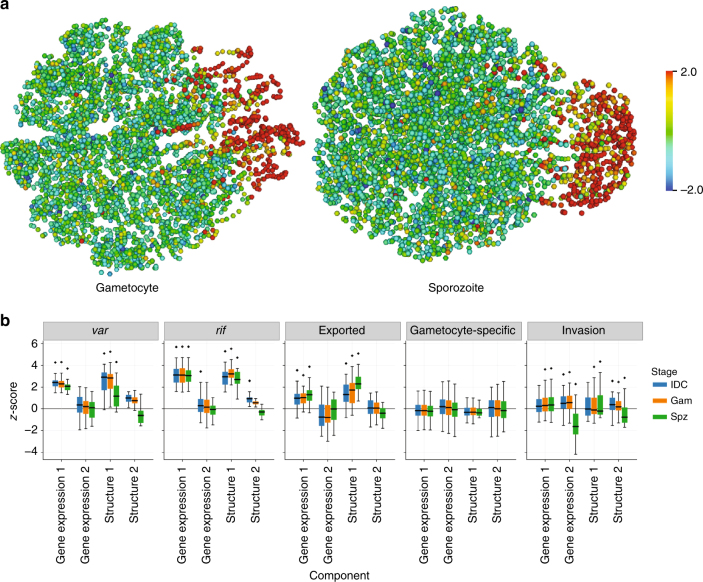
3D genome structure correlates with gene expression. **a** Each gene is plotted by its position within the 3D structure and is colored by its standardized KCCA score (see Supplementary Information) in the first gene expression component. The direction of this first gene expression component is from the telomere cluster on the right to the opposite side of the nucleus and is dominated by the repressive center (genes colored in red). **b** The distribution of standardized KCCA scores for the first and second gene expression components and the first and second structure components of specific groups of genes. The box plots show the median, first and third quartile, and the minimum and maximum values. Diamonds indicate groups of genes for which the standardized KCCA scores for both the gene expression component and the structure component were significantly different (*t*-test, FDR < 0.1%)

Finally, we explored the relationship between gene expression and 3D structure, leveraging four published expression data sets^44–47^ and our 3D models of the genome architecture. As in our previous study, we applied kernel canonical correlation analysis (KCCA)^48^. KCCA is an unsupervised learning approach akin to principal component analysis that identifies a set of orthogonal gene expression components that are coherent with the 3D structure. To perform this analysis, we separated the 3D models into three distinct groups: those related to IDC (ring, trophozoite, schizont), gametocyte (early and late) and sporozoite stages. For each of these groups of time points, we extracted a gene expression component and a structure component that exhibited coherence to the expression profiles and 3D structure, such that genes whose expression is correlated with the selected gene expression component tend to be colocalized in 3D. The gene expression components for the three sets of structures were highly correlated and were dominated by the repressive center (Fig. 6a and ref. ^16^). To further interpret the results of the KCCA, we extracted ranked lists of genes based on their KCCA scores: lists that rank genes based on the similarity of their gene expression profiles with the first or second gene expression components, and lists that rank genes based on the similarity of their 3D position with the first or second structure components. We then investigated whether several sets of genes were enriched in those ranked lists. *Var*, *rifin*, and exported protein genes all showed strong and significant enrichment on the first gene expression and structure components for all stages (Fig. 6b), as expected based on their localization in or near the repressive center. In contrast, the invasion genes were significantly enriched for the first gene expression component in gametocytes and sporozoites, and for the second gene expression and structure components in all stages (Fig. 6b). While the average scores for this group of genes are relatively small, these results suggest that expression of these genes is also coordinated with their location within the nucleus. Gametocyte-specific genes did not show correlation to the first or second component for either gene expression or structure, which is in line with regulation of these genes by other factors, such as the gametocyte-specific transcription factor PfAP2-G, instead of localization within the nucleus.

## Discussion

Understanding the mechanisms involved in gene regulation during the various life cycle stages of *P. falciparum* will be important for the development of novel strategies to block parasite replication and transmission. Our previous study described the hallmarks of genome organization during the intra-erythrocytic developmental stages of *P. falciparum*, and showed that nuclear architecture correlated well with gene expression^16^. Here, we investigated chromosome conformation and chromatin structure in the stages of parasite transmission from human to mosquito (gametocytes) and from mosquito to human (sporozoites) and compared all stages to identify various subsets of genes that exhibit changes in genome organization during the complex life cycle of *Plasmodium* parasites.

Our results confirm that virulence gene families, such as *var, rifin*, and *stevor*, cluster in perinuclear heterochromatin, not only during the IDC stages, but also in gametocytes and sporozoites. Previous reports showed multiple virulence gene clusters scattered around the nucleus, either by DNA-FISH on telomere repeat sequences^12,13^, IFA on H3K9me3^7^ or IFA on proteins that bind to chromosome ends (PfSIP2-N^49^) or subtelomeric regions (PfHP1^26,49^). Our population-based Hi-C data showed strong interactions between all telomeres, which could be consistent with a completely random distribution of telomeres over multiple clusters. However, our IFA results conflict with this large body of data and instead show a single H3K9me3 focus in all blood stages in which the parasite is not undergoing DNA replication, representative of a single repressive center. Similar results were previously obtained by IFA against the heterochromatin mark H3K36me3^11^ and also recently for heterochromatin mark H3K9me3^50^. Differences in timing during the cell cycle (before or after start of DNA replication) may account for some of these differences. Indeed, we also observed multiple H3K9me3 foci during the trophozoite stage, most likely as a result of nuclear expansion and the start of DNA multiplication. We currently do not have an explanation that would reconcile our observations with those of others in the field. However, whether *var* genes cluster in one or multiple repressive centers throughout the IDC, both models are consistent with the presence of distinct heterochromatin and euchromatin regions controlling gene expression and antigenic variation in *P. falciparum*.

In gametocytes, the overall genome organization is similar to IDC stages, in line with relatively small changes in the gene expression program during gametocytogenesis^51^. We observed specific changes for *pfap2-g*^3^ that dissociates from the repressive center, and for invasion genes that associate with the repressive center. In addition, subtelomeric genes encoding exported proteins are selectively silenced or activated by H3K9me3 deposition or removal, respectively. These results confirm that remodeling of the infected host cell is among the essential changes that occur during gametocytogenesis^52,53^, and that an epigenetic switch provides an extra layer of transcriptional regulation for the genes involved. The presence of H3K9me3 in the relatively large intergenic region downstream of *pfgdv1* suggests that this region by itself is an important determinant in the regulation of gametocyte differentiation. A lncRNA is transcribed from this region at low levels during the IDC^54^, and it is tempting to speculate that this transcript is upregulated in gametocytes and possibly essential for gametocyte development.

The sharp division of chromosome 14 into two superdomains is an intriguing finding that will need to be explored in more detail. The inactivated X chromosome (Xi) in mammalian cells adopts a seemingly similar structure with a ~200 kb hinge region that is organized into euchromatin, while the surrounding chromosome is in a heterochromatic state^55^. However, the domain boundary on *P. falciparum* chromosome 14 is sharp, does not have a distinct hinge region, and is not surrounded by H3K9me3 marking. In support of the hypothesis that boundary formation is sex-specific, we discovered that the homolog of ApiAP2 transcription factor PF3D7_1429200 (PfAP2-O3) in *P. berghei* is strictly female-specific, suggesting that PfAP2-O3 controls female gametocyte differentiation. A few hundred genes are differentially expressed between male and female gametocytes^56,57^ and could be under the control of this protein, either through direct transcriptional regulation or through selective stabilization of female-specific transcripts. Interestingly, disruption of *ap2-o3* in *P. berghei* resulted in differential gene expression in gametocytes and reduced gametocyte levels, although the sex ratio was not influenced^38^. However, *pfap2-03* is located approximately 40 kb from the domain boundary and it is not directly clear if and how expression of this gene would be influenced by the formation of these superdomains.

An alternative hypothesis is that the domain boundary is involved in regulation of the nearby gene Pf3D7_1430100 (*pfptpa*). PfPTPA has been shown to bind and activate PP2A, and to block the G2/M transition^58^. The transition from rapidly dividing asexual parasites into cell cycle arrested gametocytes is likely to require tight cell cycle regulation by a protein such as PTPA. Given the differences in *pfptpa* expression between the IDC and gametocyte stages, we speculate that the domain boundary inside or close to this gene may be important for driving the expression of this gene, which may in turn activate PP2A to block cell division in gametocytes. In ongoing efforts, we are further investigating the role of the domain boundary in gene expression and gametocyte differentiation.

From an evolutionary perspective, *P. falciparum* and *P. vivax* are highly divergent within the family of *Plasmodium* species^59,60^. Salivary gland sporozoites maintain a relatively quiescent transcriptional state, waiting for injection into the human bloodstream and invasion of a hepatocyte before ramping up transcriptional activity. Our interpretation of the numerous long-range interactions in the sporozoite stage is that the majority of the genome is transcriptionally repressed, with the exception of several active loci that colocalize in transcriptional islands, giving rise to long-range interactions. The observation that the genes involved in these long-range interactions are not each other’s homologs in *P. falciparum* and *P. vivax* is suggestive of species-specific gene expression and warrants further investigation.

To fully understand transcriptional regulation, it is of great interest to unravel the causal relationship between genome organization and transcriptional activity. In multicellular organisms, evidence is accumulating that certain aspects of genome organization are independent of transcription^61^. In addition, disruptions in genome structure that bring together previously isolated promoters and enhancers can result in gene activation^62^. It will be important to determine to what extent genome organization controls transcriptional activity in *P. falciparum*. Our findings bring a new level of insight into genome dynamics during the *Plasmodium* life cycle and open up new avenues for targeted approaches towards understanding parasite gene regulation. In addition, molecules inhibiting the (re-)structuring of the genome have the potential to act as potent transmission-blocking antimalarials.

## Methods

### Experimental procedures

Parasite strains and cultures: The *P. falciparum* strain NF54 (obtained from the MR4 malaria repository) was cultured at 5–10% parasitemia in human O^+^ erythrocytes at 5% haematocrit. The induction of gametocyte-stage parasites was performed by sorbitol synchronization and culturing in a low medium volume (see Supplementary Methods). Stage IV/V gametocytes at 2% parasitemia were isolated from 150 ml of culture using a percoll gradient, were cultured for one additional day, and were then isolated by magnetic purification yielding 6.25 × 10^8^ parasites (Supplementary Fig. 1). To obtain sporozoites, adult female *Anopheles stephensi* mosquitoes were allowed to feed on *P. falciparum* NF54 gametocyte cultures. Sporozoites were harvested from infected mosquitoes 14–19 days later. Stage II/III gametocytes were obtained using the *P. falciparum* NF54^Pfs16^ reporter gene line^63^. Gametocytes were isolated by magnetic purification, yielding 1.17 × 10^8^ parasites with high purity (>95% gametocytes) as determined by GFP expression (Supplementary Fig. 1). To obtain *P. vivax* sporozoites, female *Anopheles cracens* mosquitoes were fed on blood samples drawn from *P. vivax* infected patients who had given written informed consent and who attended a Shoklo Malaria Research Unit (SMRU) clinic in Mawker Thai or Wang Pha, on the western Thailand-Myanmar border. The research protocol was approved by Oxford Tropical Research Ethics Committee and adhered to the Declaration of Helsinki. Two biological replicates containing 21,583,075 and 29,245,000 sporozoites, respectively, were used for Hi-C experiments. Fifteen days post-infection, *P. vivax* sporozoites were harvested from *An. cracens* salivary glands. The construction of *P. falciparum* 3D7 transgenic strain PfHP1-GFP-DD has been described previously^26^. Parasites were synchronized, split into two populations at 4–12 hpi and cultured in the presence or absence of Shield-1 as described. Parasites were harvested for Hi-C at 4–12 hpi in the next cell cycle. More details about parasite cultures and collections are provided in the Supplementary Methods.

Hi-C procedure: Parasites were crosslinked in 1.25% formaldehyde in warm PBS for 25 min on a rocking platform in a total volume between 1 and 10 ml, depending on the number of parasites harvested. Glycine was added to a final concentration as 150 mM, followed by 15 min of incubation at 37 °C and 15 min of incubation at 4 °C, both steps on a rocking platform. The parasites were centrifuged at 660×*g* for 20 min at 4 °C, resuspended in 5 volumes of ice-cold PBS, and incubated for 10 min at 4 °C on a rocking platfrom. Parasites were centrifuged at 660×*g* for 15 min at 4 °C, washed once in ice-cold PBS, and stored as a pellet at −80 °C. For late stage gametocytes, the crosslinking protocol was slightly modified. Late gametocytes were collected in lysis buffer (25 mM Tris-HCl, pH 8.0, 10 mM NaCl, 2 mM 4-(2-aminoethyl)benzenesulfonyl fluoride HCl (AEBSF), 1% Igepal CA-360 (v/v), and EDTA-free protease inhibitor cocktail (Roche)) and incubated for 10 min at RT. After homogenization by 15 needle passages, formaldehyde was added to a final concentration of 1.25%, followed by 10 more needle passages. The protocol was then continued as for all other samples. To map the inter-chromosomal and intrachromosomal contact counts, crosslinked parasites were subjected to the tethered conformation capture procedure, using MboI for restriction digests, as described in detail in the Supplementary Methods.

DNA-FISH: DNA-FISH experiments were performed as previously described^16^. In brief, probes were prepared using Fluorescein-High Prime and Biotin-High Prime kits (Roche) according to manufacturer’s instructions. Template DNA was prepared by PCR (5 min at 95 °C, 35 cycles of 30 s at 98 °C, followed by 150 s at 62 °C, and 5 min at 62 °C) using the KAPA HiFi DNA Polymerase HotStart ReadyMix. Sequences of primers used for probe generation are shown in Supplementary Table 5. Double sorbitol synchronized ring-stage parasites were extracted using 0.015% saponin in cold PBS, washed in cold PBS and fixed in 4% formaldehyde in PBS at RT. A monolayer of parasites was deposited on a 9 × 9 mm frame-seal slide chamber on a standard microscopy slide and air-dried. Parasites were permeabilized with 0.1% Triton X-100 in PBS. After application of the denatured probes, the slides were denatured at 80 °C for 30 min followed by hybridization at 37 °C overnight. The slides were washed, equilibrated, washed in TNT solution, stained with DAPI and mounted.

Immunofluorescence microscopy: *P. falciparum* IDC-stage parasites and gametocytes were fixed onto slides using 4% paraformaldehyde for 30 min at RT. Slides were washed three times using 1× PBS. The parasites were permeabilized with 0.1% Triton-X for 30 min at RT, followed by a wash step with 1× PBS. Samples were blocked overnight at 4 °C in IFA buffer (2% BSA, 0.05% Tween-20, 100 mM glycine, 3 mM EDTA, 150 mM NaCl and 1× PBS). Cells were incubated with anti-Histone H3 antibody (ab8898 (Abcam), 1:500 or 07–442 (Millipore), 1:500) for 1 h at RT followed by anti-rabbit Alexa Fluor 488 (Life Technologies A11008; 1:500). No differences were observed in the results obtained with the two primary antibodies (Supplementary Fig. 12). Slides were mounted in Vectashield mounting medium with DAPI. Images were acquired using an Olympus BX40 epifluorescence microscope.

H3K9me3 ChIP-seq: Asexual parasites were crosslinked for 10 min with 1% formaldehyde in PBS at 37 °C, while gametocytes were crosslinked with 1.25% formaldehyde in lysis buffer for 25 min at RT. Chromatin was sheared using the Covaris Ultra Sonicator (S220). ChIP was performed using 2 μg of anti-Histone H3K9me3 antibody (ab8898 (Abcam) for biological replicates #1 and 07–442 (Millipore) for biological replicates #2) or no antibody as a negative control. Details are provided in the Supplementary Methods.

Amplification and Southern blot of region spanning the domain boundary on chr14: Genomic DNA was isolated from a mixed blood stage culture of *P. falciparum* strain 3D7, from a mixed blood stage culture of *P. falciparum* strain NF54, and from a stage II/III gametocyte culture of *P. falciparum* strain NF54 using the DNeasy Blood & Tissue kit (Qiagen). PCR amplifications were performed using 50 ng of genomic DNA, 0.4 μM of each primer (see Supplementary Table 5), and HiFi HotStart ReadyMix (KAPA Biosystems) with the following program: 5 min at 95 °C, 30 cycles of 30 s at 95 °C, 30 s at 52 °C, 2 min at 62 °C and a final extension of 6 min at 62 °C. Genomic DNA (100 ng) was digested with restriction enzyme DraIII (NEB), purified using the DNA genomic DNA Clean & Concentrator kit (Zymo Research), and separated by DNA electrophoresis on a 0.8% agarose gel (100 min at 35 V). The DNA was transferred to charged nylon membrane by upward capillary transfer. The DNA probe was generated by PCR amplification using 10 ng of genomic DNA, 0.4 μM of each primer (see Supplementary Table 5), 0.425 mM biotinylated dCTP, and HiFi HotStart ReadyMix (KAPA Biosystems) with the following program: 3 min at 95 °C, 35 cycles of 20 s at 98 °C, 30 s at 58 °C, 30 s at 62 °C and a final extension of 5 min at 62 °C. The membrane was incubated with 25 ng/ml probe in PerfectHyb Plus hybridization buffer (Sigma) overnight at 60 °C. The membrane was blocked with 5× Casein blocking buffer (Sigma) and incubated with HRP-conjugated streptavidin (1:2000). The membrane was developed using Amersham ECL Prime Western Blotting Detection Reagent (GE Healthcare).

Validation of sex-specific ApiAP2 TF in *P. berghei:* Transgenic parasites endogenously expressing a GFP-tagged version of the *P. berghei* homolog of Pf3D7_1429200 (PBANKA_1015500) were generated by single homologous recombination^64^. A 1323 bp region of PBANKA_1015500 omitting the stop codon was PCR amplified on genomic DNA using primers T2191 (5′-CCCCGGTACCGAATGCCCTAATAAATCTATTTCAATAG, KpnI site underlined) and T2192 (5′-CCCCGGGCCCATATTTTTTTGGTCGTTGGAAATTAAAC, ApaI site underlined). This was inserted upstream of the *gfp* sequence in the p277 vector using KpnI and ApaI restriction sites as underlined in the primers. The p277 vector contains the human *dhfr* cassette, conveying resistance to pyrimethamine. Before transfection, the sequence was linearized using ClaI. For the endogenously C-terminal fusion GFP-tagged parasites, a diagnostic PCR reaction was used as illustrated in Supplementary Fig. 15C. Primers INT T219 (5′-CAAATGATATTATCCCTTATATTGAAAG) and ol492 (5′-ACGCTGAACTTGTGGCCG) were used to determine correct fusion of the gfp sequence at the targeted locus by single homologous recombination. The presence of the full-length gene was verified using primers INT T219 and T2192. Asexual proliferation and gametocytogenesis were analysed using blood smears. Gametocyte activation and zygote formation were monitored using in vitro cultures and the surface antigen P28. Blood stages were stained in schizont medium for 60 min at 37 °C. Cells were washed once with respective medium and resuspended in PBS containing Hoechst 33342 DNA stain before being mounted for fluorescent microscopy. Six-to-eight-week-old female Tuck-Ordinary (TO) or NIHS (Harlan) outbred mice were used for all experiments. All animal work at Nottingham has passed an ethical review process and was approved by the United Kingdom Home Office. Work was carried out in accordance with the United Kingdom “Animals (Scientific Procedures) Act 1986” and in compliance with “European Directive 86/609/EEC” for the protection of animals used for experimental purposes under UK Home Office Project Licenses (30/3248). Sodium pentabarbitol was used for terminal anesthesia and a combination of ketamine followed by antisedan was used for general anesthesia.

### Computational methods

We mapped and binned reads and created and ICE-normalized contact count matrices as previously described (ref. 16 and Supplementary Information). A consensus 3D genome structure was inferred for each of the transmission stages and the three IDC stages using Pastis^20^. Pastis uses a maximum likelihood approach based on modeling contacts using a negative binomial model to account for the observed overdispersion. This method generates more accurate and robust structures when compared to the optimization-based approach we used previously^16^. To identify significant contacts, we modeled the effect of genomic distance on contact count probability with a spline using fit-hi-c^19^. Because of the possibility of differing statistical power between datasets, we report both the number of significant contacts above a given threshold, as well as the percent of significant contacts meeting a criterion (e.g., between *pfap2* gene loci and virulence clusters) out of all significant contacts. Significant co-localization of gene sets was assessed using previously developed tests (refs.^21,65^, and Supplementary Information).

We developed ACCOST (altered chromatin conformation statistics) to estimate the statistical significance of differences in contact counts between samples, taking as inspiration the negative binomial-based tests used for RNA-seq data^66–68^. Briefly, we modeled each bin as a negative binomial random variable, and we estimated the relationship between the mean and variance by grouping pairs of loci that are separated by the same linear genomic distance. We adapted the model employed by DESeq to Hi-C data by using an explicit specific scaling factor corresponding to bin-specific ICE biases. In addition, we estimated variance and dispersion of the negative binomial without replicates by assuming that most bins at a given genomic distance act similarly. Contact count matrices were subsampled to account for differences in interaction counts measured at the various stages and only intrachromosomal contacts for which the sum of the contacts was above the 80% percentile were tested. The resulting fold-change values were filtered after FDR estimation to only include loci that show a two-fold or larger difference in contacts, after normalizing for the effect of genomic distance, with a false discovery rate of less than 1% (Supplementary Data 3). For details of the model and implementation, see Supplementary Information.

### Code availability

Python scripts for mapping, binning, and normalization are described in the Supplementary Information and source code is available from Bitbucket (https://bitbucket.org/noblelab/plasmo-hic-2018/). ICE normalization was done using iced (https://github.com/hiclib/iced). The source code for ACCOST is available at https://github.com/cookkate/ACCOST.

### Data availability

The Hi-C and ChIP-seq sequencing data that support the findings of this study have been deposited in the NCBI Sequence Read Archive with accession numbers SRP091967 and SRP091939, respectively. Fold-change heatmaps can be accessed at http://noble.gs.washington.edu/proj/plasmo3d_sexualstages/.

## Electronic supplementary material

Supplementary Information

Description of Additional Supplementary Files

Supplementary Data 1

Supplementary Data 2

Supplementary Data 3

Supplementary Data 4

Supplementary Data 5

Supplementary Movie 1
