# Supplementary material for: Changes in genome organization of parasite-specific gene families during the Plasmodium transmission stages
Source: Nat Commun. 2018 May 15;9:1910. doi: 10.1038/s41467-018-04295-5 (PMC5954139; doi:10.1038/s41467-018-04295-5)
Supplement: Supplementary file 1 — Supplementary Information [file 41467_2018_4295_MOESM1_ESM.pdf]

**Supplementary Information to:**

# Changes in genome organization of parasite-specific gene families during the *Plasmodium* transmission stages

Bunnik et al.

## Supplementary Methods

### *Parasite strains and cultures*

The *P. falciparum* strain NF54 was cultured in human $O^+$ erythrocytes at 5% haematocrit as previously described[1]. The induction of gametocyte-stage parasites was adapted from a previously published protocol[2]. In brief, parasites were synchronized by 5% sorbitol lysis and diluted the following day into 75 $cm^2$ flasks to reach 0.5% parasitemia at a haematocrit of 8.3% (total volume of 15 ml). Parasites were stressed for 3 days by daily replacement of 10 ml of culture media. Cultures with 5-10% parasitemia were then induced by increasing media to a final volume of 25 ml per flask. For the next 5 days, cultures were maintained by removing 10 ml of media and adding 10 ml of fresh media supplemented with 50 mM N-acetyl glucosamine (NAG) to extinguish asexual parasites. Subsequently, cultures were fed with regular media until harvest of gametocytes at 2% parasitemia 14 days after induction, corresponding to stage IV-V of gametocytogenesis. Parasite developmental stages were assessed by Giemsa-stained blood smears. Gametocytes were isolated using a percoll gradient, were cultured for one additional day, and were then isolated by magnetic purification yielding 6.25 x $10^8$ parasites (**Supplementary Fig. 1**).

Stage II/III gametocytes were obtained using the *P. falciparum* $NF54^{Pfs16}$ reporter gene line, which expresses a green fluorescent protein (GFP)-luciferase fusion under the temporal control of the gametocyte-specific *Pfs16* promoter[3]. Asexual parasites were cultured as previously described[1], with some modifications[4]. Gametocyte production was carried out according to an established protocol[5], with minor modifications[4]. Gametocytes were isolated by magnetic purification, yielding 1.17 x $10^8$ parasites with high purity (>95% gametocytes) as determined by GFP expression. (**Supplementary Fig. 1**). Of note, we obtained zero mapped sequence reads for chr4:1-40,000 and chr5:1,310,000-1,343,557 in this transgenic strain, suggesting that these regions are deleted.

To obtain *P. falciparum* (strain NF54) sporozoites (**Supplementary Fig. 1**), asexual cultures in were maintained *in vitro* through infections of washed, type O+ erythrocytes grown in RPMI 1640 supplemented with 50 µM hypoxanthine, 25 mM HEPES, 2 mM L-glutamine, and 10% O+ human serum in a gas mixture consisting of 5% CO2, 5% O2, and 90% N2. Gametocyte cultures were initiated at 5% hematocrit and 1% parasitemia and were maintained for up to 17 days with daily media changes to promote sexual development. Adult female *Anopheles stephensi* mosquitoes (3 to 7 days post-emergence) were collected into mesh-topped, wax-lined pots and were allowed to feed through a membrane feeding apparatus for up to 20 min upon gametocyte cultures supplemented to 40% hematocrit containing fresh O+ human serum and O+ erythrocytes. Infected mosquitoes were maintained for 14 to 19 days at 27 °C and 75% humidity and were provided with a 20% w/v dextrose solution. Salivary glands from *P. falciparum*-infected mosquitoes were harvested by microdissection, homogenized, and total sporozoite numbers were counted on a hematocytometer. Sporozoite preparations were cleaned by purification on an Accudenz discontinuous gradient as previously described[6], washed with PBS and resuspended in PBS. For each experiment, sporozoite viability was assessed by incubation with 100 micrograms/ml of propidium iodide for 10 minutes at RT,

followed by the addition of 1 ml of DMEM. Sporozoites were pelleted in a microcentrifuge (15,000 rpm, 4 minutes at 4°C) and then resuspended in 20 microlitres of DMEM. PI-stained sporozoites were counted by fluorescence microscopy. In general, about 10% of sporozoites took up the dye.

To obtain *P. vivax* sporozoites, 10 ml of *P. vivax* blood samples were drawn from patients who had signed a consent form (protocol approved by Oxford Tropical Research Ethics Committee) and who attended a Shoklo Malaria Research Unit (SMRU) clinic in Mawker Thai or Wang Pha, on the western Thailand-Myanmar border. The samples were transported to the SMRU laboratories in Mae Sot and processed as described previously[7]. Hungry *Anopheles cracens* female mosquitoes (4-7 days old) were collected in small cups and blood fed with the infected blood using the Haemotek® membrane system. Fully engorged mosquitoes were moved to large plastic containers covered with a netting material and kept in secure incubators (Sanyo®, MIR-254) programmed at 26°C. After 7 days, 5 mosquitoes were dissected under a stereomicroscope to detect the presence of oocysts in the midguts. The average number of oocysts ranged from 100 to 200. Fifteen days post-infection, *P. vivax* sporozoites were harvested from An. cracens salivary glands in 50 µl of RPMI. Salivary glands were spun down, crashed with a 100 µl pipette and 10 µl of sporozoites suspension was placed on a KOVA® Glasstic slide with 10 grids. The number of parasites was counted and averaged. The two biological replicates contained 21,583,075 and 29,245,000 sporozoites, respectively.

The construction of *P. falciparum* 3D7 transgenic strain HP1-GFP-DD has been described previously[8]. Parasites were synchronized, split into two populations at 4-12 hpi and cultured in the presence or absence of Shield-1 as described. Parasites were harvested for Hi-C at 4-12 hpi in the next cell cycle.

*Tethered conformation capture procedure (Hi-C)*

**Day 1**: Parasite pellets were thawed on ice in 500 µl HiC lysis buffer (25 mM Tris-HCl at pH 8.0, 10 mM NaCl, 2 mM AEBSF, Roche Complete Mini EDTA-free protease inhibitor cocktail, 0.25% Igepal CA-630). Parasite membranes were disrupted by passing the lysate through a 26.5-gauge needle 15 times with a syringe. Samples were spun at 2,500 × g for 5 min at room temperature (RT). Pellets were washed twice with 250 µl ice-cold wash buffer (50 mM Tris-HCl at pH 8.0, 50 mM NaCl, 1 mM EDTA) and resuspended in the same buffer to a final volume of 50 µl. Samples were mixed with 19 µl 2% SDS to a final concentration of 0.5% and incubated at 55°C for 15 min. Suspensions were cooled down to RT before they were mixed with 21 µl 25 mM EZ-link Iodoacetyl-PEG2-Biotin (IPB) (Pierce) to biotinylate proteins. After incubating for 1 h at RT while rotating, the SDS was neutralized by adding 260 µl 1× NEBuffer 2 (NEB). Samples were mixed with 45 µl 10% Triton X-100 to a final concentration of 1% and incubated for 10 min on ice, followed by 10 min at 37°C. One µl 1 M DTT, 20 µl 10× NEBuffer 2, 83 µl water and 7 µl MboI restriction enzyme (NEB) (25 units/µl) was added to digest the DNA overnight at 37°C in a total volume of 506 µl.

**Day 2**: After digestion, samples were loaded into a 20 kDa cutoff Slide-A-Lyzer® Dialysis Cassette (Pierce) and dialyzed for 4 h at RT against 0.5 L of dialysis buffer (10 mM Tris-HCl at pH 8.0, 1 mM EDTA) to eliminate excess IPB remaining from the biotinylation step. Dialysis buffer was renewed after 3 h. Eighty µl MyOne Streptavidin T1 beads (Invitrogen) were washed 3 times with PBS + 0.01% Tween-20 (PBST) and beads were resuspended in 400 µl PBST. Dialyzed samples were transferred to 1.7 ml prelubricated microcentrifuge tubes (Corning). Four hundred µl beads were added and samples were incubated for 30 min at RT while rotating. To prevent interference of unbound streptavidin on the beads with later steps (adding biotinylated dCTP) 5 µl neutralized IPB was added to each tube. IPB was neutralized by adding an equimolar amount of 2-mercaptoethanol. Samples were incubated for an additional 15 min at RT while rotating. Not biotinylated chromatin and not cross-linked DNA was removed by washing the magnetic T1 beads once with 600 µl PBST and once with 600 µl wash buffer (10 mM Tris-HCl at pH 8.0, 50 mM NaCl, 0.4% Triton X-100). Beads were resuspended in 100 µl of the same wash buffer. MboI generated 5' overhangs were filled in by adding 63 µl water, 1 µl 1 M MgCl, 10 µl 10× NEBuffer 2, 0.7 µl 10 mM dATP, 0.7 µl 10 mM dTTP, 0.7 µl 10 mM 2'-Deoxyguanosine-5'-O-(1-thiotriphosphate). sodium salt, Sp-isomer (Axxora), 15 µl 0.4 mM Biotin-14-dCTP (Invitrogen), 4 µl 10% Triton X-100 and 5 µl 5U/µl DNA Polymerase I, Large (Klenow) Fragment (NEB). Samples were incubated for 40 min at RT while rotating. Reaction was stopped by adding 5 µl 0.5 M EDTA to the suspension. After 2 min of incubation at RT while rotating, beads were washed twice with 600 µl buffer (50 mM Tris-HCl at pH 7.4, 0.4% Triton X-100, 0.1 mM EDTA) and resuspended in 500 µl of the same buffer. Each sample was transferred into a 15 ml centrifuge tube. For blunt-end ligation under dilute conditions 500 µl sample was mixed with 4 ml water, 250 µl 10× Ligase Buffer (NEB), 100 µl 1 M Tris-HCl at pH 7.4, 90 µl 20% Triton X-100, 50 µl 100× BSA and 2 µl 2,000 U/µl T4 DNA Ligase (NEB), and incubated overnight at 16°C.

**Day 3**: The ligation reaction was stopped by adding 200 µl 0.5 M EDTA. The magnetic T1 beads were collected on the wall of the tube using a magnet and the solution was aspirated out of the tube. The beads were resuspended in 400 µl extraction buffer (50 mM Tris-HCl at pH 8.0, 0.2% SDS, 1 mM EDTA, 500 mM NaCl) and the mix was transferred into a new microcentrifuge tube. Samples were treated with 5 µl RNase A (20 mg/ml) (Invitrogen) for 45 min at 37°C and with 20 µl Proteinase K (20 mg/ml) (NEB) overnight at 45°C.

**Day 4**: An additional 5 µl Proteinase K was added and samples were incubated for another 2 h at 45°C. Beads were collected on the wall of the tube. DNA was extracted from the supernatant with Agencourt AMPure XP beads and resuspended in 20 µl 10 mM Tris-HCl at pH 8.0. The purified DNA was treated with 1 µl of 100 U/ µl Exonuclease III (NEB) in 90 µl 1× NEBuffer 1 for one h at 37°C. The reaction was ended by adding 2 µl 0.5 M EDTA and 2 µl 5 M NaCl, and subsequent incubation at 70°C for 20 min, followed by cooling to 4°C. The total volume was added up to 100 µl and transferred into a 130 µl Covaris miroTube with snap cap. DNA was sonicated on a Covaris S220 for 3 minutes using 5% duty factor, 200 cycles per burst and a peak incident power of 175. Ten µl of MyOne Streptavidin C1 magnetic beads (Invitrogen) were washed twice with 500 µl 1× Bind & Wash (B&W) buffer (5 mM Tris-HCl at pH 7.4, 0.5 mM EDTA, 1 M NaCl) and resuspended in 100 µl 2× B&W buffer. The sonicated DNA sample and

the C1 beads were mixed and incubated at RT for 30 min. The beads were washed once with 500 µl 1× B&W buffer with 0.1% Triton, once with 500 µl 10 mM Tris-HCl at pH 8.0, resuspended in a total volume of 100 µl of end repair mix (2 U of DNA Polymerase I, Large (Klenow) Fragment (NEB), 6 U of T4 DNA Polymerase (NEB), 20 U of T4 Polynucleotide Kinase (NEB) in 1× T4 DNA Ligase Buffer (NEB) with 0.4 mM of dNTPs) and incubated for 30 min at 20°C. The reaction was stopped by adding 1 µl of 0.5 M EDTA. The beads were once with 500 µl 1× B&W buffer with 0.1% Triton, once with 500 µl 10 mM Tris-HCl at pH 8.0, resuspended in 50 µl of A-tailing mix (15 U of Klenow Fragment (3′→5′ exo–) (NEB) in 50 µl 1× NEBuffer 2 with 0.2 mM dATP) and incubated for 30 min at 37°C. The beads were once with 500 µl 1× B&W buffer with 0.1% Triton, once with 500 µl 10 mM Tris-HCl at pH 8.0, and resuspended in 49 µl ligation mix (2000 U of T4 DNA ligase in 1x T4 DNA ligase buffer). One µl of 1.5 µM NEBnext adapter was added and the reaction mixture was incubated at 25°C for 60 min. After the addition of 1 µl of USER enzyme, the mixture was incubated for 15 min at 37°C. The beads were once with 500 µl 1× B&W buffer with 0.1% Triton, and once with 500 µl 10 mM Tris-HCl at pH 8.0. The library was amplified for 12 – 15 cycles using NEBnext Multiplex Oligos and KAPA HiFi Hotstart ReadyMix, and purified using Agencourt AMPure XP beads.

*H3K9me3 ChIP-seq*

Synchronized asexual parasite cultures were extracted in 0.15% saponin for 10 min on ice as previously described[9]. Subsequently, parasites were crosslinked for 10 min with 1% formaldehyde in PBS at 37°C, followed by quenching in 0.125 M glycine for 5 min at 37°C. Nuclear extractions were performed as previously described[9]. Stage IV-V gametocytes were purified and harvested using a percoll gradient as previously described[10]. Isolated gametocytes were incubated in lysis buffer (25 mM Tris-HCl, pH 8.0, 10 mM NaCl, 2 mM AEBSF, 1% Igepal CA-360 (Sigma Aldrich) for 10 min at RT and lysed by passing through a 26 G ½ inch needle fifteen times. Parasites were crosslinked by adding 1.25% formaldehyde, passing through a 26 G ½ inch needle ten times and incubating for 25 min at RT. To quench the crosslinking reaction, glycine was added to a final concentration of 150 mM, incubated for 15 min at RT followed by an incubation for 15 min at 4°C. Parasites were centrifuged for 5 min at 5,000 rpm at 4°C, washed with cold wash buffer (50 mM Tris-HCl, pH 8.0, 50 mM NaCl, 1 mM EDTA, 2 mM AEBSF, EDTA-free protease inhibitor cocktail (Roche)) and stored at -80°C.

For chromatin fragmentation, parasite nuclei were resuspended in shearing buffer (0.1% SDS, 1 mM EDTA, 10 mM Tris HCl pH 7.5, EDTA-free protease inhibitor cocktail (Roche), and phosphatase inhibitor cocktail (Roche) and sheared using the Covaris Ultra Sonicator (S220) for 6 min with the following settings; 5% duty cycle, 140 intensity peak incident power, 200 cycles per burst. To remove insoluble material, samples were centrifuged for 10 min at 14,000 rpm at 4°C. Fragmented chromatin was diluted 1:1 in ChIP dilution buffer (30 mM Tris-HCl pH 8, 3 mM EDTA, 0.1% SDS, 300 mM NaCl, 1.8% Triton X-100, EDTA-free protease inhibitor cocktail (Roche) and phosphatase inhibitor cocktail (Roche)). ChIP was performed as described previously[9] using 2 µg of anti-Histone H3K9me3 antibody (ab8898 (Abcam) for biological replicates #1 and 07-442 (Millipore) for biological replicates #2) or no antibody as a negative control. Libraries were prepared using the KAPA Library Preparation Kit (KAPA Biosystems)

and amplified for a total of 15 PCR cycles (15 cycles of [15 s at 98°C, 30 s at 55°C, 30 s at 62°C]). Libraries were sequenced on the Illumina NextSeq 500, generating 75 bp paired-end reads. Reads were mapped to the *P. falciparum* genome (PlasmoDB version 28.0) using bowtie2 v2.2.9[11]. To visualize read coverage in the IGV genome browser[12,13], read coverage was normalized per million mapped reads and the negative control values were subtracted from the IP sample values. To calculate H3K9me3 levels for each gene, read coverage per nucleotide was determined using BEDTools v2.26.0[14] and normalized per kilobase gene and per million mapped reads to obtain RPKM values. To further normalize the data for background noise, the negative control RPKM values were subtracted from the IP sample RPKM values. A RPKM of 40 was selected as the cutoff for positive H3K9me3 levels, based on the observation that more than 99.5% of genes that were not annotated as virulence genes or exported proteins were below that threshold. In addition, genes with differential H3K9me3 levels between trophozoites and gametocytes showed at least 2-fold difference in RPKM values.

*Hi-C data mapping, binning, and normalization*

We trimmed the 3' end of reads to a total read length of 40 bp and mapped the reads to the *P. falciparum* (PlasmoDB v9.0) and *P. vivax* (PlasmoDB v28) genomes using BWA, as previously described[15]. We binned both genomes into ten kilobase regions and assigned read pairs to the resulting $n$ bins, thereby obtaining for each Hi-C experiment a corresponding $n \times n$ raw contact count matrix $C$. In this matrix, each row and column corresponds to a 10-kb window, and each entry indicates the number of times the two regions have been observed in contact. Hi-C contact count matrices suffer from many technical and biological biases[16-18]. Accordingly, we normalize the raw contact count matrix $C$ using ICE[18] (http://github.com/hiclib/iced). This method decomposes the bias $\beta_{ij}$ associated with entry $C_{ij}$ of the contact count matrix into the product of two biases $\beta_i$ and $\beta_j$ associated with genomic regions $i$ and $j$. We denote the binned, normalized data as the "contact count matrices" $\hat{\mathbf{C}}$ throughout the text. Prior to normalization, we filter unmappable bins by first calculating the 50-bp mappability using GEM[19]. We then calculate the proportion of uniquely mappable bases across all regions that are within 400 bp of an MboI restriction site. We excluded 10-kb bins for which the average mappability within 400 bp of a MboI site was less than 25% from ICE normalization and subsequent analyses. For *P. falciparum*, 179 bins (7.7% of all bins) were discarded due to low mappability. Of those, 92 were within subtelomeric virulence clusters, 13 in internal virulence clusters, and 61 were outside the annotated virulence clusters but within the first or last 100 kb of the chromosome assembly (166 total). For *P. vivax*, 13 bins (0.6%) were discarded, 11 of which were subtelomeric (within 100 kb of the telomere). These unmappable bins are marked as grey in all heatmaps.

In addition, we calculated restriction enzyme-level data (as shown in Figure 4B and Supplementary Figure 13) by binning the *P. falciparum* genome by genomic regions between MboI sites and assigning contact counts as described. These matrices were not normalized. For comparisons between stages, we subsampled the mapped reads for each stage to the minimum number observed.

*Detecting errors in genome assembly*

The ability of Hi-C to identify genome rearrangements is the basis for its use in genome assembly. To illustrate the expected pattern produced by a translocation, we implemented a metric developed by Dudchenko and colleagues[20] to detect errors in genome assembly by Hi-C. We expect that most locations that are close together on the linear genome contact much more frequently than locations that are farther apart. To calculate a misassembly score, we first calculate a threshold value of the 95th percentile of all nonzero counts. All values in the Hi-C matrix are set to the minimum of either the normalized read count at that location or the 95th percentile. Next, a triangle 5 bins in height and with its apex at the diagonal of the matrix is scanned along the diagonal, and the metric $S_{observed}$ is equal to the sum of the values of all bins (after thresholding) within that triangle. The expected score $S_{expected}$ is the 95th percentile threshold multiplied by the number of bins inside the triangle. To avoid artifacts induced by mappability issues, we filtered out bins with <50% mappability by setting the value of counts from those bins to the threshold value. The final score is the ratio between $S_{observed}$ and $S_{expected}$.

*Identifying significant contacts and significant colocalization*

We modeled the effect of genomic distance on contact count probability with a spline using fit-hi-c to identify significant contacts between bins[15]. The results of this analysis are reported as a q-value assigned to each contact, where the q-value is defined as the minimum false discovery rate threshold at which a given discovery is deemed significant. Because of the possibility of differing statistical power between datasets, we report both the number of significant contacts above a given threshold, as well as the percent of significant contacts meeting a criterion (e.g. between *apiap2* gene loci and virulence clusters) out of all significant contacts. To correct ratios of contact counts for the effect of genomic distance, we calculated the expected number of contacts at that distance using fit-hi-c, and calculated the observed / expected statistic for each bin. The ratios reported in Supplementary Figure 4 are the ratios of those observed / expected statistics. We calculated significant colocalization of centromeres using previously described permutation tests, described in the text as the “Witten-Noble colocalization test”[21] and “Paulsen colocalization test”[22]. The main difference between the two tests is the use of intrachromosomal contacts; the Witten-Noble test is not designed to use them, while the Paulsen test is. For this reason, we use the Witten-Noble test to test the co-localization of centromeres (for which the important contacts are interchromosomal), and the Paulsen test to measure the colocalization of virulence clusters in the HP1 knockdown. We tested a number of sets of genes for colocalization among the eight stages investigated, so the p-values reported are corrected for multiple testing using the Benjamini-Hochberg FDR method.

**Resampling test for groups of genes**. To measure the significance of the overall change in contacts between groups of genes (e.g. *apiap2* genes and virulence clusters), we summed the number of contacts between bins overlapping the two groups of genes with a fit-hi-c q-value of less than 0.05 in the IDC stages (ring, trophozoite, schizont) and the transmission stages (stage II/III gametocytes, stage IV/V gametocytes, and sporozoites). We then compared those two sums using a one-sided sign test. In the case of the *apiap2* genes and virulence clusters, the alternative hypothesis was that it is possible that a random set of genes might have the same

difference in contacts between the IDC and transmission stages. To exclude that possibility, we conducted a resampling analysis, selecting groups of bins the same size as the *apiap2* genes (35 10-kb bins) and virulence clusters (191 10-kb bins) and repeating the sign test on the total number of fit-hi-c significant contacts. We then calculated a resampling p-value by calculating the number of times the sign test p-value was lower than the one we observed for the actual gene bins.

*Identifying differential contacts*

We developed ACCOST (Altered Chromatin COnformation STatistics) to estimate the statistical significance of differences in contact counts between samples without biological replicates. We modeled the observed counts in a given bin in each sample by a negative binomial distribution, similar to what DESeq[23], edgeR[24], and parametric rDiff[25] do for RNA-seq data, and HiC-DC (https://bitbucket.org/leslielab/hic-dc) does for single Hi-C experiments. We adapted DESeq to Hi-C data by using an explicit specific scaling factor for each bin count corresponding to bin-specific ICE biases. In addition, we estimated variance and dispersion of the negative binomial without replicates by assuming that most bins at a given genomic distance act similarly. Given two Hi-C contact matrices $\boldsymbol{C^A}$ and $\boldsymbol{C^B}$, with constant width bins indexed by $i$ and $j$, our goal is to determine which $(i, j)$ pairs have significantly different contact counts. An R package, diffHic[26], accomplishes this task in the case of multiple biological replicates, which are unavailable for most Hi-C experiments. Instead, we developed a method to estimate the variance in the contact counts using only a single replicate.

**Statistical model**. In any given sample, we model the raw read count $C_{ij}$ between bins $i$ and $j$ as a negative binomial random variable with size $r_{ij}$ and probability $p_{ij}$:

$$C_{ij} \sim NB(r_{ij}, p_{ij}) \, .$$

Remember that the mean and variance of $C_{ij}$ are then respectively

$$\mu_{ij} = \frac{r_{ij}(1 - p_{ij})}{p_{ij}} \, ,$$

$$\sigma_{ij}^2 = \frac{r_{ij}^2(1 - p_{ij})}{p_{ij}} \, .$$

To account for various bin-dependent biases and sample-specific overdispersion of the negative binomial, we model the mean and variance as

$$\mu_{ij} = \beta_i \beta_j q_{ij} \, ,$$

$$\sigma_{ij}^2 = \mu_{ij} + \beta_i^2 \beta_j^2 f(q_{ij}) \, ,$$

where $\beta = (\beta_1, \ldots, \beta_n)$ is a vector of bin- and sample-specific scaling factors, $f$ is a sample-specific smooth function, and $q_{ij}$ represents a measure of normalized interaction strength between bins $i$ and $j$. Note that the parameters of the NB distribution can be recovered from $\beta$, $f$ and $q$ following

$$r_{ij} = \frac{\mu_{ij}^2}{\sigma_{i,j}^2 - \mu_{i,j}} = \frac{q_{ij}}{f(q_{ij})},$$

$$p_{ij} = \frac{\mu_{ij}}{\sigma_{ij}^2} = \frac{q_{ij}}{q_{ij} + \beta_i \beta_j f(q_{ij})}.$$

**Estimation of $\beta$ and $f$.** Our model relates a normalized interaction strength $q_{ij}$ to the parameters of the NB distribution of the observed counts. It depends on the scaling factors $\beta$ and the smooth function $f$, which are both sample-specific. We estimate both of them on each sample independently, as follows. For $\beta$, we use Iterative Correction and Eigenvector decomposition (ICE) [18] to obtain an estimate $\hat{\beta} = (\hat{\beta}_1, \ldots, \hat{\beta}_n)$. To estimate $f$, we follow an approach similar to DESeq, adapted to the setting of Hi-C data, which generally lack replicates. To estimate $f$ without replicates, we assume that most bins at a given genomic distance from each other interact similarly, i.e. that $q_{i,j} = q_l$ is a function of the genomic distance $l = |i - j|$. For a given genomic distance $l$, denoting as $I(l)$ the set of $(i, j)$ pairs of genomic positions with $|i, j| = l$, we estimate the mean and variance of the normalized counts at distance $l$ respectively by:

$$\hat{q}_l = \frac{1}{|I(l)|} \sum_{(i,j) \in I(l)} \frac{C_{ij}}{\hat{\beta}_i \hat{\beta}_j},$$

and

$$\hat{v}_l = \frac{1}{|I(l)| - 1} \sum_{(i,j) \in I(l)} \left( \frac{C_{ij}}{\hat{\beta}_i \hat{\beta}_j} - \hat{q}_l \right)^2.$$

Similarly to DESeq, an unbiased estimator of the raw variance term $f(q_l)$ is

$$\hat{w}_l = \hat{v}_l - \frac{\hat{q}_l}{|I(l)|} \sum_{(i,j) \in I(l)} \frac{1}{\hat{\beta}_i \hat{\beta}_j}.$$

To further reduce the variance of the estimate, we use the assumption that $f$ is smooth to combine all $(\hat{q}_l, \hat{w}_l)$ pairs, for different genomic distances $l$, in a single nonparametric estimate $\hat{f}$ of $f$ using a local regression with generalized linear model of the gamma family, (using the locfit R package) so that

$$\hat{w}_l \simeq \hat{f}(\hat{q}_l).$$

**P-value computation**. Given two bins $i$ and $j$, and two samples $A$ and $B$ with respective counts $C_{ij}^A$ and $C_{ij}^B$ between both bins, we wish to test whether the normalized interaction strengths $q_{ij}^A$ and $q_{ij}^B$ are the same or not. We start by estimating the scaling factors and smooth functions $(\hat{\beta}_A, \hat{f}_A)$ and $(\hat{\beta}_B, \hat{f}_B)$ on each sample independently, as explained in the previous section. Under

the null hypothesis $\mathcal{H}_0 = \{q_{ij}^A = q_{ij}^B\}$, we can then estimate the common normalized interaction strength by

$$\hat{q}_{ij}^0 = \frac{1}{2}\left(\frac{C_{ij}^A}{\hat{\beta}_i^A \hat{\beta}_j^A} + \frac{C_{ij}^B}{\hat{\beta}_i^B \hat{\beta}_j^B}\right).$$

Combining this estimate with the estimated scaling factors $\hat{\beta}^k$ and smooth function $\hat{f}^k$ of each sample $k \in \{A, B\}$, we then model the count $C_{i,j}^k$ as a negative binomial random variable with parameters

$$\hat{r}_{ij}^k = \min\left(10^8, \frac{(\hat{q}_{ij}^0)^2}{\hat{f}^k(\hat{q}_{ij}^0)}\right),$$

$$\hat{p}_{ij}^A = \frac{\hat{q}_{ij}^0}{\hat{q}_{ij}^0 + \hat{\beta}_i \hat{\beta}_j \hat{f}^A(\hat{q}_{ij}^0)},$$

where the $10^8$ formulation ensures that the dispersion is never larger than $10^8$. We then follow the technique of DESeq to obtain a P-value by conditioning on the total count $C_{ij} = C_{ij}^A + C_{ij}^B$ and computing:

$$p_{ij} = \frac{\sum_{a+b=C_{ij}, p(a,b) \le p(C_{ij}^A, C_{ij}^B)} p(a,b)}{\sum_{a+b=C_{ij}} p(a,b)},$$

where

$$p(a,b) = P_{NB}(C_{ij}^A = a | \hat{r}_{ij}^A, \hat{p}_{ij}^A) P_{NB}(C_{ij}^B = b | \hat{r}_{ij}^B, \hat{p}_{ij}^B).$$

**Filtering**. Calculation of the p-value as written is expensive for large, high-resolution matrices, and may also potentially result in rounding error accumulation. Additionally, the high frequency of bins with only a few contact counts results in many calculations of p-values for differences in zero or a few contact counts, which will never reach statistical significance. For efficiency and to reduce the multiple testing burden, we only calculate p-values for intrachromosomal contacts for which the sum of the contact counts from the two samples is above the 80th percentile.

**Multiple testing**. We control the false discovery rate (FDR) using the Benjamini-Hochberg procedure. The Benjamini-Hochberg FDR is known to be conservative in the case of discrete test statistics[27]. Therefore, our corrected p-values are likely conservative.

**Availability**. ACCOST is implemented in Python and R and depends only on the standard libraries, NumPy and SciPy. ACCOST is available at:

http://noble.gs.washington.edu/proj/plasmo3d_sexualstages/.

*Inferring 3D models*

Our method for inferring the 3D structures is based on PASTIS[28]. Each chromosome is modeled as a series of *n* beads on string, each bead corresponding to a 10-kb genomic window. Contact counts $C_{ij}$ are modeled as negative binomial random variables, with two parameters: the mean $\mu_{ij}$ and the dispersion $r$. The mean $\mu_{ij}$ is parametrized as a decreasing function of the distance between bead $i$ and $j$: $\mu_{ij} = \gamma\beta_i\beta_j d_{ij}^{-3}$ (see ref. 28 for more information on the parametrization), where $\beta_i$ and $\beta_j$ are biases associated with bead $i$ and $j$, $\gamma$ is a scaling factor, and $d_{ij}$ is the Euclidean distance between bead $i$ and $j$. We can thus write the probability associated with each observation $p_{ij}$ :

$$p_{ij} = \frac{\Gamma(C_{ij} + \gamma\beta_i\beta_j r(d_{ij}^{-3}))}{\Gamma(C_{ij}+1)\Gamma(\gamma\beta_i\beta_j r(d_{ij}^{-3}))}\left(\frac{d_{ij}^{-3}}{r(d_{ij}^{-3}) + d_{ij}^{-3}}\right)^{C_{ij}}\left(\frac{r(d_{ij}^{-3})}{r(d_{ij}^{-3}) + d_{ij}^{-3}}\right)^{\gamma\beta_i\beta_j r(d_{ij}^{-3})}$$

The inference of the 3D structure can then be cast as maximizing the log-likelihood:

$$\max_{\gamma,\mathbf{X}} \quad \mathcal{L}(\mathbf{X},\gamma) = \sum_{i,j} \log(p_{ij})$$

We use the implementation from PASTIS[28] (http://cbio.ensmp.fr/pastis or https://github.com/hiclib/pastis) to perform the optimization. We initialize the variables randomly, and perform a local optimization using the L-BFGS algorithm, a quasi-Newton method.

**Stability of the inference**. The optimization problem used to infer 3D models of the genome is non-convex. Thus, the solution will depend on the initialization of the optimization, and we have no guarantees of obtaining the optimal solution. We thus perform 5,000 optimizations for each stage, and assess how much variance the resulting collection of structures exhibits. We compute for each structure of each stage a corresponding pairwise distance matrix, at a resolution of 100 kb. This computation yields for each structure a vector of 28,920 distances. We then perform a PCA analysis, and compare the intra-variance of these features among stages to the variance of the structures across stages. Because the feature matrix is very large, we perform a randomized PCA that uses an approximated singular value decomposition of the data to keep only the most significant singular vectors, thereby projecting the data to a lower dimensional space[29].

*Kernel canonical correlation analysis*

We use kernel canonical correlation analysis (KCCA)[30] to extract gene expression profiles that simultaneously capture the variance of gene expression profiles and are coherent with the 3D models. We previously performed a similar analysis to extract gene expression profiles coherent with asexual models of the genome[15]. Here, we review the underlying idea behind this analysis. Full details are presented in previous work[15].

Let $\mathcal{G}$ be the set of $k$ *P. falciparum* genes. We represent each gene by its log expression profile at $p$ time points $(e_1(g),\ldots,e_p(g))^{\mathrm{T}} \in \mathbb{R}^p$ and its position in 3D $x(g) \in \mathbb{R}^3$. We assume that the gene expression profiles are centered. We do not require the structures to be centered, because the measure of smoothness uses the Euclidean distances between each pair of genes.

We first look for a vector $v \in \mathbb{R}^p$ that captures variations between genes in expression. For that purpose, we assess the variance of the gene expression profiles once projected onto $v$:

$$V(v) = \frac{\sum_{g\in\mathcal{G}} \left(v^\top e(g)\right)^2}{\|v\|^2} .$$

The larger $V(v)$ is, the more $v$ captures the variance among the gene expression profiles. Note that finding a $v$ that maximizes the score $V(v)$ can be accomplished by extracting the first principle component of the gene expression matrix. We denote by $f_v$ the $k$-dimensional vector $f_v = (v^{\mathrm{T}} e(g), g \in \mathcal{G})$. The vector $f_v$ can be thought of as a vector of scores, one score per gene, corresponding to the projection of the gene expression profile onto $v$. It can then be shown that any profile of interest can be written as a linear combination of the gene expression profiles: $v(\upsilon) = \sum_{g\epsilon\mathcal{G}} \upsilon e(g)$, and thus the score $V$ can be expressed as

$$V(\nu) = \frac{\nu^T K^2 \nu}{\nu^T K \nu} ,$$

where $K$ is the $k \times k$ kernel matrix with entries $K_{i,j} = \sum_{l=1}^{p} e_l(i) e_l(j)$. Note that with these notations, we get $f_v = K\upsilon$.

Next, we carry out a similar decomposition for the 3D structure. In particular, we define a score $S(f)$ that measures how well a vector $f_{3D} \in \mathbb{R}^k$ of values per gene is smooth in 3D. We rely on a standard kernel method to quantify the smoothness of a function:

$$S(f_{3D}) = \frac{f_{3D}^T K_{3D}^{-1} f_{3D}}{\|f_{3D}\|^2} ,$$

where $K_{3D}$ is the Gaussian kernel matrix with entries

$$[K_{3D}]_{i,j} = \exp\left(\frac{-\|x(i) - x(j)\|^2}{2\sigma^2}\right) .$$

The smaller $S(f_{3D})$ is, the smoother the vector $f_{3D}$ in 3D is. Note that since $K_{3D}$ is invertible, any profile of scores $f_{3D}$ can be written as $K_{3D}\mu$.

Finally, to ensure that genes correlated with $v$ are also colocalized in 3D, we maximize the correlation between the scores $f_{3D}$ and $f_v$, while enforcing that $V(v)$ is large and $S(f_{3D})$ is small. This can be achieved by following the approach of ref. [30] and solving the following generalized eigenvalue problem:

$$\begin{pmatrix} 0 & K_v K_{3D} \\ K_{3D} K_v & 0 \end{pmatrix} \begin{pmatrix} \nu \\ \mu \end{pmatrix} = \rho \begin{pmatrix} (K_v + \delta I)^2 & 0 \\ 0 & (K_{3D} + \delta I)^2 \end{pmatrix} \begin{pmatrix} \nu \\ \mu \end{pmatrix} .$$

We extract the top-ranked generalized eigenvectors $(v, \mu)^T$, and thus recover the pair $(f_v, f_{3D})$ by $f_v = K_v v$ and $f_{3D} = K_{3D}\mu$.

Here, we focus on finding a gene expression profile $v$ that is representative of the variance of gene expression profiles and coherent not only with respect to the 3D model at one time point, but at multiple time points. This can be accomplished by considering the kernel $K_{3D}^*$ as the sum of the centered normalized Gaussian kernels of the different time points of interest. We divide the time points into three groups: the asexual stage (schizont, ring, trophozoite), the gametocyte stage (with both stage II/III gametocyte and stage IV/V gametocyte), and the sporozoite stage. Much can be learned from studying the properties of the expression scores $f_v$. We are interested in identifying whether gene clusters are among the genes with particularly high or low scores. We thus transform the scores to Z-scores by centering and scaling them to zero mean and unit standard deviation, and perform a t-test between the Z-scores associated with the genes in the gene cluster versus genes outside of the cluster. We consider enrichment of gene sets in the kCCA analysis significant if the P-value (after BH FDR correction) is less than 0.01 in both the gene expression and the structure component.

*Computational pipeline*

This section is intended to be used along with the computational methods provided above that describe the mathematical basis for the analyses performed, and focuses on specific scripts and parameters used for each step of analysis. Source code is available from Bitbucket (https://bitbucket.org/noblelab/plasmo-hic-2018/).

**1. Trimming and mapping**

Input: fastq files of reads

Output: pairs files

Source files: `generate_binned_midpoints.py` and `step2_GenerateFilterPairedAlignments.sh`

Sequenced reads ranged from 50 to 75 bp, and we trimmed them prior to mapping from the 30 end to a total length of 40 bp using cutadapt v1.9.dev2 with Python 2.7.3. Cutadapt parameters were: `-m 20 -u -N`, where `N` is the number of bases to remove to get 40 bp.

We mapped trimmed reads to the *P. falciparum* (PlasmoDB v9.0) and *P. vivax* (PlasmoDB v28) genomes using BWA v 0.7.3 with the command `bwa aln -t 8`.

We preprocessed the *P. falciparum* and *P. vivax* genomes with `generate_binned_midpoints.py` to generate 10-kb bins for each chromosome. We produced text les of paired mapped reads from the BWA output using `step2_GenerateFilterPairedAlignments.sh.`

**2. Mappability**

Input: *P. falciparum* and *P. vivax* genomes (.fa)

Output: mappability files

Source files: `process_genome_for_biases_combinedFrags-flankRE.py`

The *P. falciparum* and *P. vivax* genomes were pre-processed using GEMTools release 20100419 to calculate 50-bp mappability at each base. The commands used were:

```
gem-do-index --complement -i <genome fasta file> -o <prefix>
```

and

```
gem-mappability -I <prefix> -o mappability-50 -l 50 --output-line-width 500.
```

We calculated the mappability of each 10-kb region of the genome by averaging the mappability from -400 to +400 bp around restriction sites that fall within that 10-kb region, using `process_genome_for_biases_combinedFrags-flankRE.py`

**3. Binning, subsampling and normalization**

Input: pairs files

Output: raw and normalized contact count matrices

Source files: `generate_binned_midpoints.py`, `step2_get-contactCounts-atFixedWindowSize`, and `step2_get-subsampled-contactCounts-atFixedWindowSize`

We binned the *P. falciparum* and *P. vivax* genomes into 10-kb bins using generate_binned_midpoints.py and assigned read pairs to the bin to which they mapped using step2_get-contactCounts-atFixedWindowSize. This produced “raw” contact count matrices $\{C_{ij}\}$.

We performed all comparisons between time points (e.g. ACCOST or ratio plots) on data that was subsampled to the smaller read count using step2_get-subsampled-contactCounts-atFixedWindowSize.

We normalized Hi-C matrices using iterative correction and eigenvalue decomposition (ICE) as implemented in iced (https://github.com/NelleV/iced). This produced ICE biases for each 10-kb bin, as well as a normalized contact count matrix, where $C_{ij}^{norm} = \frac{C_{ij}^{raw}}{\beta_i \beta_j}$

### 4. Restriction site binning

Input: pairs files

Output: raw contact count matrices binned by restriction site

Source les: step1_findCutSites and step4_assign_cleanedPairs_toREsites

For certain analyses (e.g. the zoomed in view of the chr14 domain boundary) we used variable sized bins bordered by MboI restriction sites, which tend to be smaller than the 10-kb bins used otherwise. In this case, we determined MboI sites using step1_findCutSites and assigned read pairs to restriction fragments using step4_assign_cleanedPairs_toREsites.

### 5. Determining significant contacts

Input: raw contact count matrices, mappability les, ICE biases

Output: fit-hi-c p-value and q-value matrices

Source files: fit-hic-withInters-outputexp.py

We used a version of fit-hi-c that has been modified to output expected contacts to identify significant contacts between bins and calculate the expected contact count as a function of genomic distance. The parameters used were fit-hic-withInters-outputexp.py -l <name> -f <mappability file> -i <raw counts> -L 0 -U -1 -b 100 -m 1 -p 2 -r -l -t <ICE biases> --usebinning.

### 6. Significant co-localization

Input: normalized contact count matrices, list of gene loci

Output: single p-value

Source files: `contact_counts_to_matrix.py`, `colocalization_test.py` and `centromeres_all.tab`.

We used the Witten-Noble permutation test to test for significant co-localization of genes, as implemented in `colocalization_test.py`. Contact counts were reformatted prior to colocalization testing using `contact_counts_to_matrix.py`. Centromere locations are in `centromeres_all.tab`.

### 7. Differential contact count statistics

Input: two normalized contact count matrices, chromosome sizes file, mappability file

Output: p-value matrices and FDR corrected p-values (q-values)

Source files: ACCOST source can be found on github (https://github.com/cookkate/ACCOST), `get_FDR_from_lnpvals.py`

We calculating ACCOST p-values, representing the probability that a difference at least as extreme between normalized contact counts could be observed, using the following command:

```
differential_counts_directional.py <mappability file> <precalculated genomic distances> <precalculated genomic distances (reversed)> <raw counts A> <raw counts B> <ICE biases A> <ICE biases B> <output prefix> 1 0.25.
```

The precalculated genomic distances are optional and will be calculated if not provided, they correspond to the mapping from $i$ and $j$ bin values to genomic distances, and vice versa (for the reversed case). The last two parameters are a minimum p-value to target (1, not used) and a minimum mappability threshold (0.25).

FDR-corrected p-values were calculated using `get_FDR_from_lnpvals.py <pvalue matrix> <output filename> <chromosome sizes file>`.

### 8. Comparing matrices using ratios

Input: two normalized contact count matrices, fit-hi-c output

Output: ratio matrices

Source files: `get_corrected_ratio.py`

We corrected raw ratios for the effect of genomic distance by calculating the expected number of contact counts at a given distance $d = |i - j|$ using fit-hi-c (denoted below as $f(d)$). We calculated the ratio of observed normalized contact counts over the expected counts at each bin pair and report the ratio of those ratios, $R_{ij}$:

$$R_{ij} = \frac{C_{ij}^{A} + 1/f^{A}(|i-j|) + 1}{C_{ij}^{B} + 1/f^{B}(|i-j|) + 1}$$

The +1 construction accounts for the possibility of zeros in the denominator. We calculated corrected ratios using `get_corrected_ratio.py`.

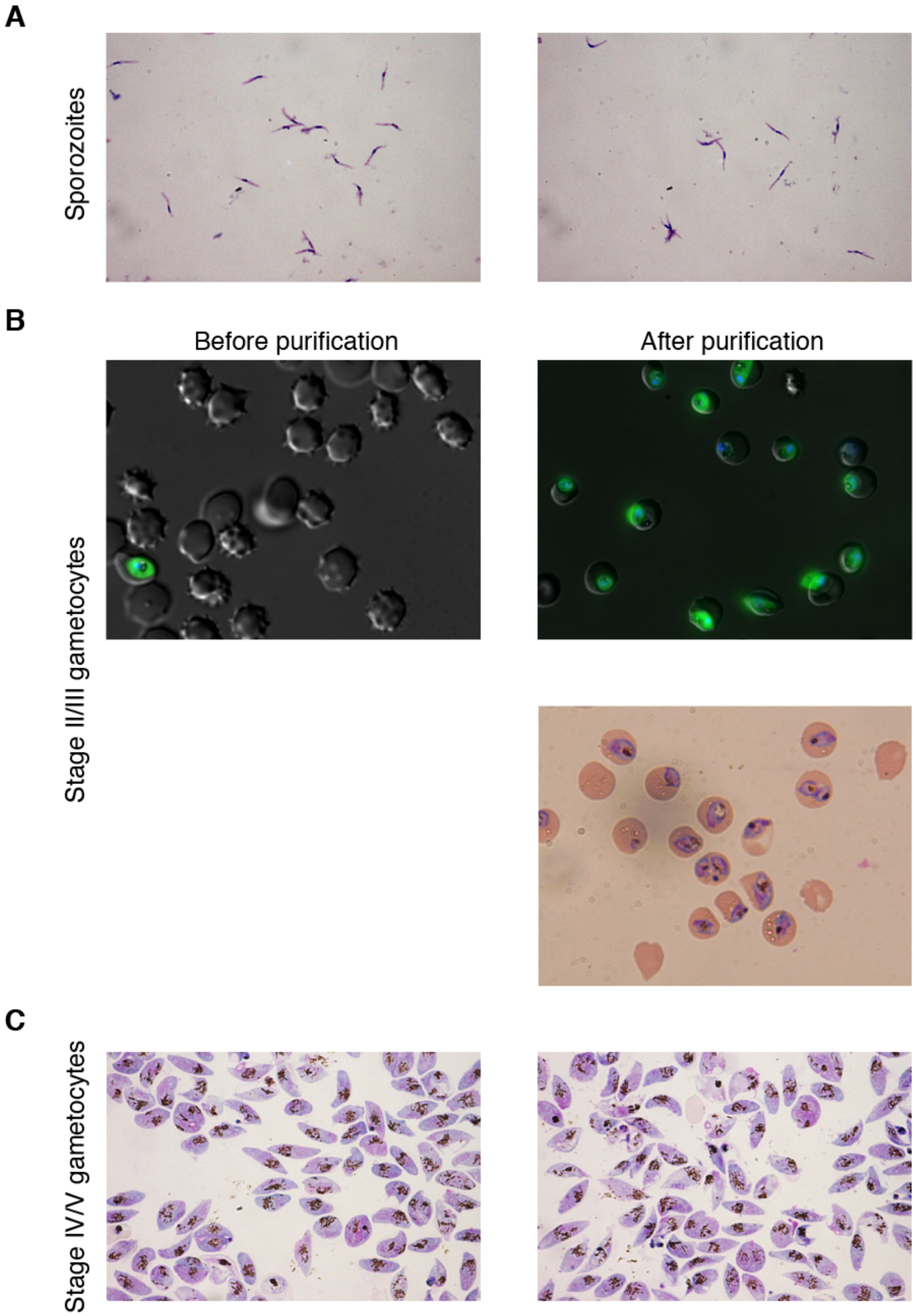


**Supplementary Figure 1: Microscopy images of the transmission stages analyzed in this study. (A)** Two representative images of Giemsa-stained *P. falciparum* salivary gland sporozoites. **(B)** GFP-expressing stage II/III gametocytes before (left) and after (right) purification visualized by fluorescent microscopy (top) or transmitted light microscopy of Giemsa-stained parasites (bottom). **(C)** Two representative images of Giemsa-stained stage IV/V gametocytes after percoll gradient and magnetic purification.

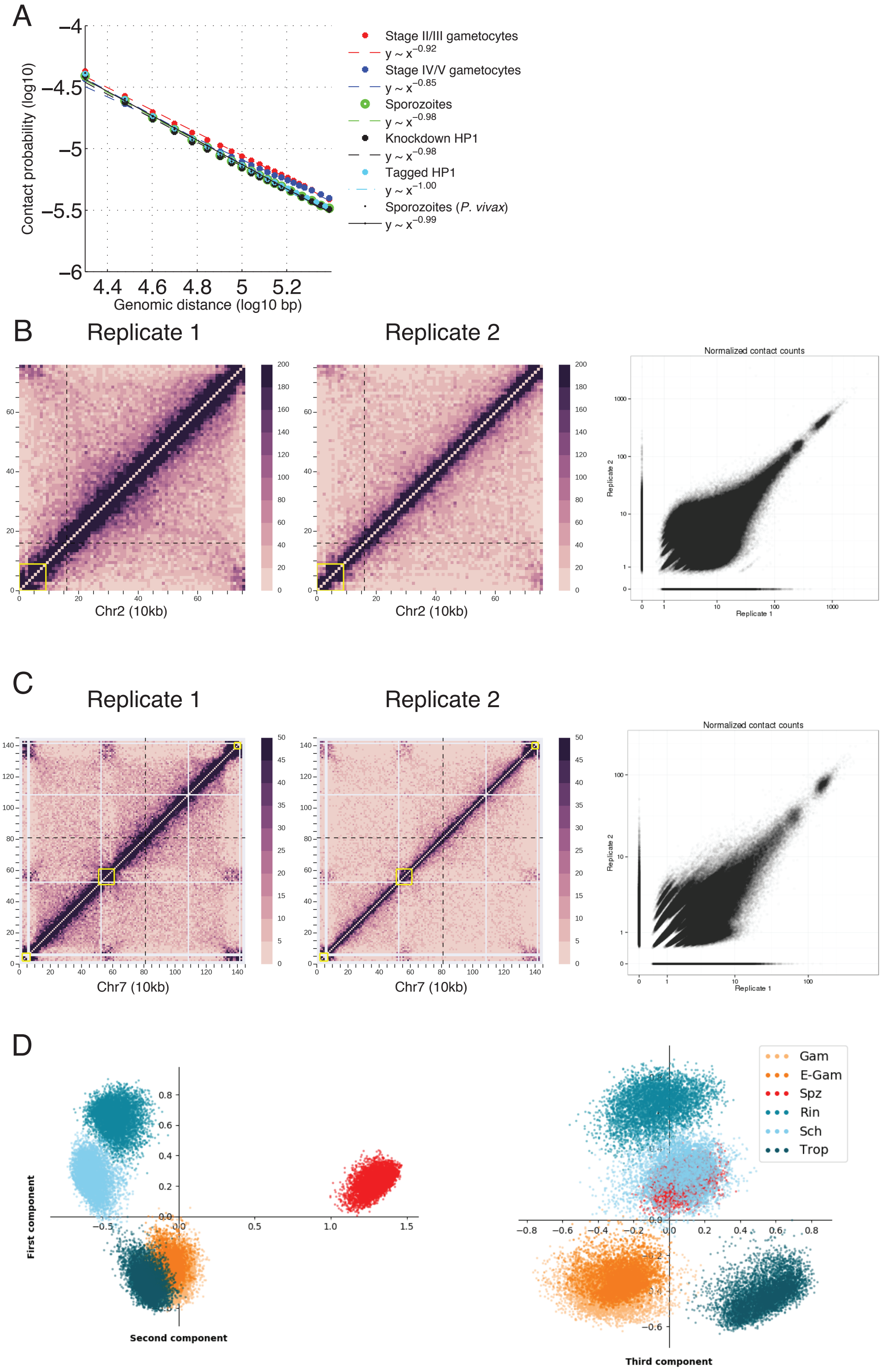
A
Contact probability (log10)
Genomic distance (log10 bp)
Stage II/III gametocytes
y ~ x−0.92
Stage IV/V gametocytes
y ~ x−0.85
Sporozoites
y ~ x−0.98
Knockdown HP1
y ~ x−0.98
Tagged HP1
y ~ x−1.00
Sporozoites (P. vivax)
y ~ x−0.99
B
Replicate 1
Replicate 2
Chr2 (10kb)
Normalized contact counts
Replicate 1
Replicate 2
C
Replicate 1
Replicate 2
Chr7 (10kb)
Normalized contact counts
D
First component
Second component
Third component
Gam
E-Gam
Spz
Rin
Sch
Trop

**Supplementary Figure 2: Quality measures of Hi-C libraries. (A)** Log-linear relationship between contact probability and genomic distance in all Hi-C libraries generated in this study. **(B)** ICE-normalized contact count matrices of chromosome 2 for two biological replicates of *P. vivax* sporozoites (left and middle) and ICE-normalized contact count scatter plot for these replicates (right). **(C)** ICE-normalized contact count matrices of chromosome 7 for two biological replicates of *P. falciparum* sporozoites (left and middle) and ICE-normalized contact count scatter plot for these replicates (right). **(D)** Principal component analysis on 5,000 3D genome structures per stage generated from varying initial starting points. The first and second components are plotted on the left, while the first and third components are plotted on the right.

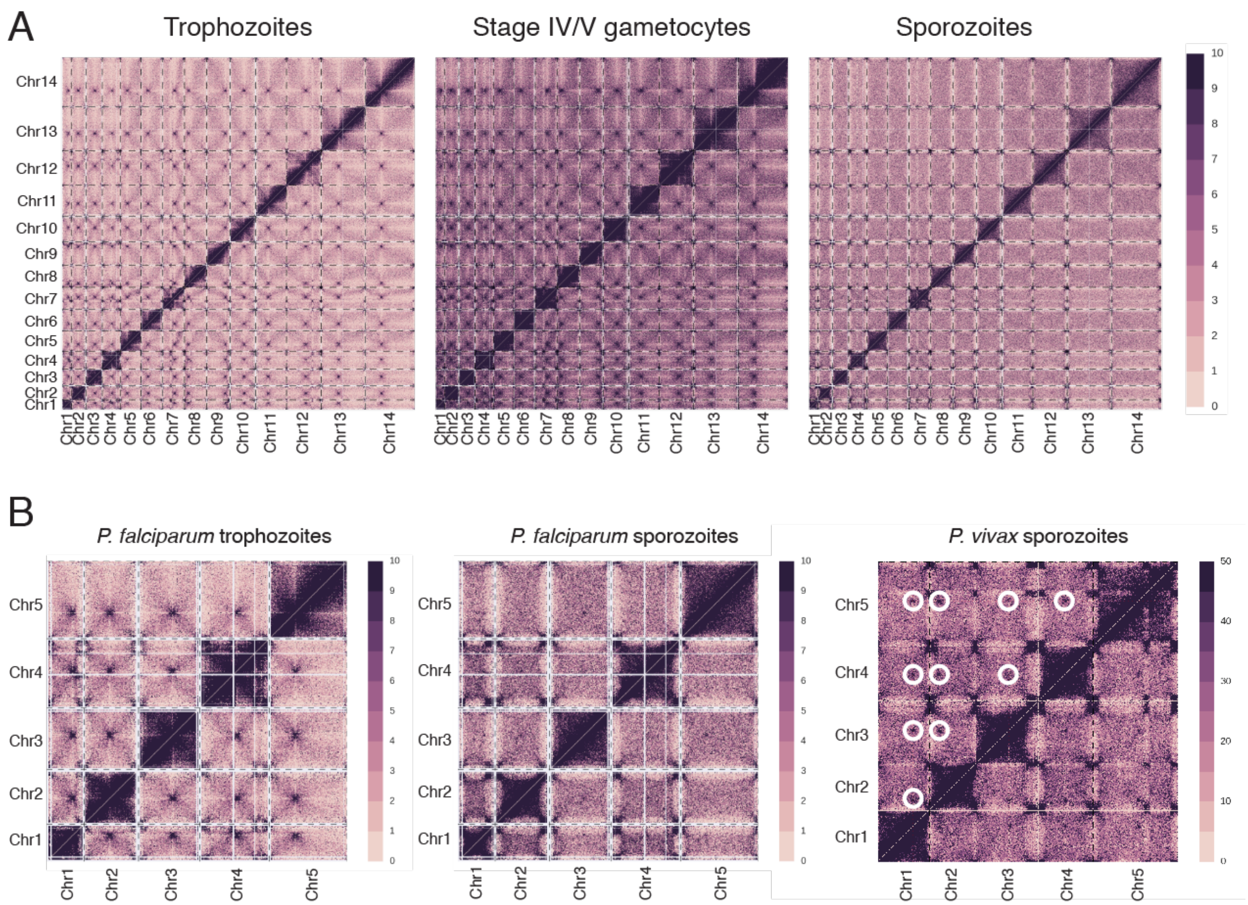


**Supplementary Figure 3: Differences in genome organization between *Plasmodium* species and life cycle stages. (A)** ICE-normalized interchromosomal contact count heatmaps at 10 kb resolution for *P. falciparum* trophozoites, stage IV/V gametocytes and sporozoites. Dashed lines indicate chromosome boundaries. **(B)** Interchromosomal contact count heatmaps of chromosomes 1-5 for *P. falciparum* trophozoites (left), showing strong co-localization of centromeres, *P. falciparum* sporozoites (center) with absent centromere co-localization, and *P. vivax* sporozoites (right), whose centromeres colocalize, although these interactions do not involve regions adjacent to the centromere. In all heatmaps, dashed black lines indicate chromosome boundaries, and white circles in the P. vivax sporozoite matrix are used to highlight inter-centromere contacts.

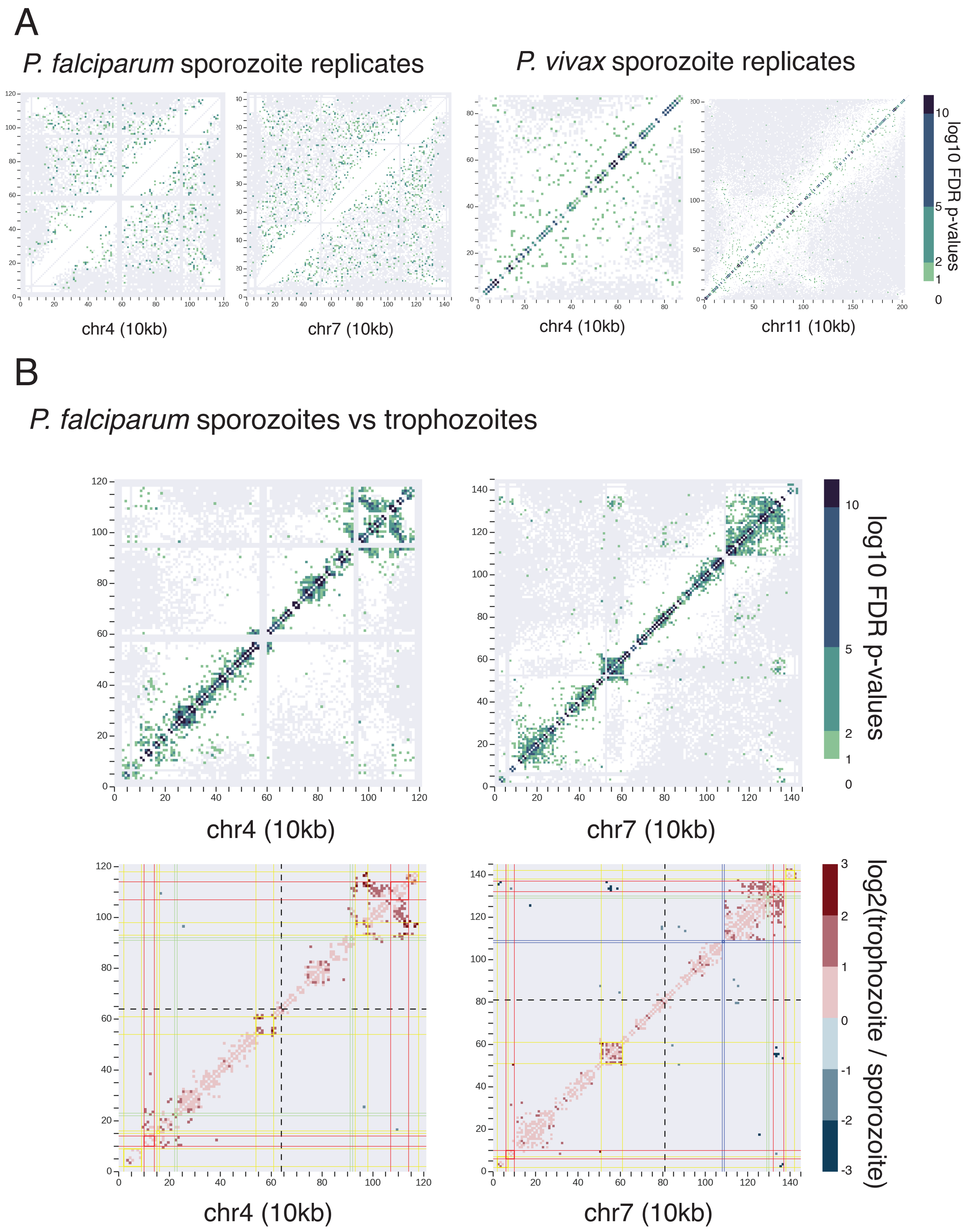

A
P. falciparum sporozoite replicates
P. vivax sporozoite replicates
chr4 (10kb)
chr7 (10kb)
chr4 (10kb)
chr11 (10kb)
log10 FDR p-values
B
P. falciparum sporozoites vs trophozoites
chr4 (10kb)
chr7 (10kb)
log10 FDR p-values
chr4 (10kb)
chr7 (10kb)
log2(trophozoite / sporozoite)

**Supplementary Figure 4: Similarities in genome organization between biological replicates as compared to differences in genome organization between different parasite stages. (A)** Heatmaps of differences in interactions between biological replicates of the sporozoite stage for *P. falciparum* (left panels for two different chromosomes) and *P. vivax* (right panels for two different chromosomes). The heatmaps are color-coded based on FDR p-values. No large-scale differences in interactions can be observed between biological replicates (compare: P. falciparum sporozoites vs trophozoites in panel B). Greyed out regions are bins that were filtered out for poor mappability, or loci for which the sum of the contact counts between the two samples was below the 80th percentile. **(B)** Heatmaps of all significantly changing interactions between trophozoites and sporozoites for chromosomes 4 (left) and 7 (right). The top panels show the FDR p-values. In the bottom panels, all 10 kb bins that differ significantly (at 1% FDR) in the number of interactions between the two stages are shown and are color-coded according to the direction of the change: loci with stronger interactions in trophozoites are indicated in red and in sporozoites in blue. Locations of genes of interest are bordered with color coded lines: virulence gene clusters are indicated in yellow, subtelomeric clusters of genes encoding exported proteins in red, ApiAP2 TF loci in green, and rDNA genes in blue. Centromeres are denoted by black dotted lines.

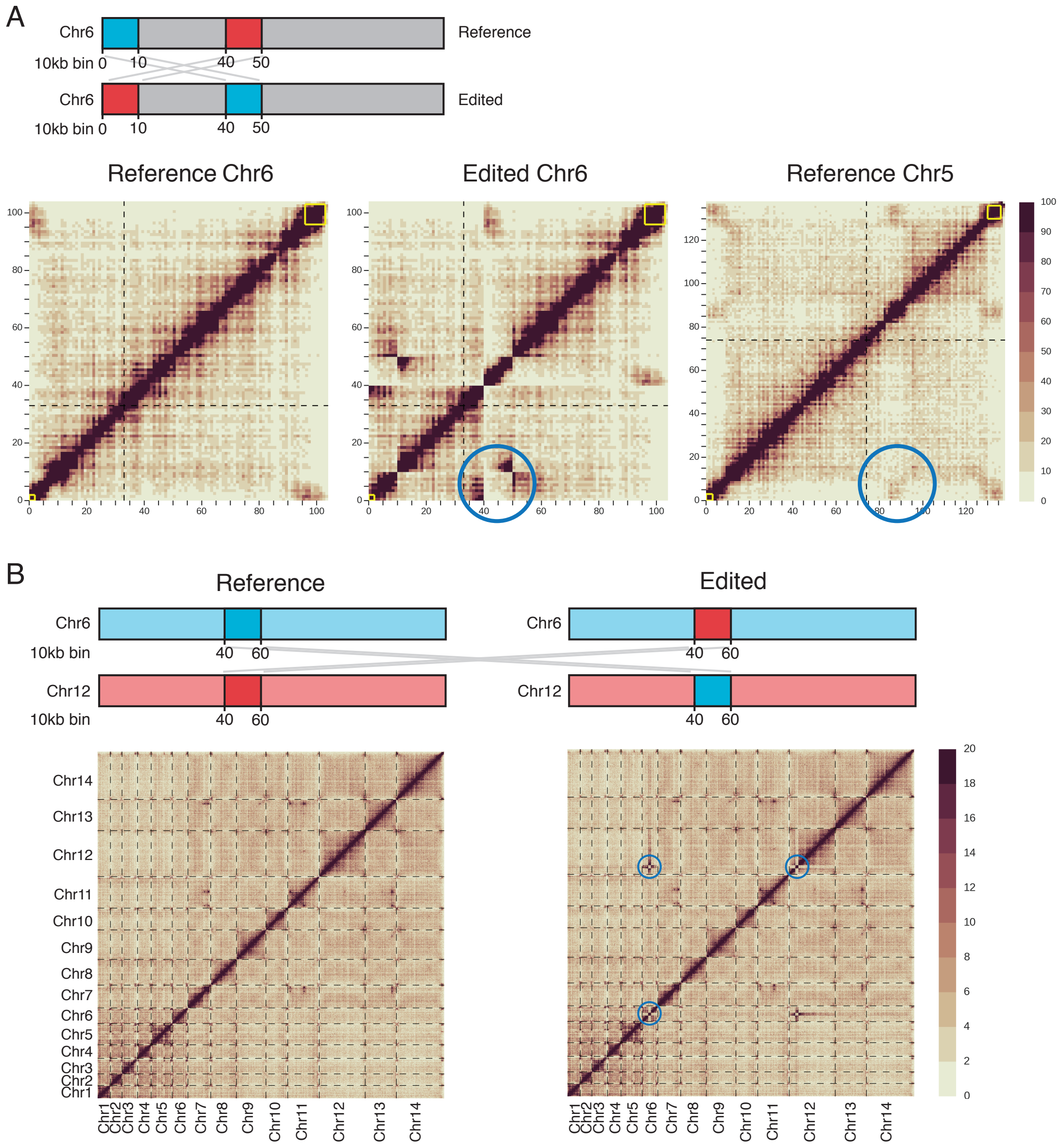


**Supplementary Figure 5: Introduced translocations in the *P. vivax* genome. (A)** A 100 kb intrachromosomal translocation in chromosome 6. A schematic representation of the introduced translocation is shown in the top. The bottom row shows three raw contact count heatmaps: observed contacts for chr6 (left), chr6 with introduced translocation (middle), and observed contacts for chr5 (right). **(B)** A 200 kb interchromosomal translocation between chromosome 6 and chromosome 12. A schematic representation of the introduced translocation is shown in the top. The bottom row shows the observed interchromosomal contacts (left) and the interchromosomal contact count heatmap after introduction of the translocation (right). These translocations produce aberrant signals in the contact count heatmaps (indicated with blue circles) that were not observed in any of the samples that were generated for this study. This supports our conclusion that the observed changes between life cycle stages are unlikely to be artifacts caused by genomic recombination.

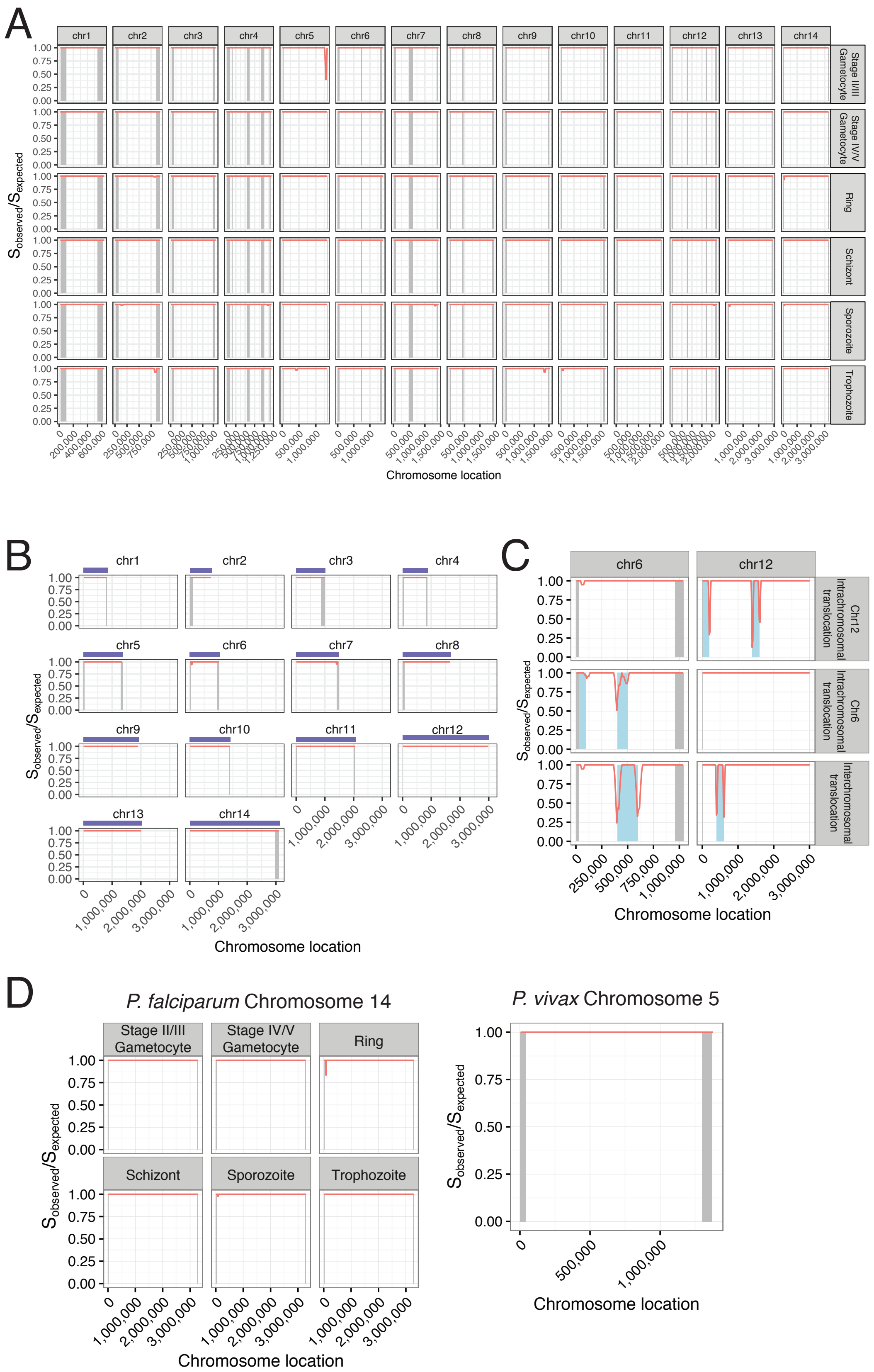
A
chr1
chr2
chr3
chr4
chr5
chr6
chr7
chr8
chr9
chr10
chr11
chr12
chr13
chr14
Stage II/III Gametocyte
Stage IV/V Gametocyte
Ring
Schizont
Sporozoite
Trophozoite
Sobserved/Sexpected
Chromosome location
B
Sobserved/Sexpected
Chromosome location
C
chr6
chr12
Chr12 Intrachromosomal translocation
Chr6 Intrachromosomal translocation
Interchromosomal translocation
Sobserved/Sexpected
Chromosome location
D
P. falciparum Chromosome 14
Stage II/III Gametocyte
Stage IV/V Gametocyte
Ring
Schizont
Sporozoite
Trophozoite
Sobserved/Sexpected
Chromosome location
P. vivax Chromosome 5
Sobserved/Sexpected
Chromosome location

**Supplementary Figure 6: Misassembly metric for *P. falciparum* and *P. vivax*.**
**(A)** The misassembly metric $S_{observed}/S_{expected}$ for all chromosomes of *P. falciparum* life cycle stages included in this study. Virulence clusters are shaded as grey bars. The region chr5:1,310,000-1,343,557 is likely deleted in the *P. falciparum* NF54$^{Pfs16}$ strain used to prepare stage II/III gametocytes, explaining the drop in the misassembly metric at the right telomere of chr5 in this sample. **(B)** The misassembly metric $S_{observed}/S_{expected}$ for all chromosomes in *P. vivax* sporozoites. Grey bars indicate the VIR gene clusters, and the purple bar at the top indicates the extent of the chromosome. **(C)** Misassembly metric on *P. vivax* data with simulated translocations. Clusters of VIR genes at the telomeres are shaded in grey, and the introduced translocations are shown in blue. Top row: Intrachromosomal translocation between 0-20,000 bp and 140,000-160,000 of chromosome 12. Middle row: Intrachromosomal translocation between 0-10,000 bp and 40,000-50,000 of chromosome 6. Bottom row: Interchromosomal translocation between 40,000-60,000 bp of chromosome 6 and 40,000-60,000 of chromosome 12. **(D)** The same data as (A) and (B), focusing on chromosome 14 of *P. falciparum* (left) and chromosome 5 of *P. vivax* (right).

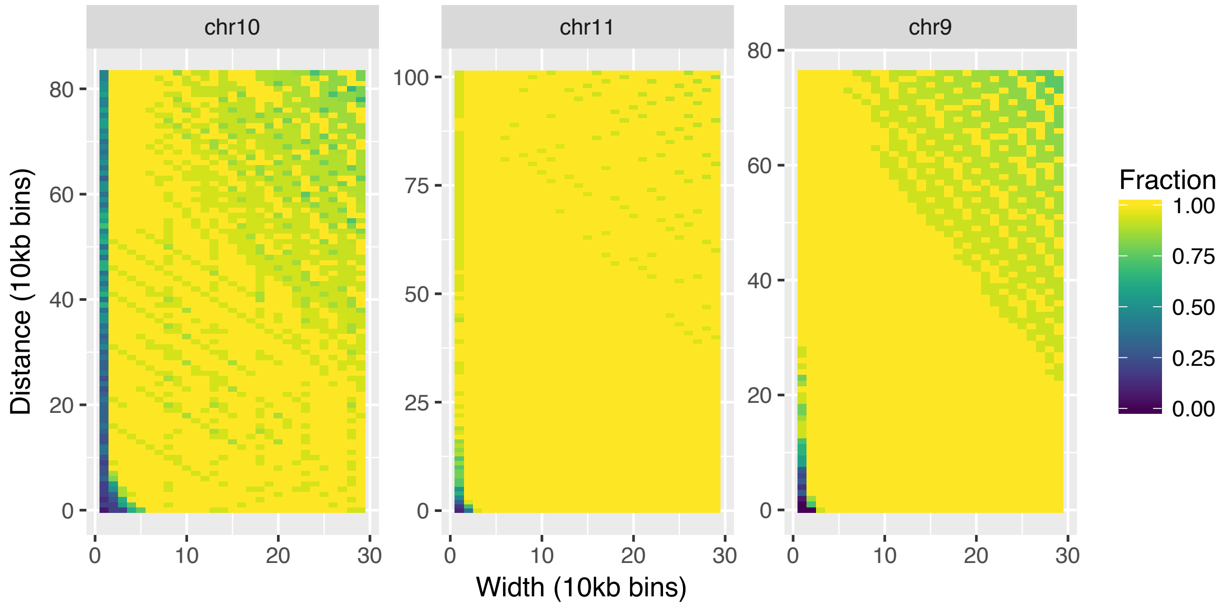


**Supplementary Figure 7: Detection of simulated translocations using the misassembly metric.** For each of three chromosomes in *Plasmodium falciparum* (chr9, chr10, and chr11), we generated translocations of widths down to a single 10kb bin, and with interchromosomal distances between the translocated regions down to zero bins (ie, right next to each other). Simulations were performed at locations across the chromosome ($n$ = 28,571 for chr9, $n$ = 35,647 for chr10, and $n$ = 52,873 for chr11). Using the misassembly metric $S_{observed}/S_{expected}$, we examined trophozoite Hi-C data across those chromosomes, and tallied the frequency of cases where the lowest value of the misassembly metric corresponded to the edge of one of the translocated regions. These frequencies were plotted as colors against the widths and distances tested.

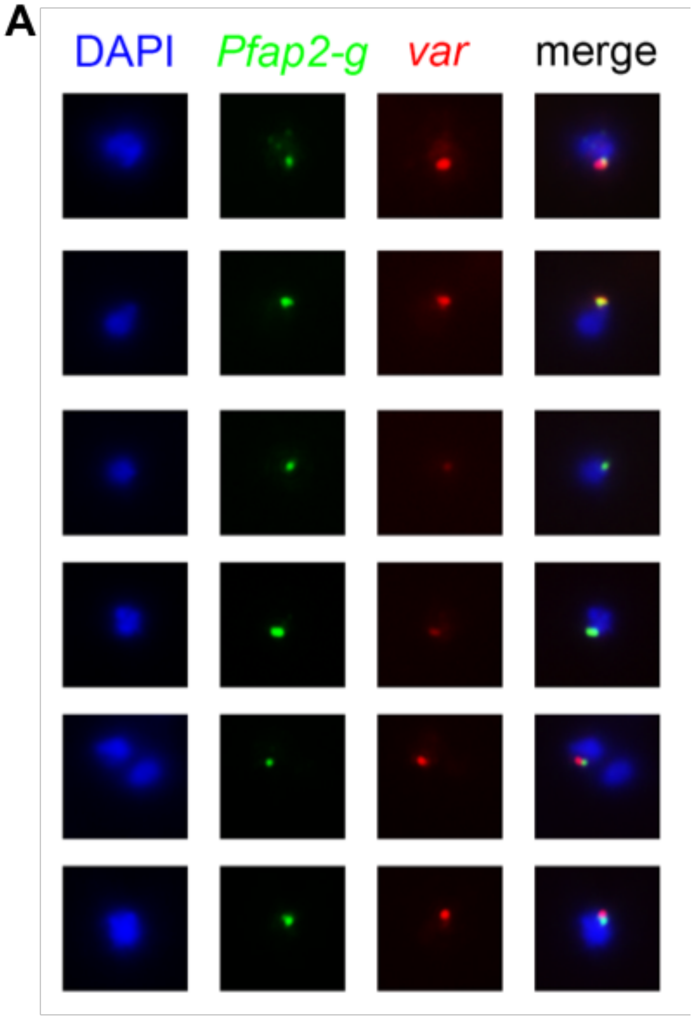


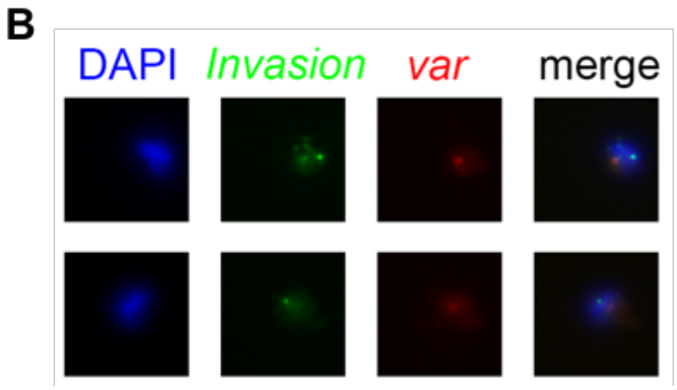


**Supplementary Figure 8: DNA-FISH experiments in ring stage parasites. (A)** Colocalization of *pfap2-g* and *var* gene PF3D7_0800300. Images are representative of visual inspection of >100 ring stage parasites. **(B)** Non-colocalizaton of invasion gene GLURP (chr10:1,399,195 – 1,402,896) and *var* gene PF3D7_0800300.

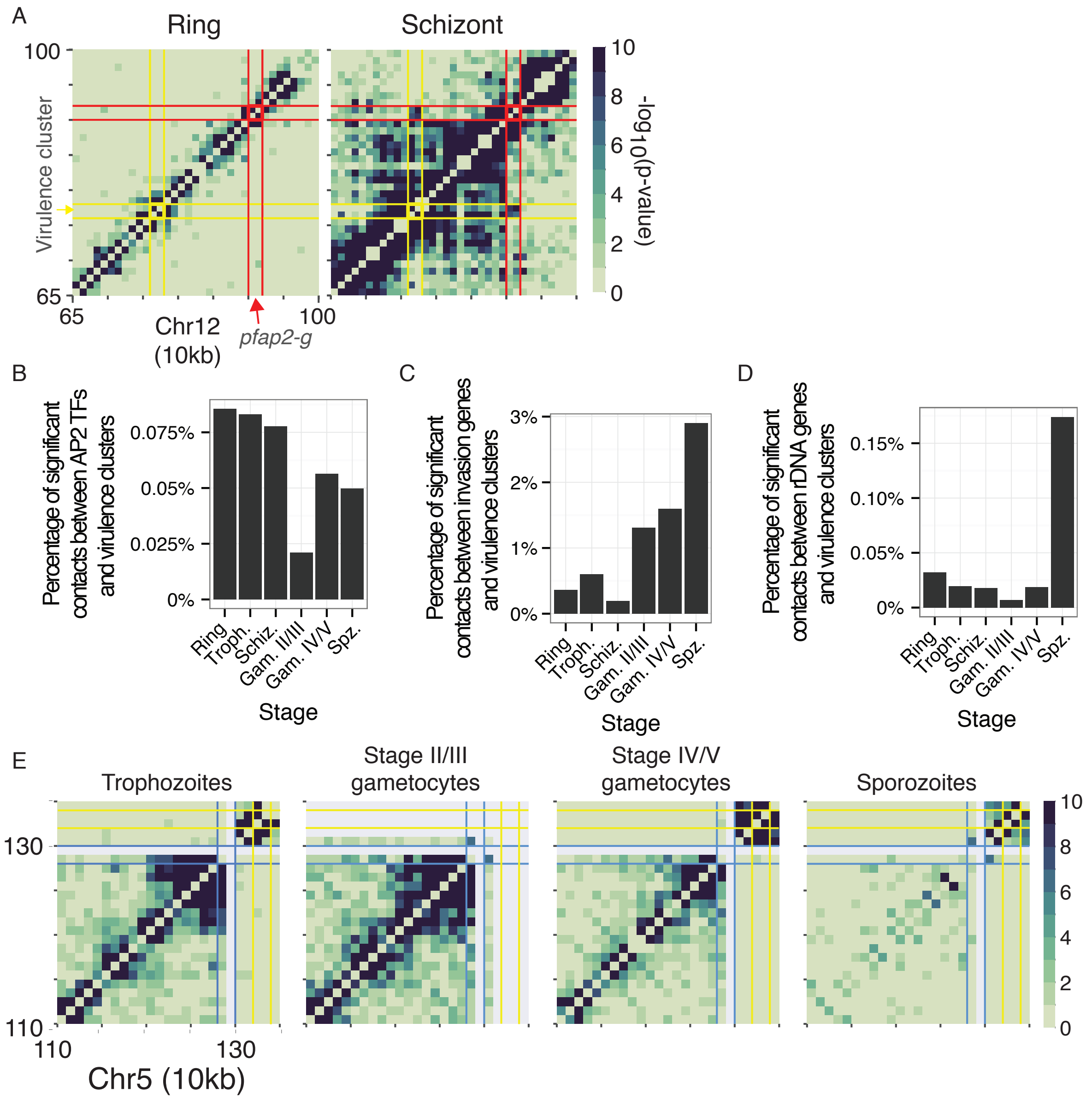


**Supplementary Figure 9: Interaction of ApiAP2 TF genes and invasion genes with the repressive center. (A)** Significant interactions between *pfap2-g* and the nearby internal *var* gene locus were also observed at the schizont stage (right panel) but not in the ring stage (left panel). A possible explanation for this observation is that the total number of significant interactions at the ring stage (n=16,705) is lower than at the trophozoite stage (n=25,457) and the schizont stage (n=160,176). The early gametocyte stage has a large number of significant interactions (n=209,345) and it is very unlikely that the absence of significant interactions between *pfap2-g* and *var* in this stage is caused by a lack of depth in the data. (**B-D)** Significant interactions between virulence genes and **(B)** *pfap2* genes, **(C)** invasion genes, and **(D)** rDNA genes are expressed as a percentage of all significant interactions within the genome. **(E)** Loss of domain formation around the rDNA locus on chr5 in *P. falciparum* sporozoites as compared to other life cycle stages. The borders of the rDNA locus are indicated by blue lines.

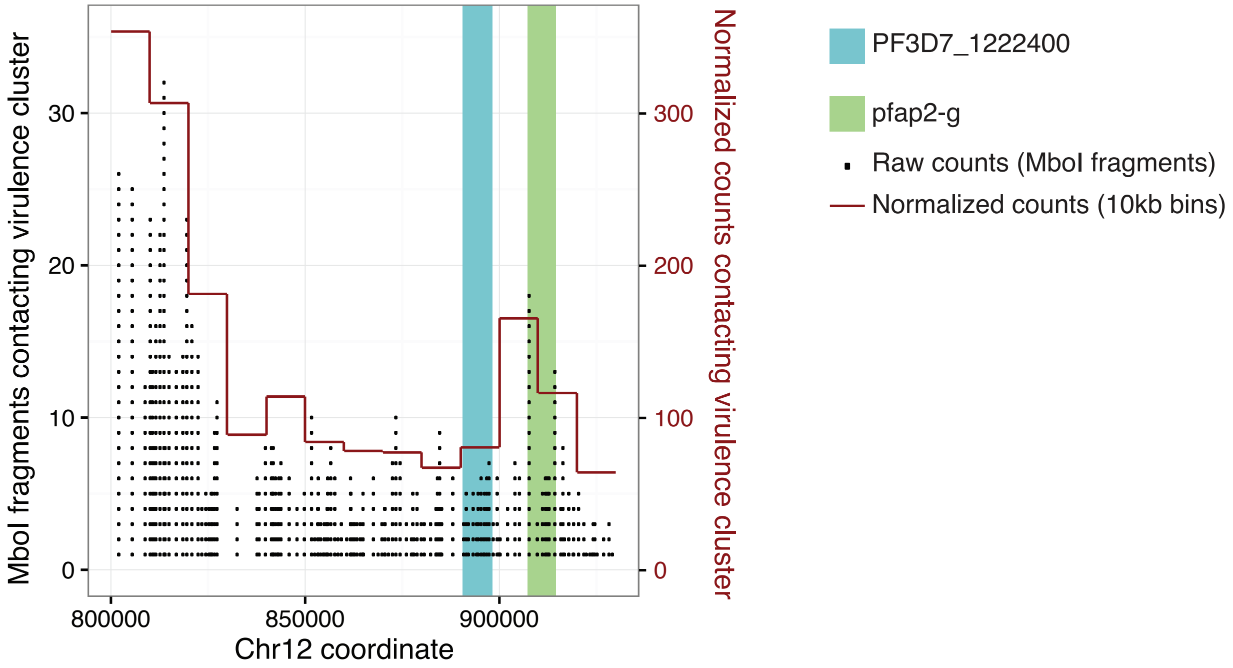


**Supplementary Figure 10: Restriction site resolution virtual 4C of chromosome 12 in trophozoites**. The chr12:764448-784830 virulence cluster was used as “bait” to collect reads mapping to individual MboI bins. Black dots represent individual reads and the left-hand axis represents the number of raw counts. The 10kb resolution ICE-normalized counts are plotted as red bars (each bar = one 10kb bin) with the right-hand axis representing the normalized contact count. The blue shaded area is the PF3D7_1222400 gene; the green area is *pfap2-g* (PF3D7_1222600).

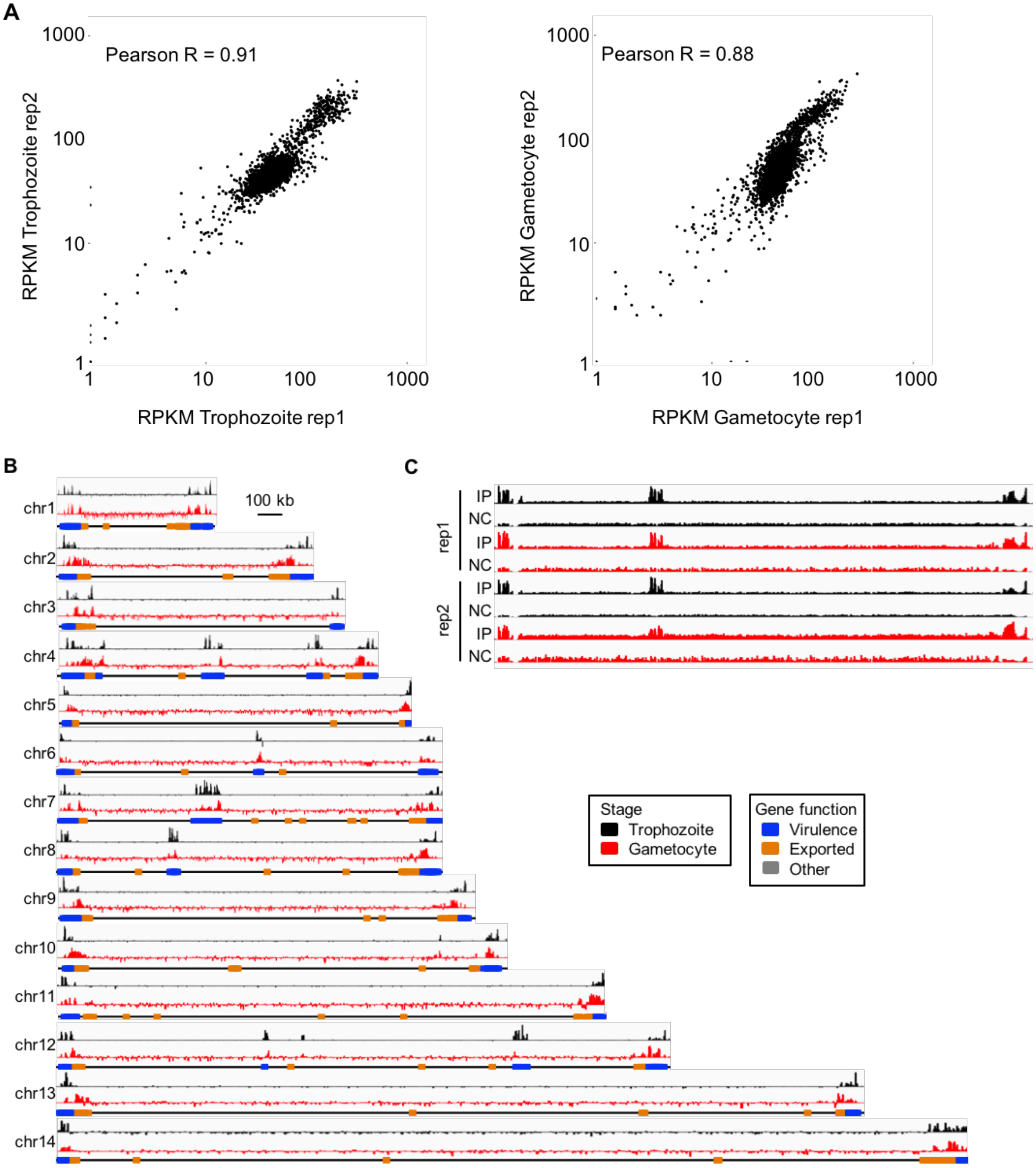


**Supplementary Figure 11: Quality measures of H3K9me3 ChIP-seq libraries. (A)** RPKM scatter plots for two biological replicates of *P. falciparum* late ring/early trophozoite stage (left) and gametocyte stage (right). **(B)** H3K9me3 ChIP-seq genome browser tracks of biological replicate 2 for trophozoite (top tracks in black) and stage IV/V gametocytes (bottom tracks in red). Results are similar to the tracks of biological replicate 1 presented in Figure 3A. **(C)** Raw read coverage of chromosome 8 for samples (IP) and negative controls (NC).

A

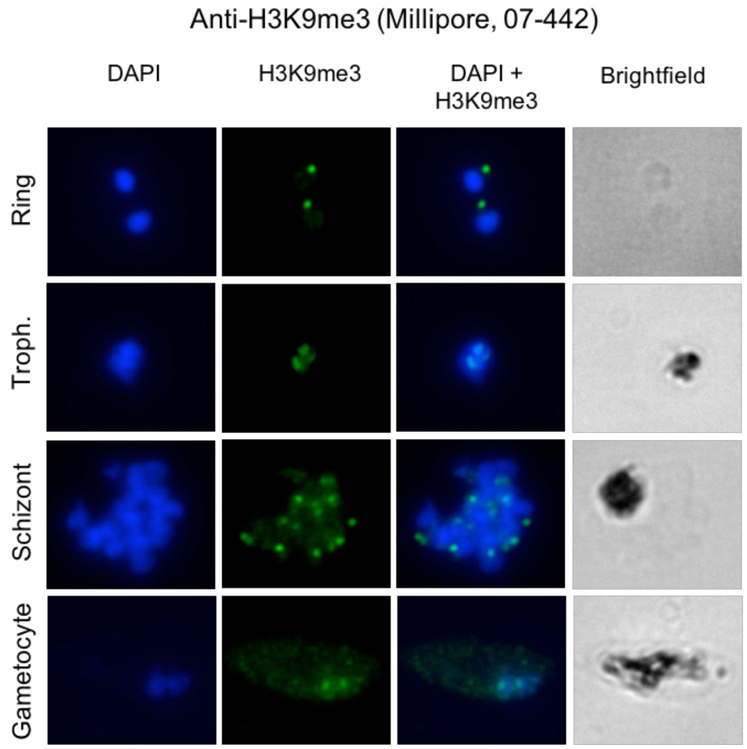


B

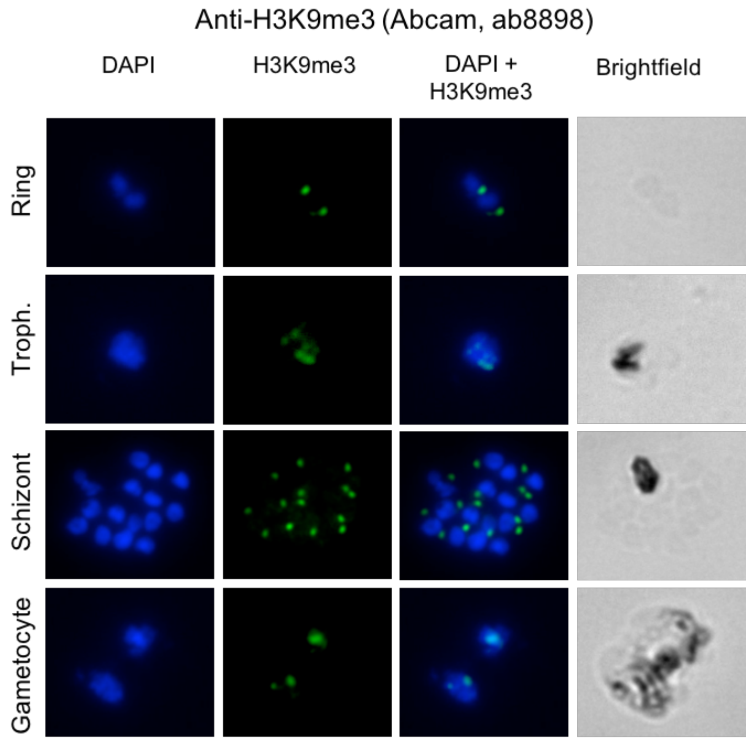


**Supplementary Figure 12: H3K9me3 immunofluorescence analysis in IDC and gametocyte stages.** The results of these experiments are highly comparable for anti-H3K9me3 antibodies Millipore 07-442 (A) and Abcam ab8898 (B).

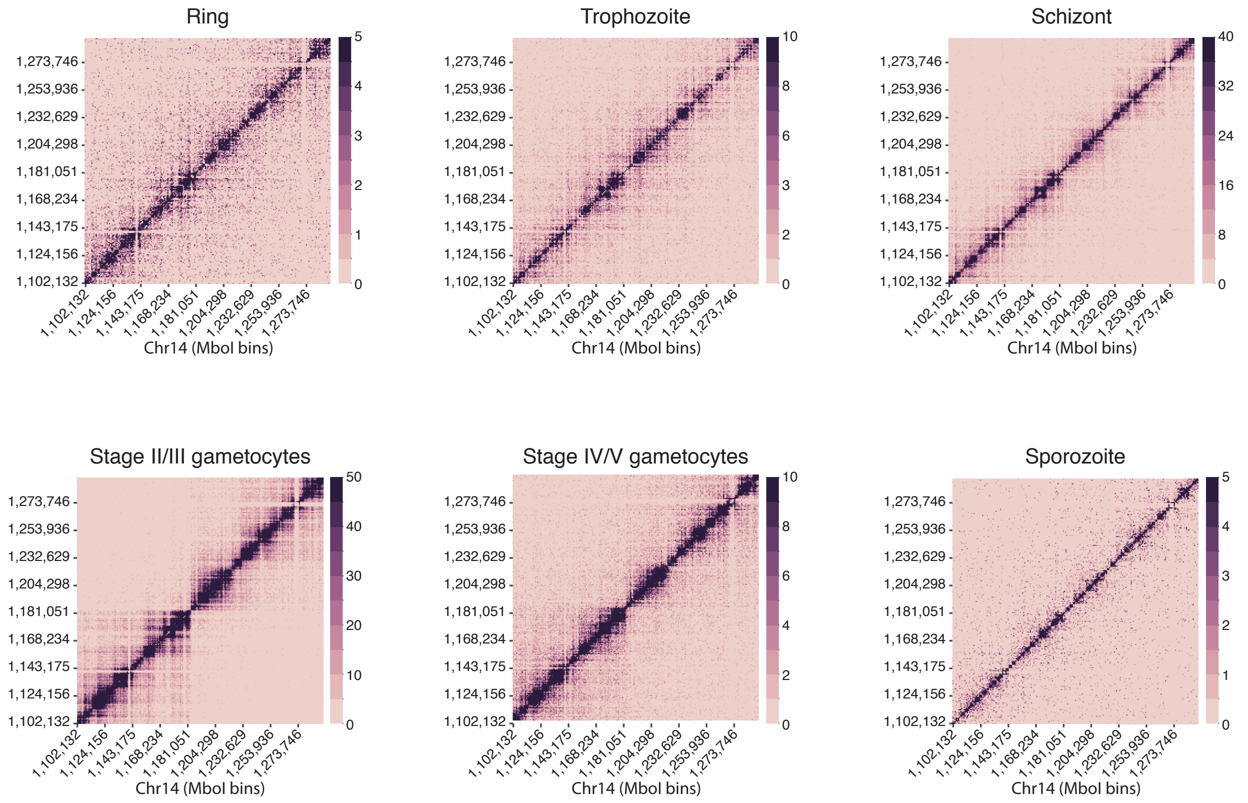


**Supplementary Figure 13: MboI restriction site resolution contact count heatmaps of the region surrounding the location of the domain boundary in chr14.** The domain boundary can be observed in both stage II/III and stage IV/V gametocytes, but is absent in other stages of the *P. falciparum* life cycle that were analyzed in this study.

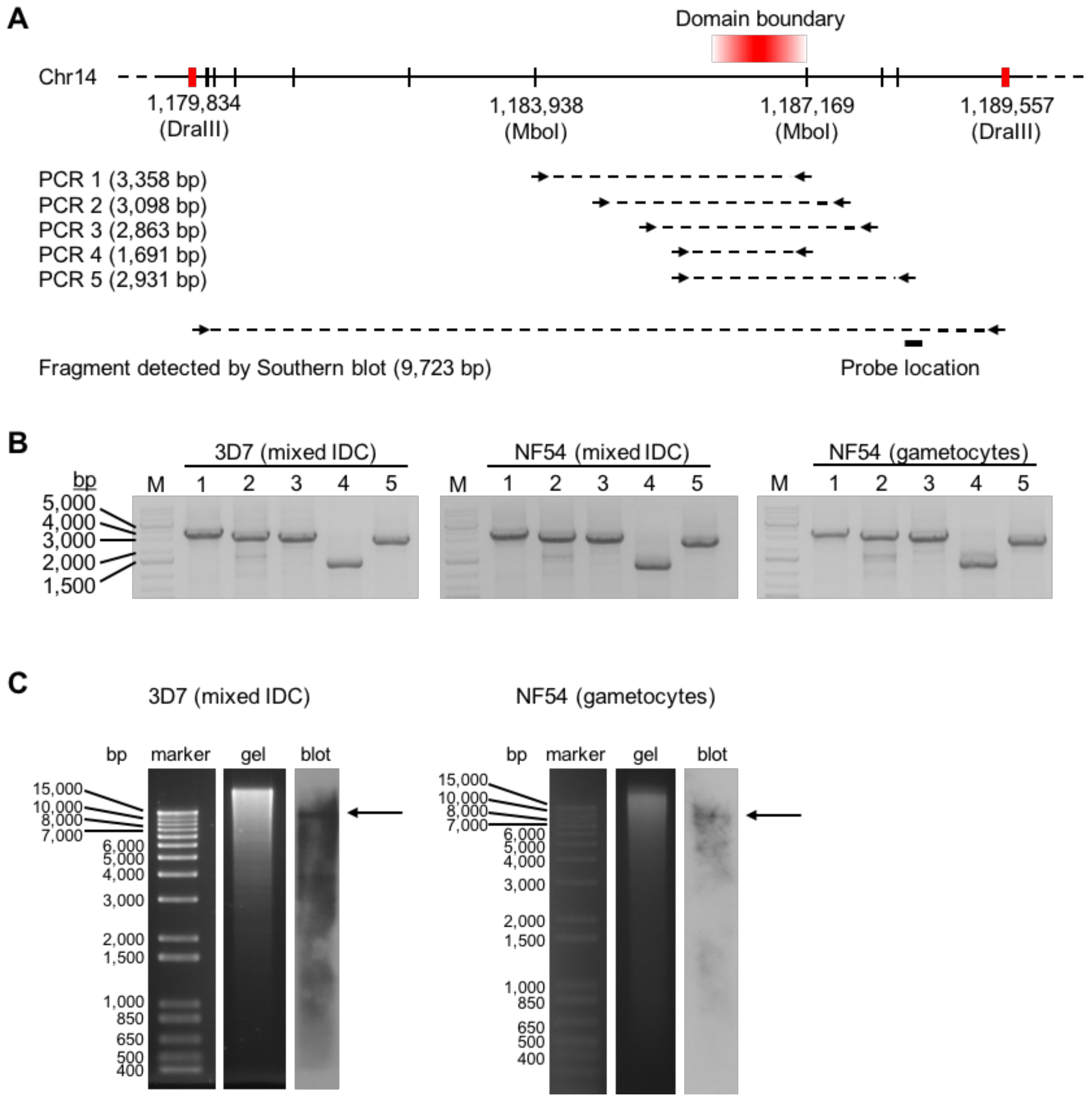


**Supplementary Figure 14: Confirmation of integrity of chromosome 14 around the domain boundary. (A)** Schematic overview of PCR and Southern blot experiments. **(B)** PCR amplifications of genomic DNA in the region chr14:1,183,922-1,188,519 isolated from *P. falciparum* strains 3D7 (DNA isolated from mixed IDC stages) and NF54 (DNA isolated from mixed IDC stages and gametocyte stage), which spans the region containing the domain boundary. Both strains show bands of the expected size, demonstrating that chromosome 14 is intact in both parasite strains. **(C)** Detection of the 9,723 bp fragment released by DraIII digestion in both mixed IDC stage parasites (strain 3D7) and gametocyte stage parasites (strain NF54), further confirming that chromosome 14 is intact in both stages of the parasite life cycle.

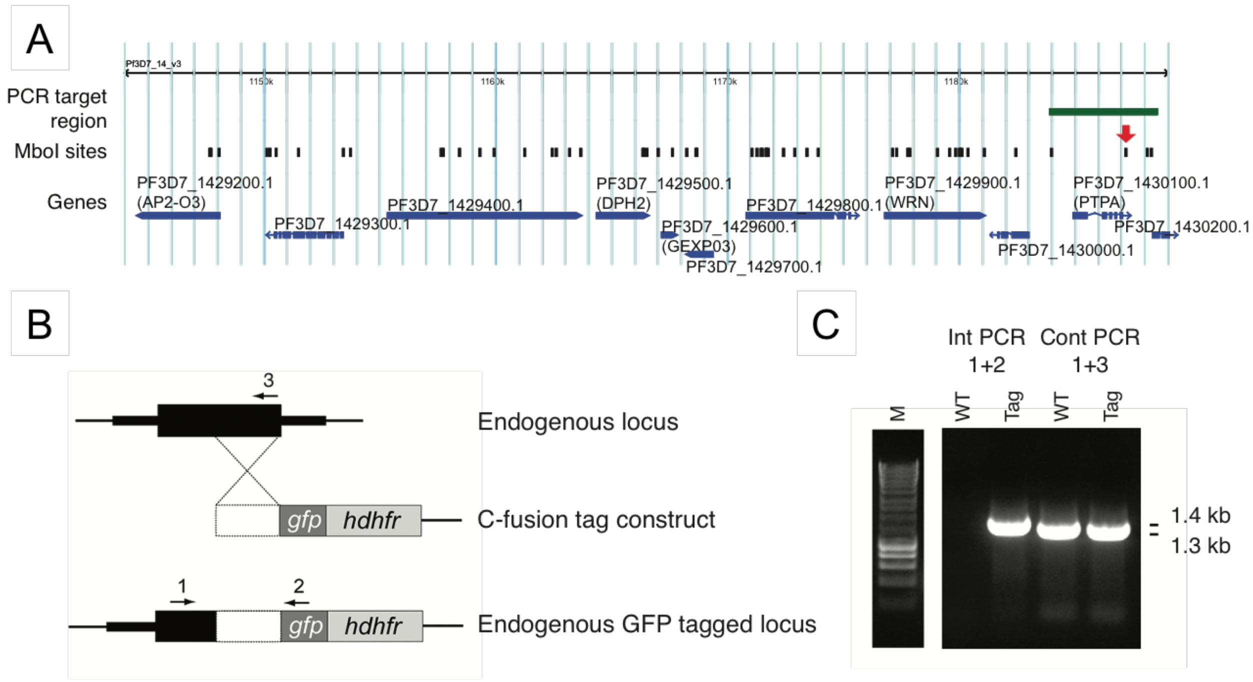


**Supplementary Figure 15: Investigating the role of *pfap2-o3* on chromosome 14. (A)** Gene model in the region around the location of the domain boundary on chromosome 14, close to the MboI restriction site at 1,187,169. *Pfap2-o3* (PF3D7_1429200) is located approximately 40 kb upstream of the domain boundary (red arrow). The region amplified by PCR as shown in panel A is indicated with a green bar. **(B)** Construction of transgenic *pbap2-o3-gfp P. berghei* parasites. Shown are schematic representations of the endogenous PBANKA_1015500 (*pbap2-o3*) locus (top), the GFP-tagging construct (middle) and the recombined PBANKA_1015500 locus following single cross-over recombination (bottom). Arrows 1 and 2 indicate PCR primers INT T219 and ol492 used to confirm successful integration in the PBANKA_1015500 locus following recombination. Arrows 1 and 3 indicate PCR primers INT T219 and T2192 used to control for the presence of the full-length template. **(C)** Results of the control PCRs on integration (left two lanes) and template integrity (right two lanes) of the PBANKA_1015500 locus in wild type (WT) and *pbap2-o3-gfp* (Tag) parasites.

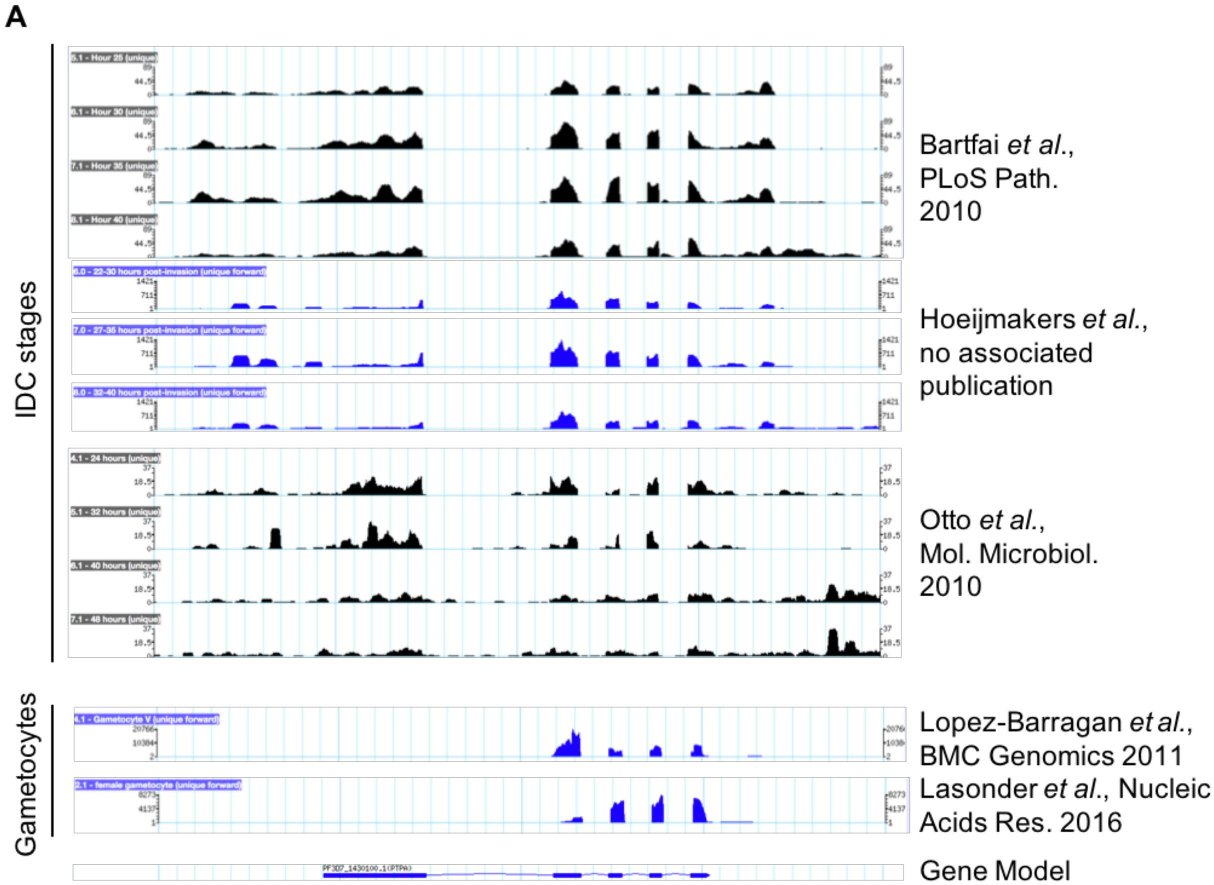


**B**

>PF3D7_1430100_INTRON1
GTACGTTTTTATTTATATATGAGTATATATTTGCATGTATATACACTTATGTTTTAA**TGCACATACACTT**
**AT**ACTTTAA**TGTACATACACTTAT**AGTTATGTATACTTGTATTGTAG**TGTACATACACTTAT**GTTTTAA**T**
**GTACATACACTTAT**ATTTTAA**TGTACATACACTTAT**ATTTTAATATATGTATACTTGTATTGTAGTGTAT
ATACACTTATATTTTAA**TGTACATACACTTAT**ATTTTAA**TGAACATACACTTAT**ATTTTAATGTATATAC
ACCTACATTTTAA**TGTACATACACTTAT**AATTATGTATACTTGTATTGTAGTGTATGTACACTTATGTTT
TAAAGTATGTATACTTATATTTTAATGTAAGTACACTTGTATTTTAGTGTATATACAATATGTTTTAATG
TATGTATAGTTGTATTTTATTGTATGTACACTTAAGCTTTAA**TGTACATACACTAAT**GCCTTAATGTACA
TGCACTTAAGCTTTAA**TGTACATACACTAAT**GCCTTAATGTATATGCATTTATGCTTTAA**TGTACATACA**
**CTTAT**ATTTTAATGTATGTGTGCTTGTGTTTTAA**TGTACATACACTTAT**GTTTTAATGTAAGTATATCTT
GGCTACATATATATATATATATATATATATTTTTTTTTTTTTTTCTTGTTTTTCAAAAATTTGCATGAACTG
TTCAG

**Supplementary Figure 16: Expression and sequence analysis of PF3D7_1430100 (PTPA). (A)** RNA-seq genome browser tracks from various publicly available data sets (obtained from PlasmoDB) showing the absence of exon 1 in the transcript expressed in gametocytes, while the variant expressed at the IDC stages contains all 5 exons. **(B)** The sequence of PF3D7_1430100 intron 1. A motif that is repeated a total of 12 times is highlighted in bold. Additional motifs can be found within the sequence.

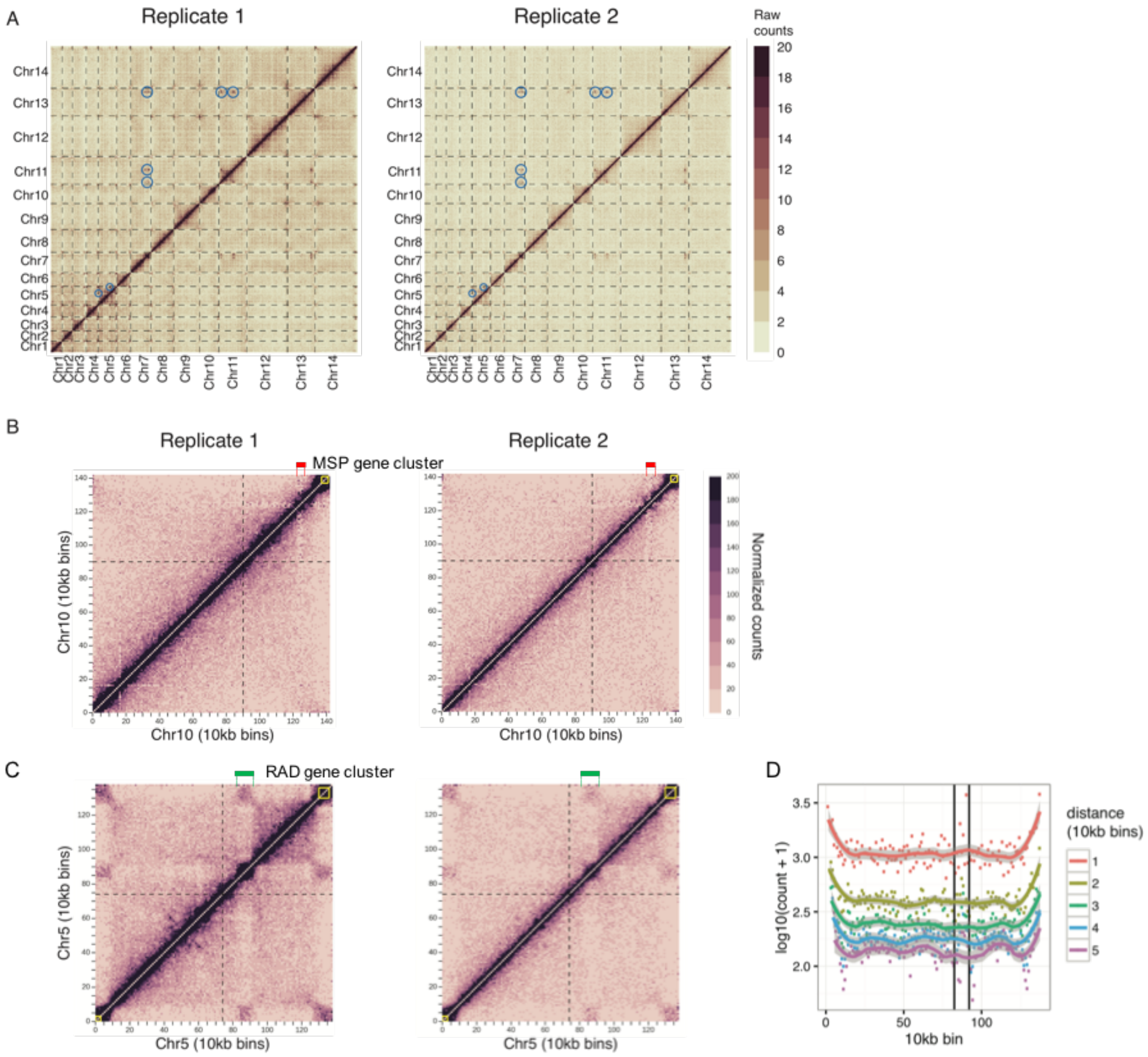


**Supplementary Figure 17: Characteristics of genome organization in *P. vivax* salivary gland sporozoites. (A)** Strong interchromosomal contacts. **(B)** *P. vivax* contains a cluster of merozoite surface proteins (MSP, i.e. invasion genes) on chromosome 10 (1,225,905 - 1,264,358, red bar) that does not interact with any other loci on chr10. **(C)** A subset of Pv-fam-e genes on *P. vivax* chromosome 5 (825,000-920,000, green bar) interacts with (sub)-telomeric regions of chr5 and other chromosomes, similar to the behavior of internal *var* gene clusters in *P. falciparum*. **(D)** Absence of a translocation event at the RAD gene locus on chr5 in *P. vivax*. Shown are the number of contacts between each 10 kb bin and its neighboring 10 kb bins up to 5 bins distance. A translocation event would show a breakpoint in the plot, instead of the smooth curves that are observed. Vertical lines indicate the borders of the RAD gene locus.

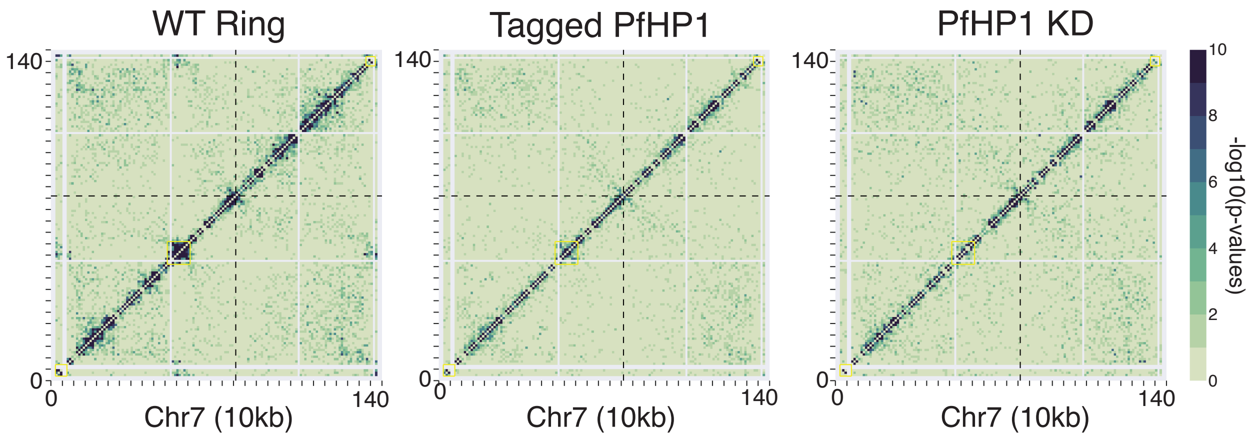


**Supplementary Figure 18: Tagging and depletion of PfHP1 result in a loss of *var* gene interactions.** Fit-hi-c P-value matrices of chromosome 7 are shown for the wild-type *P. falciparum* ring stage, the ring-stage PfHP1-GFP-DD strain cultured in the presence of Shield-1 (tagged PfHP1) and the ring-stage PfHP1-DD-GFP strain cultured in the absence of Shield-1 (knockdown [KD] PfHP1). P-values were calculated using subsampled data that contained the same number of interactions for each condition. Note the loss of interactions between the internal *var* gene cluster and the subtelomeric *var* genes in both the tagged and the knockdown PfHP1 strain.

**Supplementary Table 1**: Number of contact counts after mapping and filtering out interactions between loci that are less than 1 kb apart.

| Organism | Library | Contact counts |
|---|---|---|
| *P. falciparum* | Ring[1] | 8,980,937 |
| *P. falciparum* | Trophozoite[1] | 6,540,198 |
| *P. falciparum* | Schizont[1] | 31,093,712 |
| *P. falciparum* | Stage II/III gametocytes | 55,427,998 |
| *P. falciparum* | Stage IV/V gametocytes | 14,171,256 |
| *P. falciparum* | Sporozoites (combined) | 7,097,155 |
| *P. falciparum* | PfHP1-tagged strain | 37,362,915 |
| *P. falciparum* | PfHP1-knockdown strain | 7,115,059 |
| *P. vivax* | Sporozoites (combined) | 15,931,694 |

[1]Libraries were generated as part of a previously published study (Ay *et al*., Genome Research, 2014).

**Supplementary Table 2**: Interchromosomal contact probability (ICP) and percentage of long-range contacts (PLRC) values for each Hi-C library generated in this study.

| Organism | Library | ICP[1] | PLRC[2] |
|---|---|---|---|
| *P. falciparum* | Stage II/III gametocytes | 0.45 | 32.39% |
| *P. falciparum* | Stage IV/V gametocytes | 0.87 | 7.47% |
| *P. falciparum* | Sporozoites (replicate 1) | 1.44 | 5.05% |
| *P. falciparum* | Sporozoites (replicate 2) | 1.45 | 5.11% |
| *P. falciparum* | Sporozoites (combined) | 1.44 | 5.07% |
| *P. falciparum* | PfHP1-tagged strain | 1.64 | 10.63% |
| *P. falciparum* | PfHP1-knockdown strain | 1.64 | 3.08% |
| *P. vivax* | Sporozoites (replicate 1) | 1.75 | 5.62% |
| *P. vivax* | Sporozoites (replicate 2) | 1.66 | 5.24% |
| *P. vivax* | Sporozoites (combined) | 1.72 | 5.47% |
| ***P. falciparum*[3]** | **Trophozoite (not cross-linked)** | **7.82** | n.a. |

[1] ICP (inter-chromosomal contact probability index) is defined as (the number of inter-chromosomal interactions) / (the number of intra-chromosomal interactions above 1 kb distance).

[2] Percentage of long-range contacts is calculated as (the number of interchromosomal contacts + the number of intrachromosomal contacts over 20 kb distance) / the number of reads mapped to the *Plasmodium* genome.

[3] Control library generated without the formaldehyde cross-linking step of the Hi-C protocol from Ay *et al.*, Genome Research, 2014, included here for comparison.

**Supplementary Table 3**: The sum of Hi-C contacts for significant interactions (5% FDR) between 10 kb bins containing *pfap2* genes and 10 kb bins containing virulence genes.

| Gene | Location | R | T | S | EG | LG | SPZ | Notes |
|---|---|---|---|---|---|---|---|---|
| PF3D7_0404100 | chr4:224,779-231,733 | 7 | 11 | 6 | - | - | 7 | n.a. |
| PF3D7_0420300[1] | chr4:917,990-928,411 | 33 | 114 | 55 | 9 | 55 | 12 | n.a. |
| PF3D7_0611200 | chr6:467,481-468,642 | - | - | 5 | - | - | - | n.a. |
| PF3D7_0730300 | chr7:1,297,459-1,301,454 | - | - | 15 | - | - | 15 | *pfap2-l* |
| PF3D7_0802100 | chr8:151,808-159,668 | 9 | - | - | - | - | 4 | n.a. |
| PF3D7_0934400 | chr9:1,349,790-1,350,392 | 13 | - | - | - | - | - | n.a. |
| PF3D7_1139300 | chr11:1,556,744-1,565,045 | - | - | - | - | - | 6 | n.a. |
| PF3D7_1222400 | chr12:890,581-898,257 | - | 58 | 56 | - | - | - | n.a. |
| PF3D7_1222600 | chr12:907,203-914,501 | - | 215 | 23 | - | 87 | - | *pfap2-g* |

R, ring; T, trophozoite; S, schizont; EG, early (stage II/III) gametocytes; LG, late (stage IV/V) gametocytes; SPZ, sporozoites; n.a., not applicable.

[1]Interactions between PF3D7_0420300 and the nearby internal virulence gene cluster (in the adjacent 10 kb bin) were not included in this table to exclude any interactions caused by physical constraints.

**Supplementary Table 4**: Loci involved in long-range interactions in *P. vivax* sporozoites.

| Chr | locus (kb) | start | end | gene | description | Pf homolog |
|---|---|---|---|---|---|---|
| 3 | 185 | 184,691 | 187,491 | PVX_000945 | Apical sushi | PF3D7_0405900 |
| 3 | 285 | 277,713<br>284,048<br>287,913 | 280,254<br>287,041<br>291,841 | PVX_000820<br>PVX_000815<br>PVX_000810 | Flap endonuclease<br>SIAP1<br>PLP1 | PF3D7_0408500<br>PF3D7_0408600<br>PF3D7_0408700 |
| 7 | 1,085 | 1,080,758<br>1,085,365<br>1,088,721<br>1,089,698 | 1,083,176<br>1,087,252<br>1,089,632<br>1,090,833 | PVX_099855<br>PVX_099860<br>PVX_099870<br>PVX_099875 | Hypothetical<br>Hypothetical<br>Hypothetical<br>Hypothetical | PF3D7_0928200<br>PF3D7_0928300<br>PF3D7_0928400<br>PF3D7_0928500 |
| 7 | 1,355 | 1,353,104<br>1,356,687 | 1,354,428<br>1,360,472 | PVX_086945<br>PVX_086940 | Exported<br>Hypothetical | PF3D7_0935500<br>n.a. |
| 7 | 1,385 | 1,380,491<br>1,383,396<br>1,388,280 | 1,381,592<br>1,383,959<br>1,389,756 | PVX_086920<br>PVX_086915<br>PVX_086910 | Hypothetical<br>ETRAMP<br>PHIST | n.a.<br>n.a.<br>n.a. |
| 9 | 1,535 | 1,515,724 | 1,524,557 | PVX_092570 | ApiAP2 TF | PF3D7_1139300 |
| 11 | 175 | 169,826<br>172,656<br>175,850<br>179,797 | 171,495<br>174,464<br>176,663<br>183,156 | PVX_115295<br>PVX_115290<br>PVX_115285<br>PVX_115280 | Hypothetical<br>SRP receptor<br>NDP kinase<br>Hypothetical | PF3D7_1366700<br>PF3D7_1366600<br>PF3D7_1366500<br>PF3D7_1366400 |
| 11 | 1,075<br>1,085 | 1072903<br>1080139 | 1073769<br>1086780 | PVX_114310<br>PVX_114305 | Ribonuclease H2<br>Tyr kinase | PF3D7_0623900<br>PF3D7_0623800 |
| 11 | 1,115 | 1,129,262 | 1,138,672 | PVX_114260 | ApiAP2 TF | PF3D7_0622900<br>(*pfap2-sp3)* |
| 13 | 1,105 | 1,105,001 | 1,107,127 | PVX_085325 | Pv specific | n.a. |
| 13 | 1,775 | 1,773,064 | 1,777,749 | PVX_086035 | ApiAP2 TF | PF3D7_1408200<br>(*pfap2-g2*) |

Kb, kilobase; Pf, *Plasmodium falciparum*; n.a., not available.

**Supplementary Table 5**: Sequences of primers used for the generation of FISH probes and to validate that chr14 is physically intact around the domain boundary.

| Chr | Gene/Location | Primer sequence | Use |
|---|---|---|---|
| 8 | PF3D7_0800300 (*var*) | F: 5'-CGAAAGATAGTAGTGATGGT-3'<br>R: 5'-CACTTATGCATTTCCATCCA-3' | FISH |
| 12 | PF3D7_1222600 (*pfap2-g*) | F: 5'-ATGGATAATATGAATGCACCTA-3'<br>R: 5'-GTTGATAAATCACTAATAGCAC-3' | FISH |
| 14 | 1,183,922 – 1,187,279 | F: 5'- GTGTGTTAAATCCATTGATC -3'<br>R: 5'- GAAAGAATGTTGTTAAGCATCC -3' | PCR |
| 14 | 1,184,647 – 1,187,744 | F: 5'- GAGTAACTATAATATAGGTCC -3'<br>R: 5'- GCGCGATAAATATACACCACC -3' | PCR |
| 14 | 1,185,204 – 1,188,066 | F: 5'- GGTAATAGAGGATTTCAACA -3'<br>R: 5'- CGTGTACATATAAAGTGACATAC -3' | PCR |
| 14 | 1,185,589 – 1,187,279 | F: 5'- GTAGTGTACATACACTTATG -3'<br>R: 5'- GCGCGATAAATATACACCACC -3' | PCR |
| 14 | 1,185,589 – 1,188,519 | F: 5'- GTAGTGTACATACACTTATG -3'<br>R: 5'- CAATCCTCTATGTTTATCTACATC -3' | PCR |
| 14 | 1,188,381 – 1,188,599 | F: 5'- GAAACAATTTCCGATATATTTAACTCAACATAGA -3'<br>R: 5'- GAACTACCTGTGCCTCTCC -3' | Probe for Southern |

**Supplementary Movie 1: Animation of the changes in *P. falciparum* genome organization during stage transitions**

We recommend opening this file in VLC media player (http://www.videolan.org/vlc/). The video is also accessible via YouTube: https://youtu.be/fcccffs16FQ . The order of the stages shown in the video is as follows: ring, trophozoite, schizont, early gametocyte, late gametocyte and finally sporozoite.

## Supplementary References


1. Trager, W. & Jensen, J.B. Human malaria parasites in continuous culture. *Science* **193**, 673-675 (1976).
2. Ifediba, T. & Vanderberg, J.P. Complete in vitro maturation of Plasmodium falciparum gametocytes. *Nature* **294**, 364-366 (1981).
3. Adjalley, S.H., Johnston, G.L., Li, T., Eastman, R.T., Ekland, E.H., Eappen, A.G., Richman, A., Sim, B.K., Lee, M.C., Hoffman, S.L. & Fidock, D.A. Quantitative assessment of Plasmodium falciparum sexual development reveals potent transmission-blocking activity by methylene blue. *Proc Natl Acad Sci U S A* **108**, E1214-1223 (2011).
4. Lucantoni, L., Fidock, D.A. & Avery, V.M. Luciferase-Based, High-Throughput Assay for Screening and Profiling Transmission-Blocking Compounds against Plasmodium falciparum Gametocytes. *Antimicrob Agents Chemother* **60**, 2097-2107 (2016).
5. Duffy, S. & Avery, V.M. Identification of inhibitors of Plasmodium falciparum gametocyte development. *Malar J* **12**, 408 (2013).
6. Swearingen, K.E., Lindner, S.E., Shi, L., Shears, M.J., Harupa, A., Hopp, C.S., Vaughan, A.M., Springer, T.A., Moritz, R.L., Kappe, S.H. & Sinnis, P. Interrogating the Plasmodium Sporozoite Surface: Identification of Surface-Exposed Proteins and Demonstration of Glycosylation on CSP and TRAP by Mass Spectrometry-Based Proteomics. *PLoS Pathog* **12**, e1005606 (2016).
7. Andolina, C., Landier, J., Carrara, V., Chu, C.S., Franetich, J.F., Roth, A., Renia, L., Roucher, C., White, N.J., Snounou, G. & Nosten, F. The suitability of laboratory-bred Anopheles cracens for the production of Plasmodium vivax sporozoites. *Malar J* **14**, 312 (2015).
8. Brancucci, N.M., Bertschi, N.L., Zhu, L., Niederwieser, I., Chin, W.H., Wampfler, R., Freymond, C., Rottmann, M., Felger, I., Bozdech, Z. & Voss, T.S. Heterochromatin protein 1 secures survival and transmission of malaria parasites. *Cell Host Microbe* **16**, 165-176 (2014).
9. Bunnik, E.M., Polishko, A., Prudhomme, J., Ponts, N., Gill, S.S., Lonardi, S. & Le Roch, K.G. DNA-encoded nucleosome occupancy is associated with transcription levels in the human malaria parasite Plasmodium falciparum. *BMC Genomics* **15**, 347 (2014).
10. Miao, J. & Cui, L. Rapid isolation of single malaria parasite-infected red blood cells by cell sorting. *Nat Protoc* **6**, 140-146 (2011).
11. Langmead, B. & Salzberg, S.L. Fast gapped-read alignment with Bowtie 2. *Nat Methods* **9**, 357-359 (2012).
12. Robinson, J.T., Thorvaldsdottir, H., Winckler, W., Guttman, M., Lander, E.S., Getz, G. & Mesirov, J.P. Integrative genomics viewer. *Nat Biotechnol* **29**, 24-26 (2011).
13. Thorvaldsdottir, H., Robinson, J.T. & Mesirov, J.P. Integrative Genomics Viewer (IGV): high-performance genomics data visualization and exploration. *Brief Bioinform* **14**, 178-192 (2013).
14. Quinlan, A.R. & Hall, I.M. BEDTools: a flexible suite of utilities for comparing genomic features. *Bioinformatics* **26**, 841-842 (2010).
15. Ay, F., Bunnik, E.M., Varoquaux, N., Bol, S.M., Prudhomme, J., Vert, J.P., Noble, W.S. & Le Roch, K.G. Three-dimensional modeling of the P. falciparum genome during the erythrocytic cycle reveals a strong connection between genome architecture and gene expression. *Genome Res* **24**, 974-988 (2014).
16. Cournac, A., Marie-Nelly, H., Marbouty, M., Koszul, R. & Mozziconacci, J. Normalization of a chromosomal contact map. *BMC Genomics* **13**, 436 (2012).
17. Yaffe, E. & Tanay, A. Probabilistic modeling of Hi-C contact maps eliminates systematic biases to characterize global chromosomal architecture. *Nat Genet* **43**, 1059-1065 (2011).

18. Imakaev, M., Fudenberg, G., McCord, R.P., Naumova, N., Goloborodko, A., Lajoie, B.R., Dekker, J. & Mirny, L.A. Iterative correction of Hi-C data reveals hallmarks of chromosome organization. *Nature methods* **9**, 999-1003 (2012).
19. Derrien, T., Estelle, J., Marco Sola, S., Knowles, D.G., Raineri, E., Guigo, R. & Ribeca, P. Fast computation and applications of genome mappability. *PLoS One* **7**, e30377 (2012).
20. Dudchenko, O., Batra, S.S., Omer, A.D., Nyquist, S.K., Hoeger, M., Durand, N.C., Shamim, M.S., Machol, I., Lander, E.S., Aiden, A.P. & Aiden, E.L. De novo assembly of the Aedes aegypti genome using Hi-C yields chromosome-length scaffolds. *Science* **356**, 92-95 (2017).
21. Witten, D.M. & Noble, W.S. On the assessment of statistical significance of three-dimensional colocalization of sets of genomic elements. *Nucleic acids research* **40**, 3849-3855 (2012).
22. Paulsen, J., Lien, T.G., Sandve, G.K., Holden, L., Borgan, O., Glad, I.K. & Hovig, E. Handling realistic assumptions in hypothesis testing of 3D co-localization of genomic elements. *Nucleic Acids Res* **41**, 5164-5174 (2013).
23. Anders, S. & Huber, W. Differential expression analysis for sequence count data. *Genome Biol* **11**, R106 (2010).
24. Robinson, M.D., McCarthy, D.J. & Smyth, G.K. edgeR: a Bioconductor package for differential expression analysis of digital gene expression data. *Bioinformatics* **26**, 139-140 (2010).
25. Drewe, P., Stegle, O., Hartmann, L., Kahles, A., Bohnert, R., Wachter, A., Borgwardt, K. & Ratsch, G. Accurate detection of differential RNA processing. *Nucleic Acids Res* **41**, 5189-5198 (2013).
26. Lun, A.T. & Smyth, G.K. diffHic: a Bioconductor package to detect differential genomic interactions in Hi-C data. *BMC Bioinformatics* **16**, 258 (2015).
27. Benjamini, Y. & Yekutieli, D. The control of the false discovery rate in multiple testing under dependency. *Ann. Statist.* **29**, 1165-1188 (2001).
28. Varoquaux, N., Ay, F., Noble, W.S. & Vert, J.P. A statistical approach for inferring the 3D structure of the genome. *Bioinformatics* **30**, i26-33 (2014).
29. Halko, N., Martinsson, P.-G. & Tropp, J.A. Finding structure with randomness: Probabilistic algorithms for constructing approximate matrix decompositions. *SIAM Reviews* **53**, 217-288 (2011).
30. Bach, F.R. & Jordan, M.I. Kernel independent component analysis. *Journal of Machine Learning Research* **3**, 1-48 (2003).
