# Supplementary material for: Changes in genome organization of parasite-specific gene families during the Plasmodium transmission stages
Source: Nat Commun. 2018 May 15;9:1910. doi: 10.1038/s41467-018-04295-5 (PMC5954139; doi:10.1038/s41467-018-04295-5)
Supplement: Supplementary file 2 — Description of Additional Supplementary Files [file 41467_2018_4295_MOESM2_ESM.pdf]

Description of Additional Supplementary Files

File Name: Supplementary Data 1
Description: Chromosome visualizations for P. falciparum stages.

File Name: Supplementary Data 2
Description: Chromosome visualizations for P. vivax sporozoites.

File Name: Supplementary Data 3
Description: Loci that show a two-fold or larger difference in contacts between two stages with a false discovery rate of 1%.

File Name: Supplementary Data 4
Description: Significant interactions (q

File Name: Supplementary Data 5
Description: H3K9me3 ChIP-seq results.

File Name: Supplementary Movie 1
Description: Animation of the changes in P. falciparum genome organization during stage transitions
