# Supplementary material for: Changes in genome organization of parasite-specific gene families during the Plasmodium transmission stages
Source: Nat Commun. 2018 May 15;9:1910. doi: 10.1038/s41467-018-04295-5 (PMC5954139; doi:10.1038/s41467-018-04295-5)

Supplementary Data 1: Chromosome visualizations for *P. falciparum* stages. For each chromosome, we generated three pages of figures for the various stages and parasite strains assessed. On the first page are the ring, trophozoite, and schizont stage from our previous study re-analyzed using the same pipeline as the new stages. On the second page are stage II/III gametocytes, stage IV/V gametocytes and sporozoites. On the third page are the wild-type ring stage (same as on the first page, but repeated here to facilitate the comparison with the HP1 strains), the ring stage of the HP1-tagged strain, and the ring stage of the HP1-knockdown strain. Within each page, the top row shows the raw contact count matrices at 10 kb resolution, the second row shows the ICE-normalized contact count matrices at 10 kb resolution, and the third row shows matrices of the fit-hi-c P-values assigned to the contacts. In these three matrices, the location of the centromere is indicated by a dashed black line and the location of the virulence gene clusters in indicated by a yellow box. Unmappable regions are colored in grey. The fourth row shows the pairwise Euclidean distance matrices derived from the 3D model, and the bottom row shows the inferred configuration in 3D with light blue spheres indicating centromeres, white spheres indicating telomeres and purple regions indicating the location of virulence gene clusters. Of note, we obtained zero mapped sequence reads for chr4:1-40,000 and chr5:1,310,000-1,343,557 in stage II/III gametocytes, suggesting that these regions are deleted in the *P. falciparum* NF54^{Pfs16} reporter gene line used for this experiment.

Chromosome 1

Ring | Trophozoite | Schizont

Raw counts

ICE normalized counts

fit-hi-c P-values

Euclidean distance

Structure

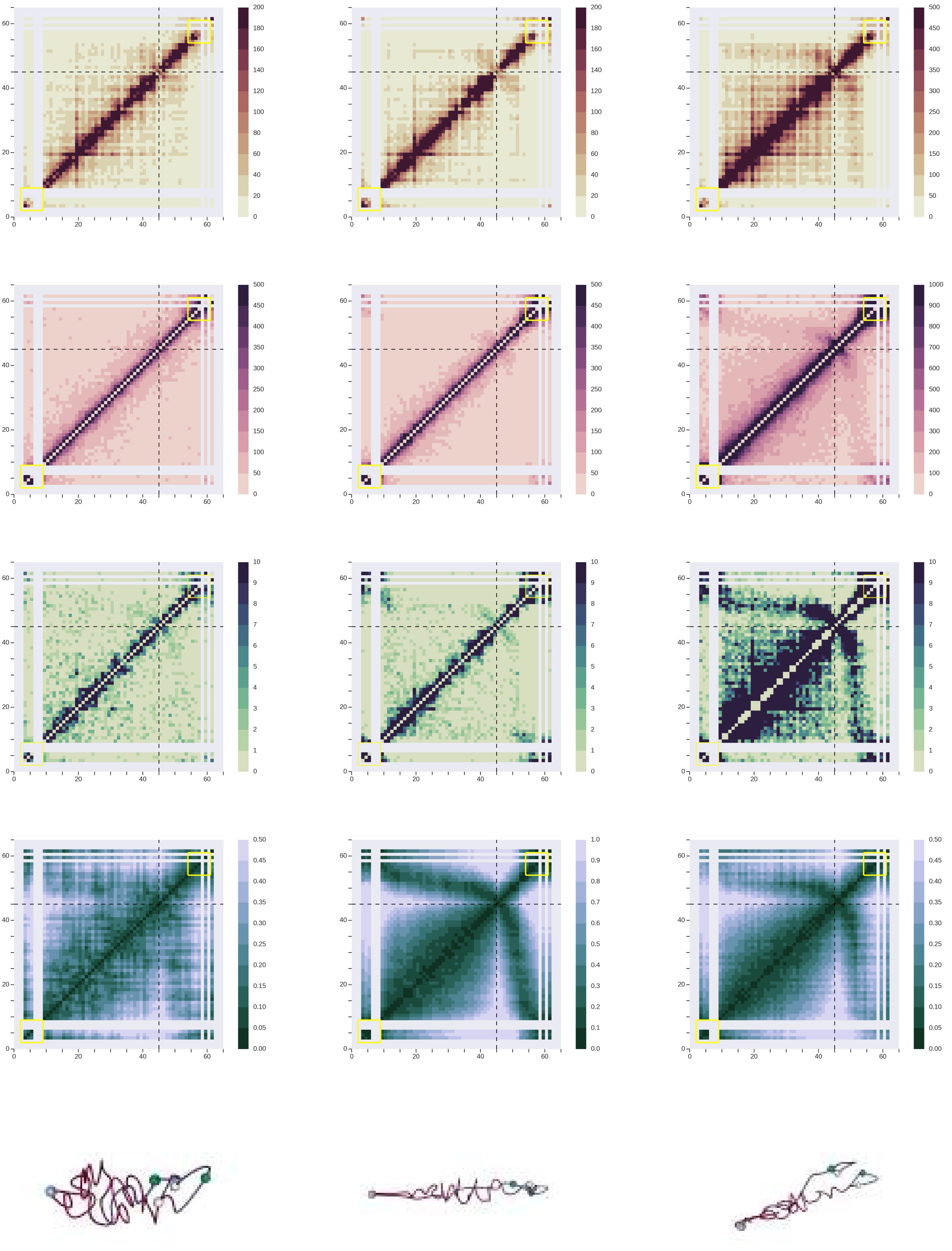

Chromosome 1

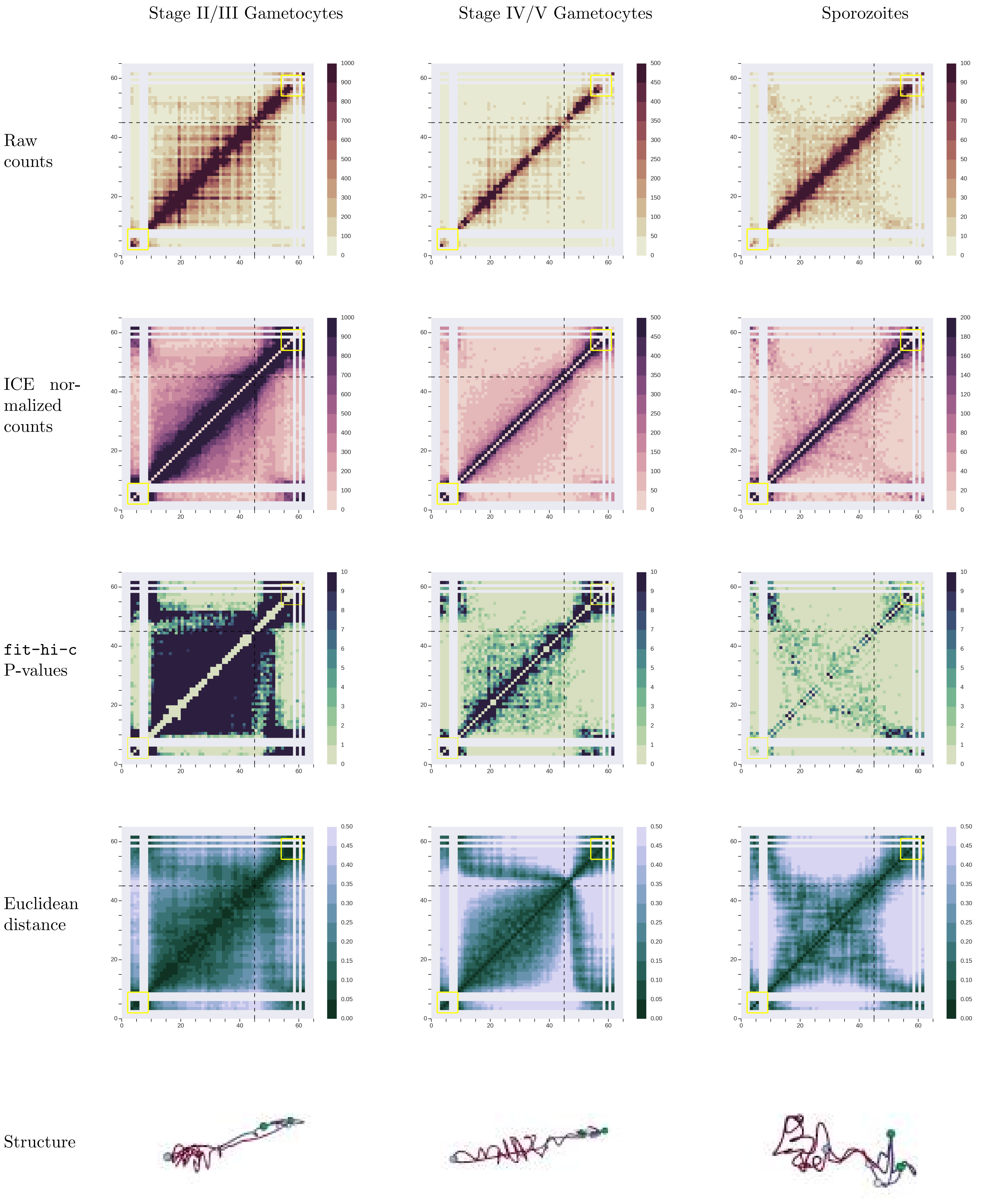

Chromosome 1

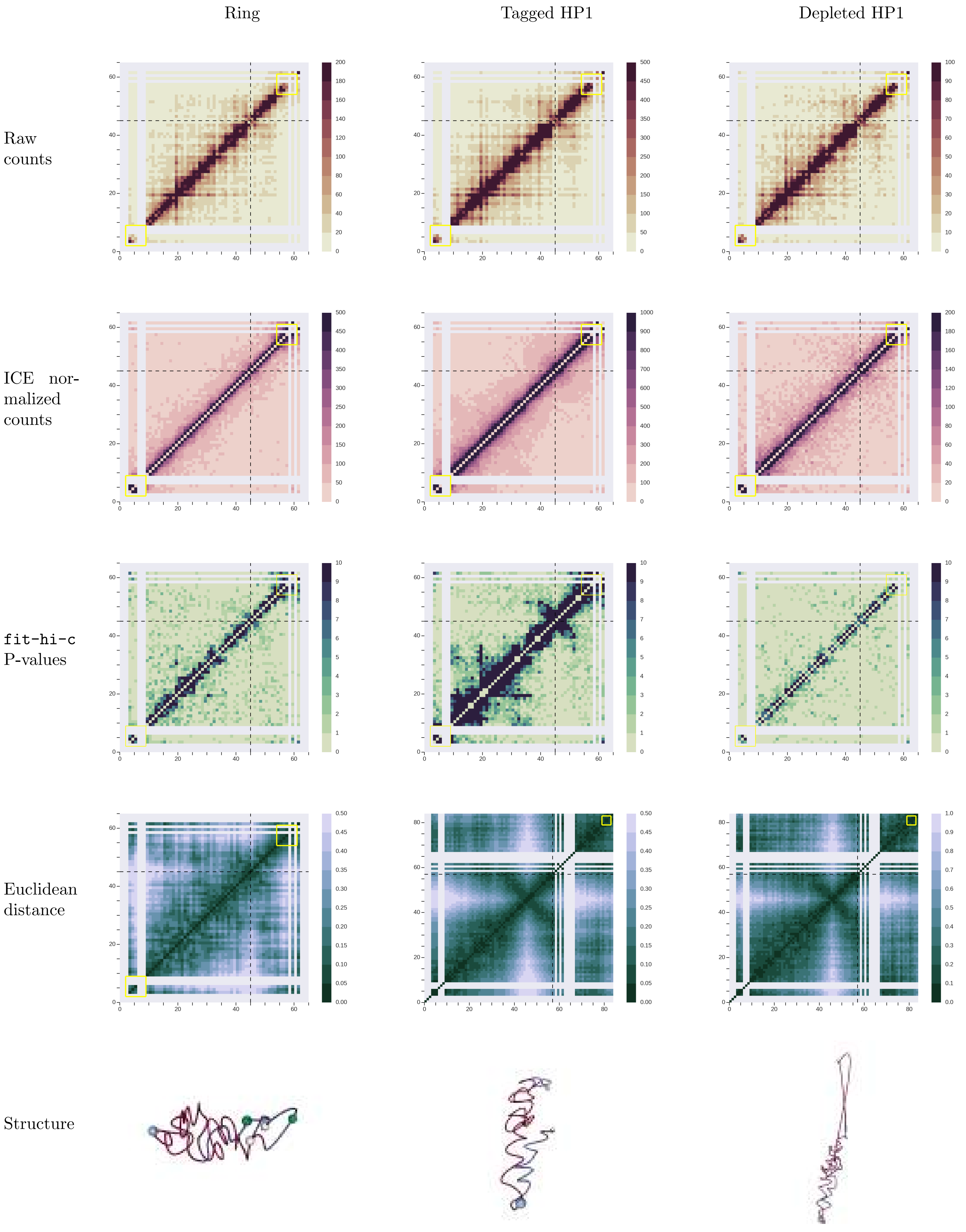

Chromosome 2

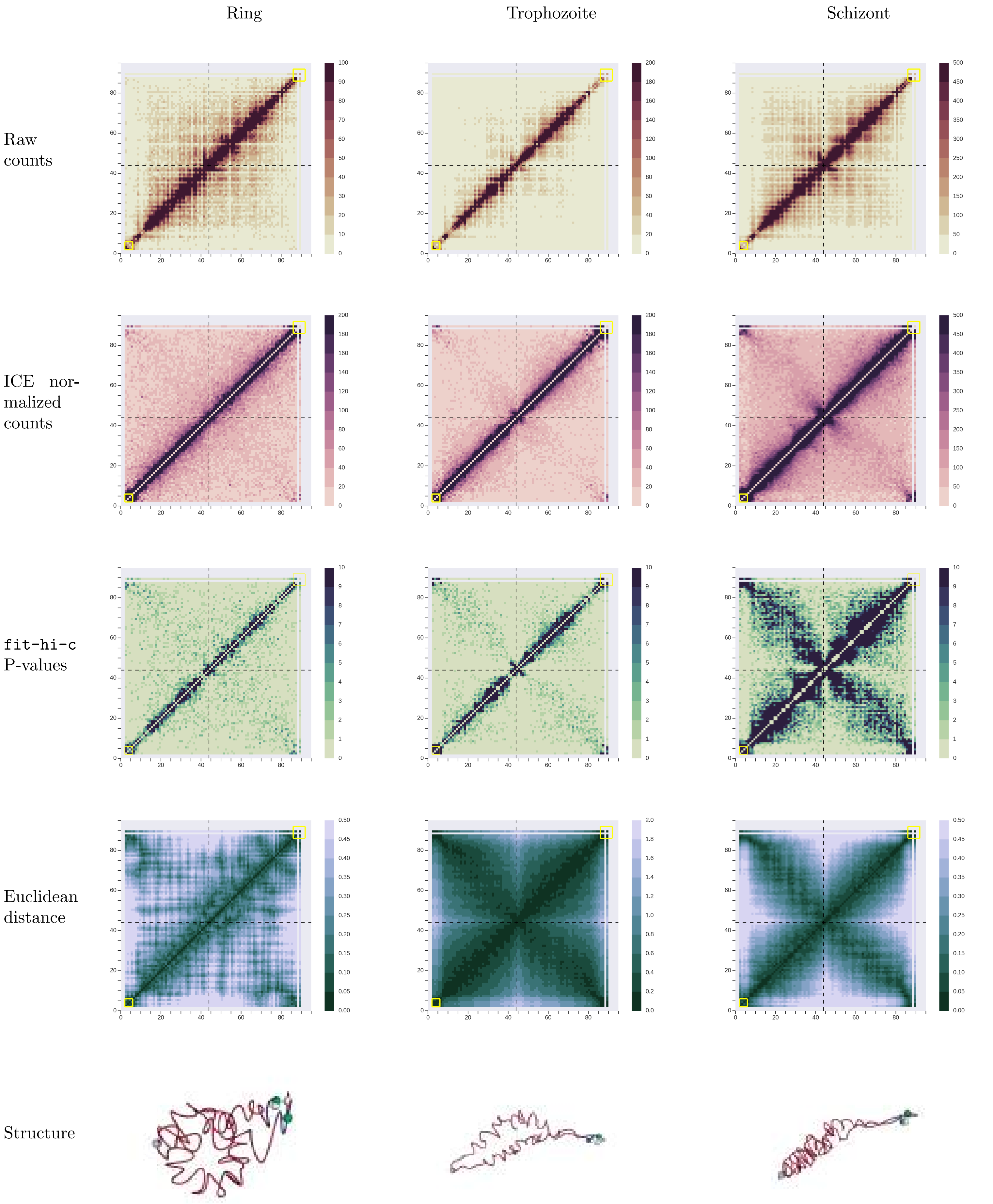

Chromosome 2

Stage II/III Gametocytes | Stage IV/V Gametocytes | Sporozoites

Raw counts

ICE normalized counts

`fit-hi-c` P-values

Euclidean distance

Structure

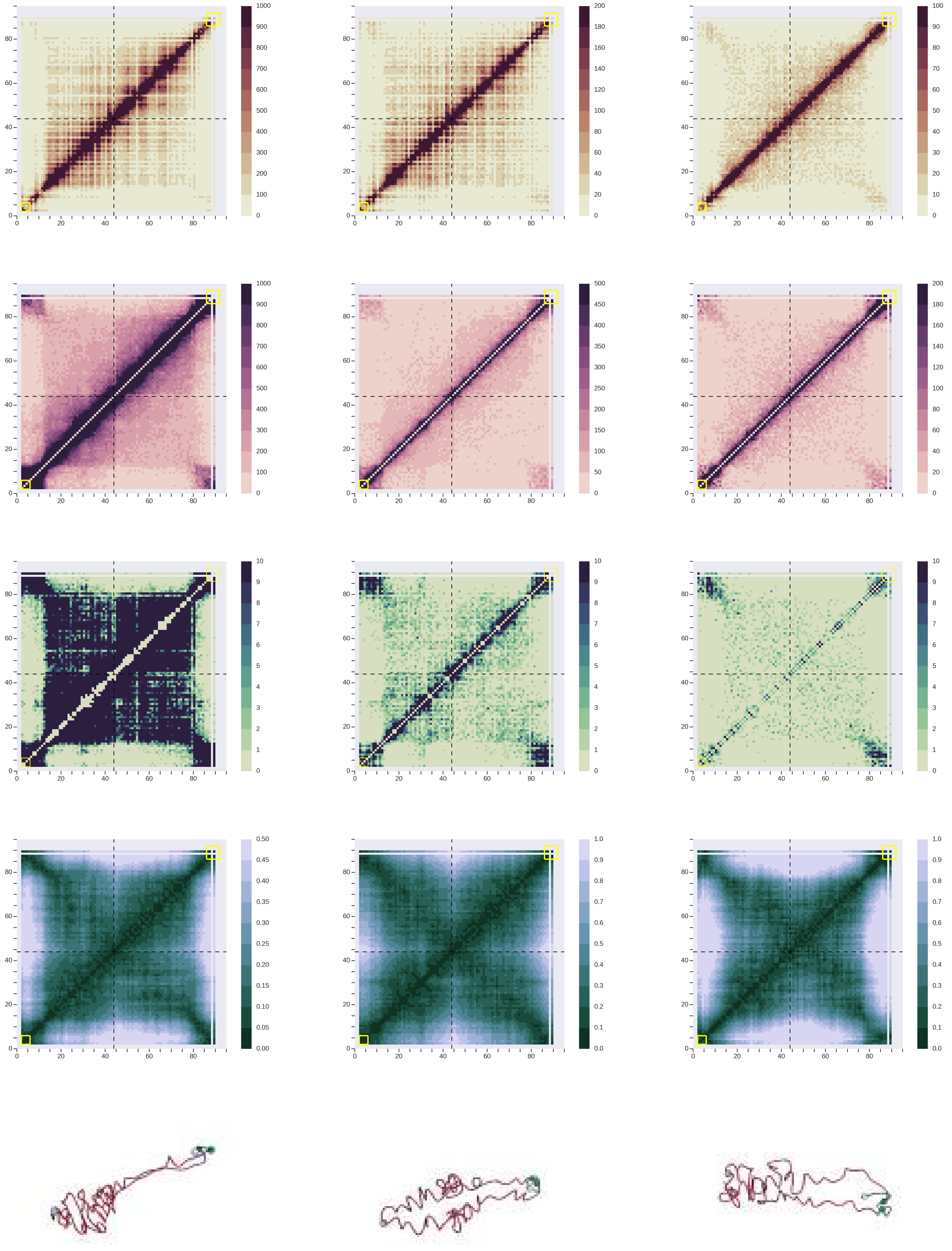

Chromosome 2

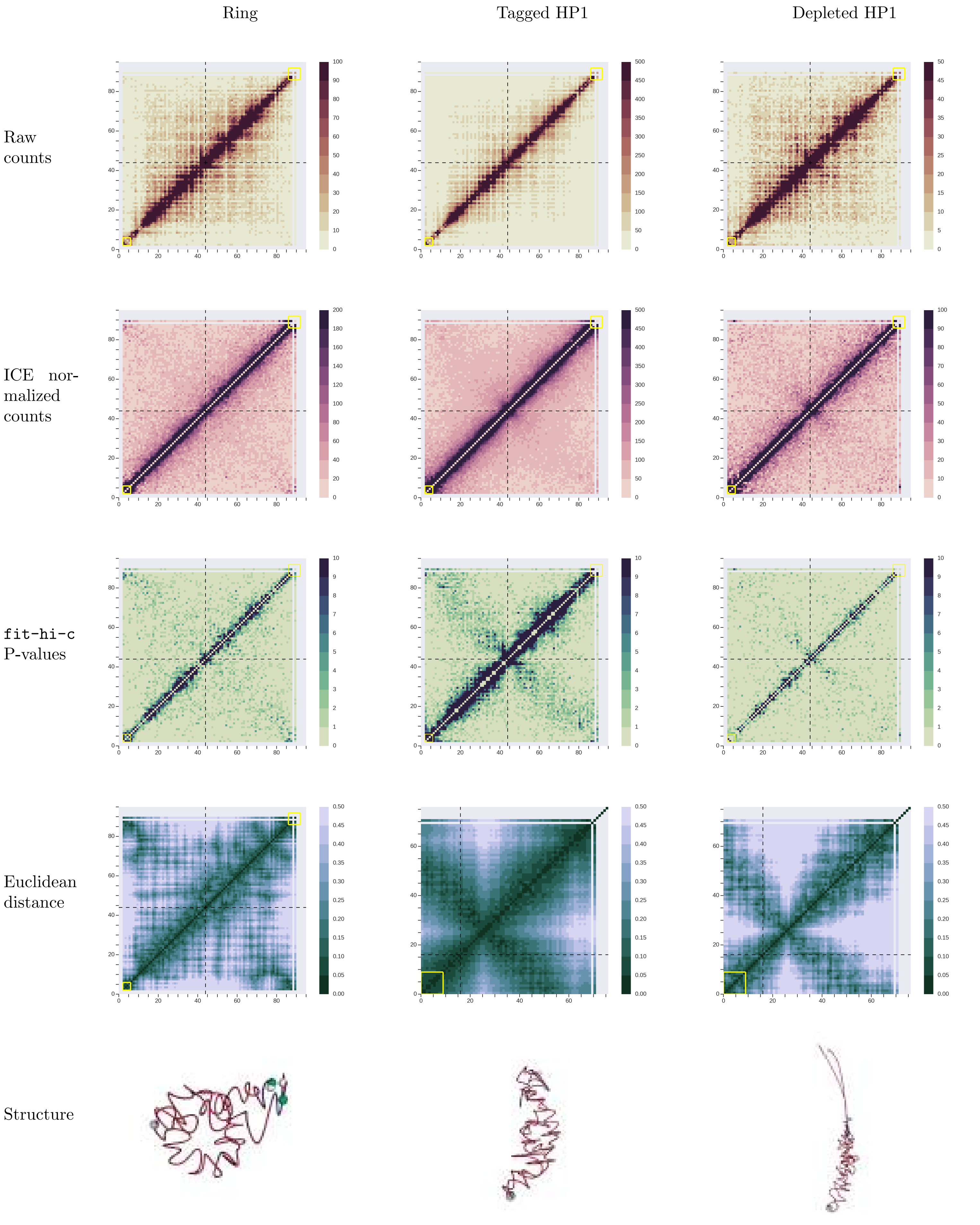

Chromosome 3

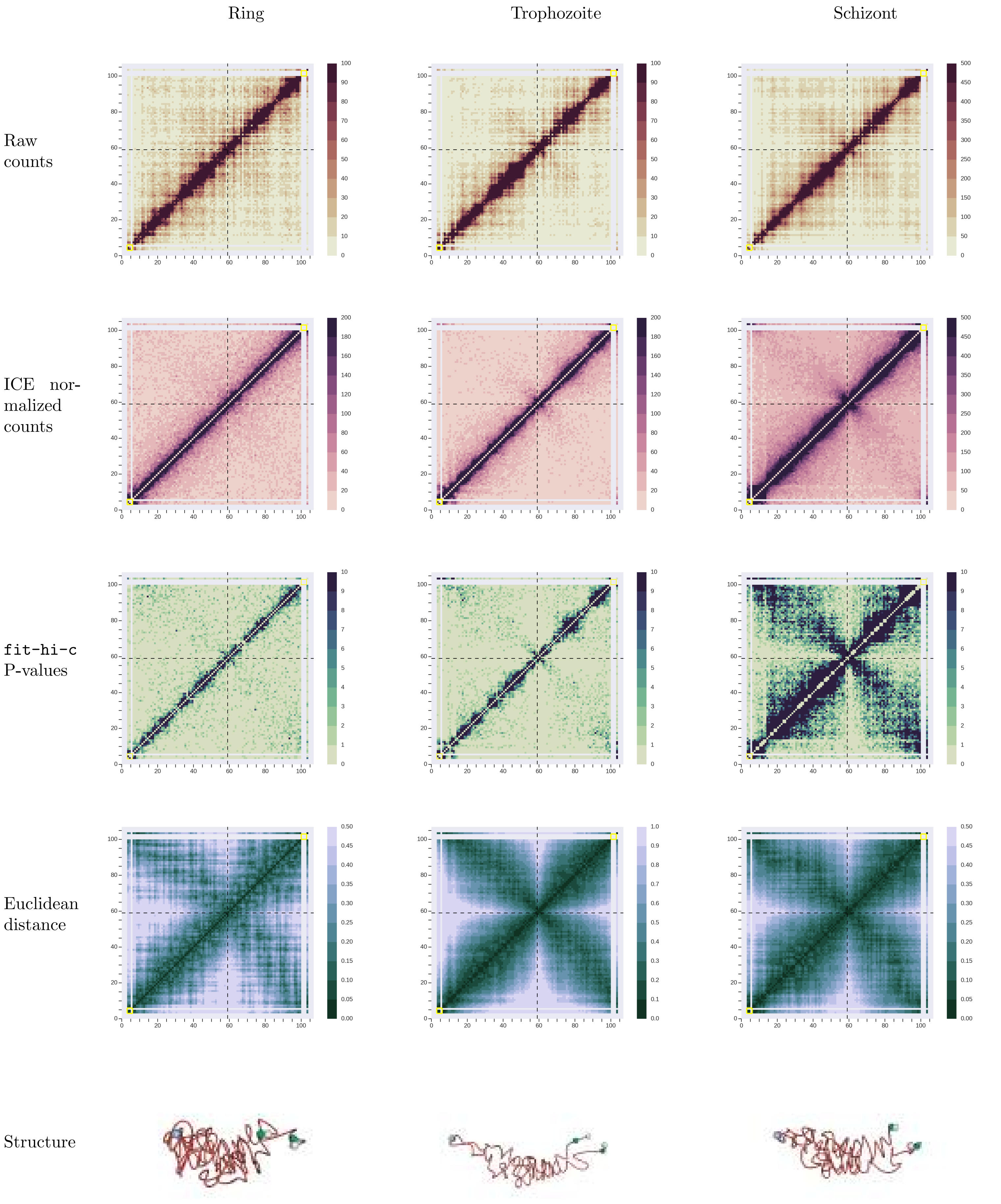

Chromosome 3

Stage II/III Gametocytes | Stage IV/V Gametocytes | Sporozoites

Raw counts

ICE normalized counts

`fit-hi-c` P-values

Euclidean distance

Structure

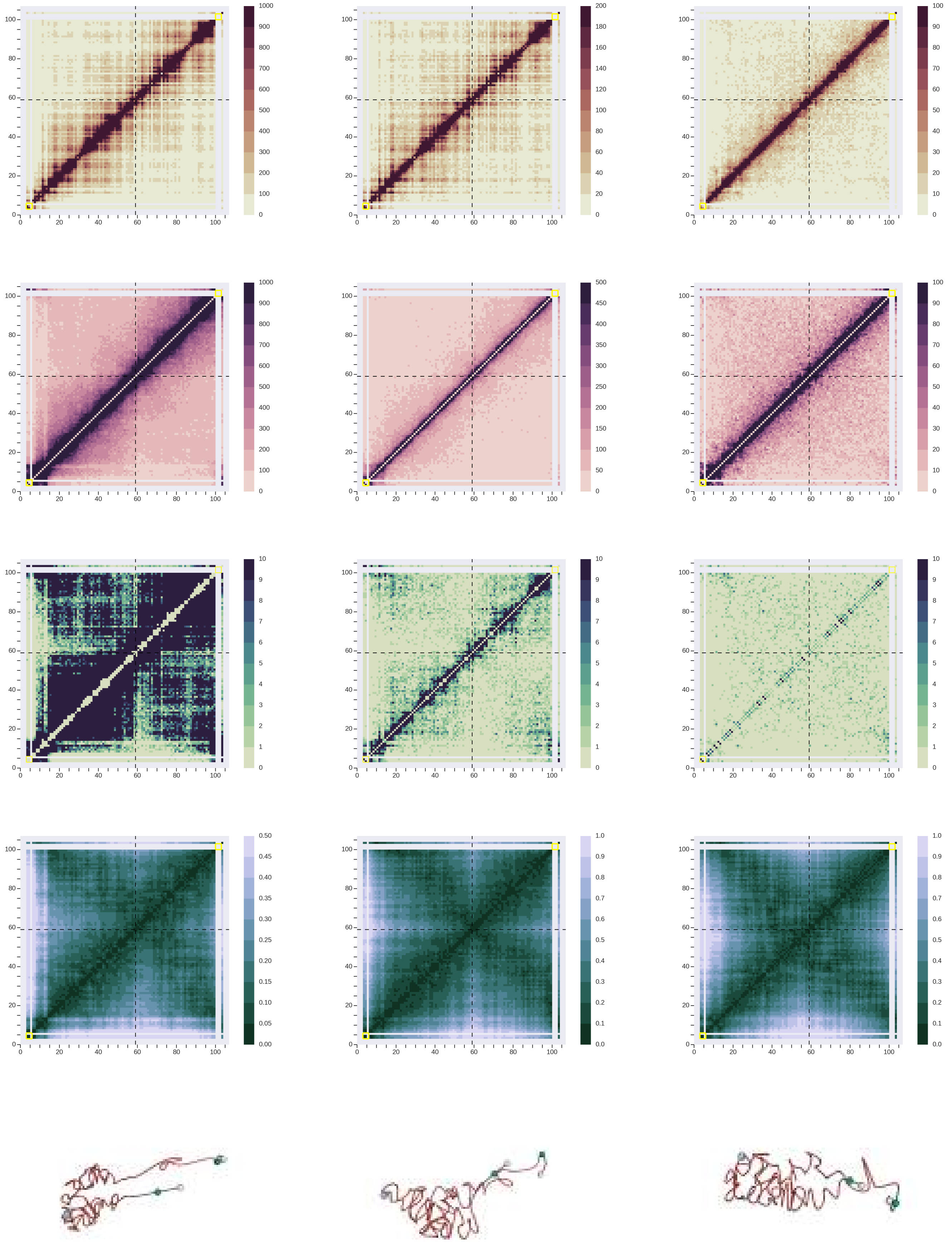

Chromosome 3

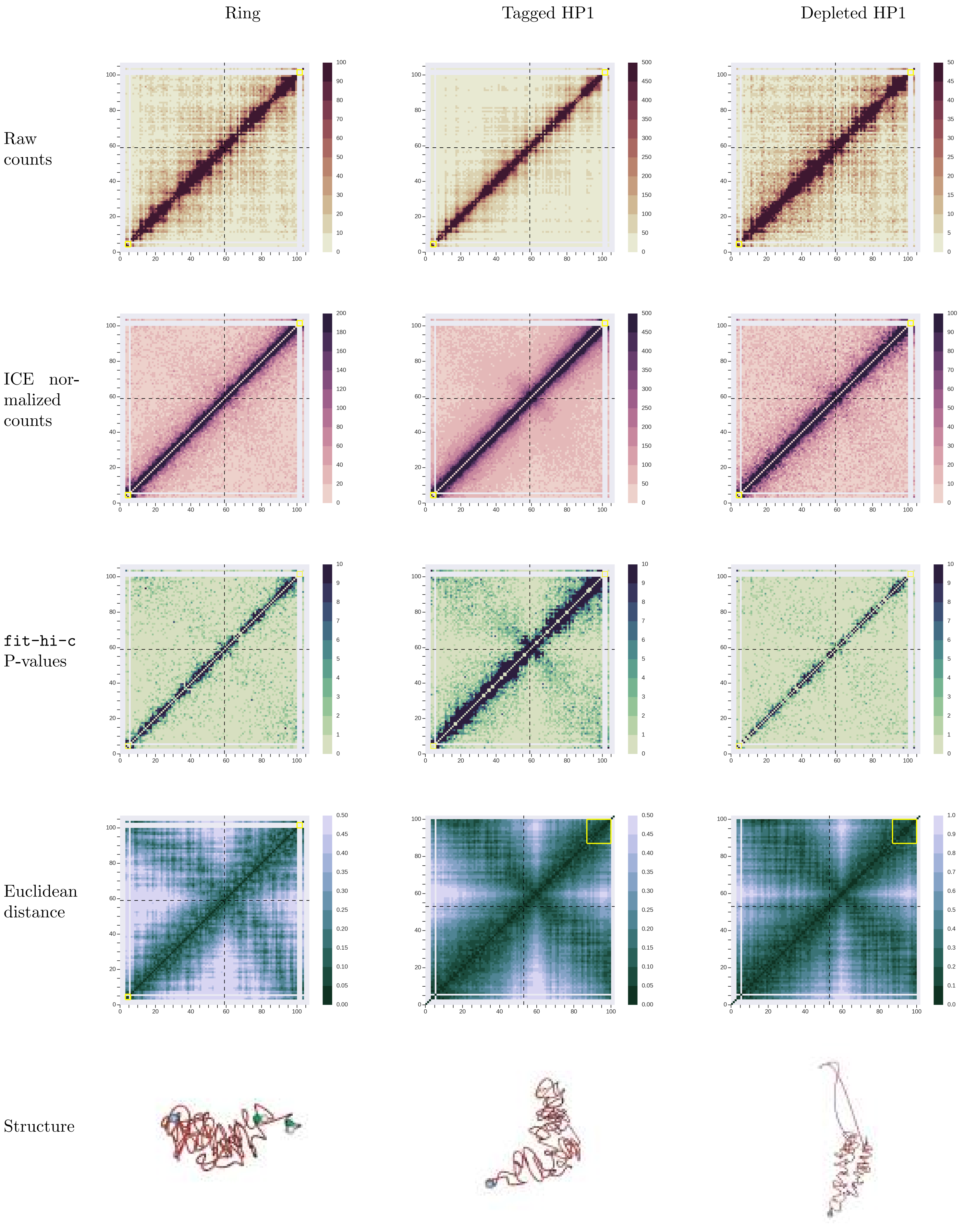

Chromosome 4

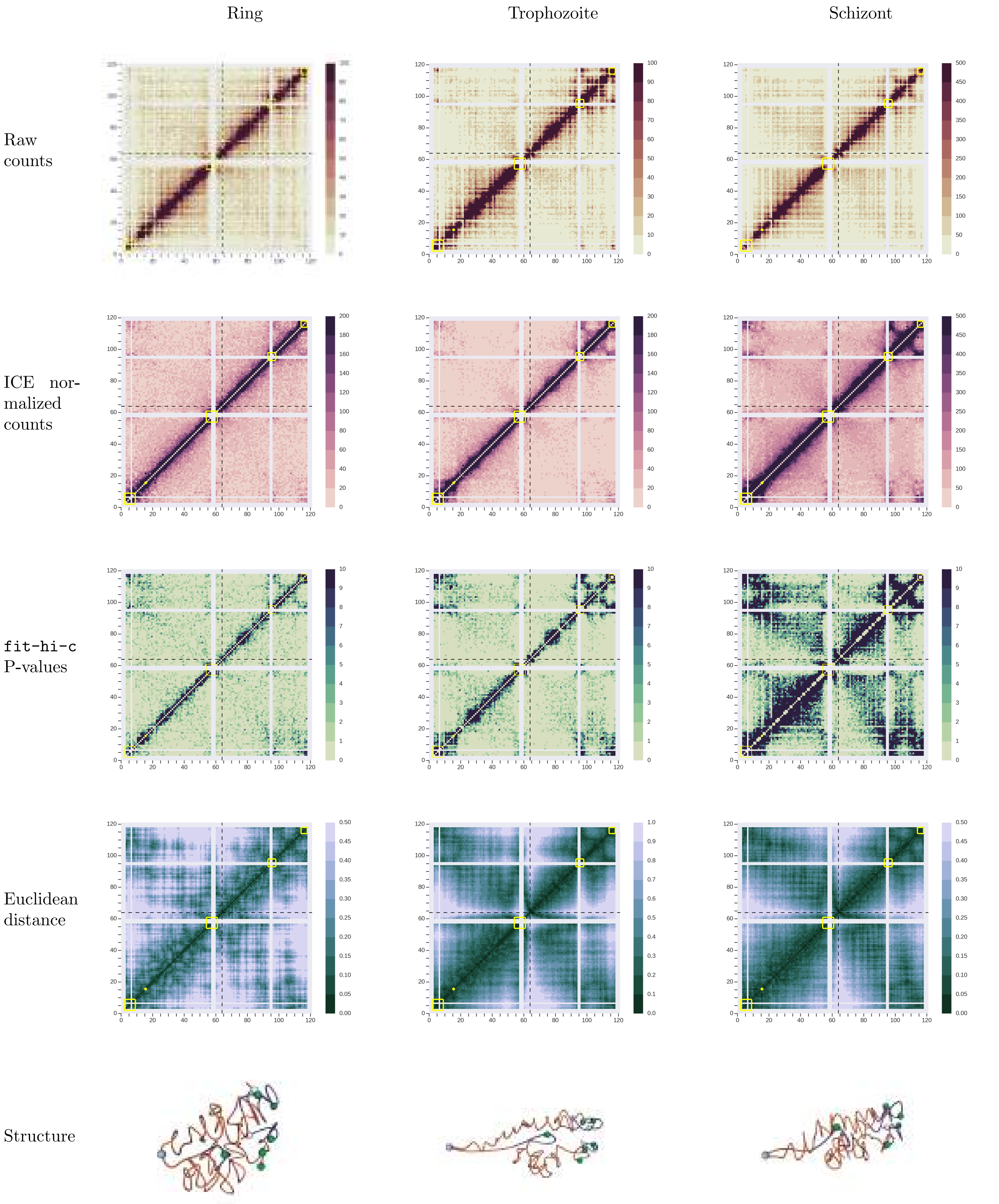

Chromosome 4

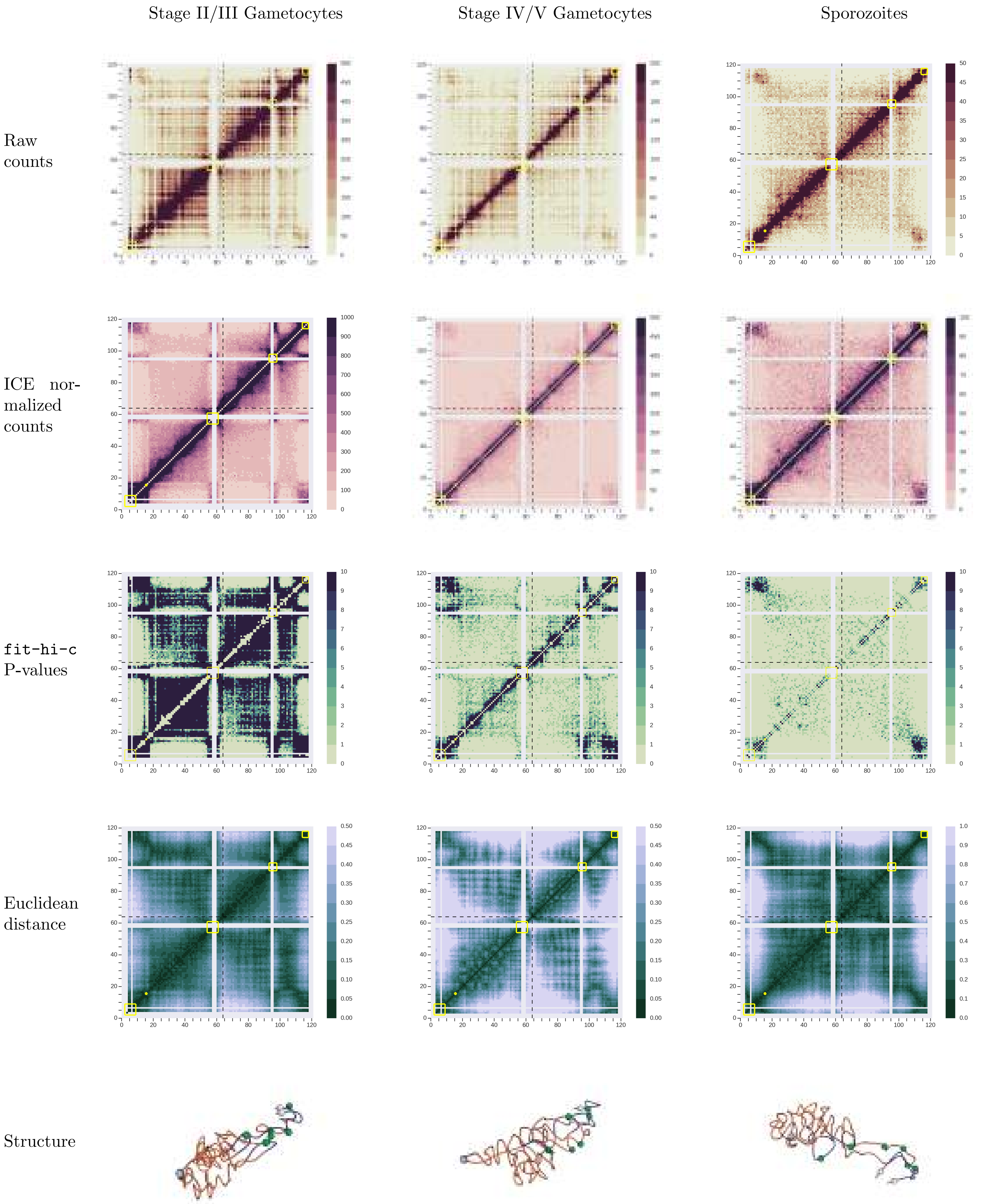

Chromosome 4

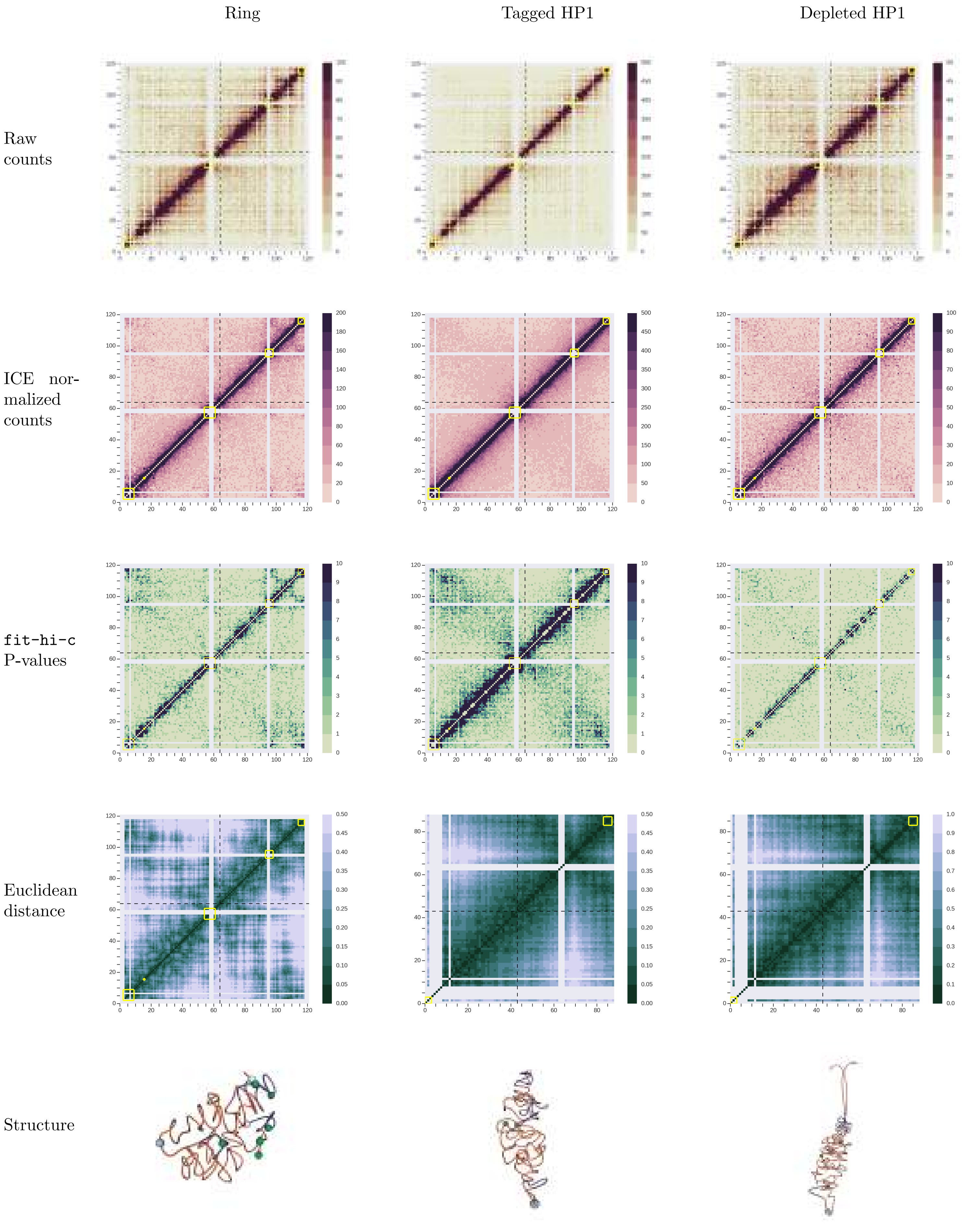

Chromosome 5

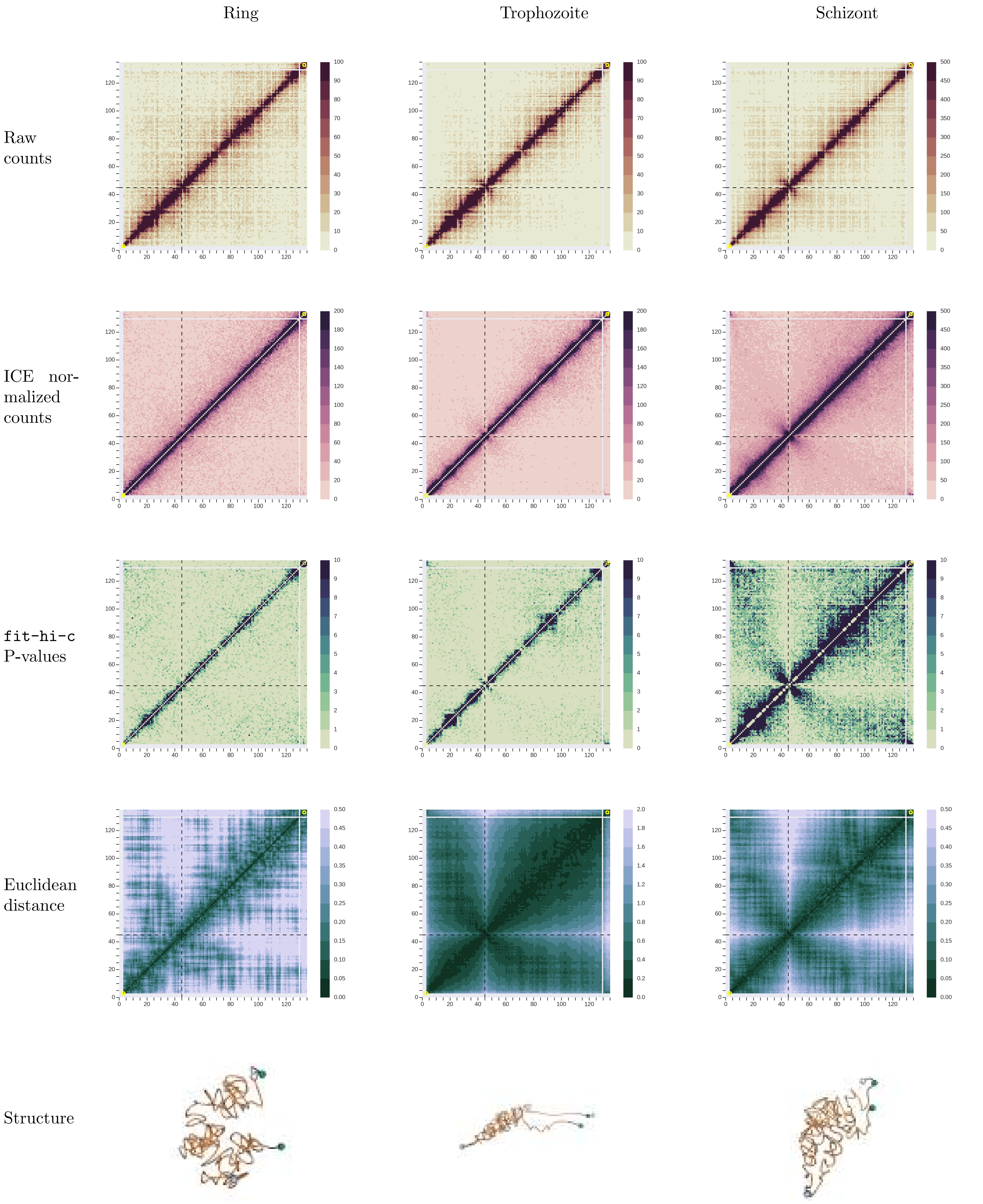

Chromosome 5

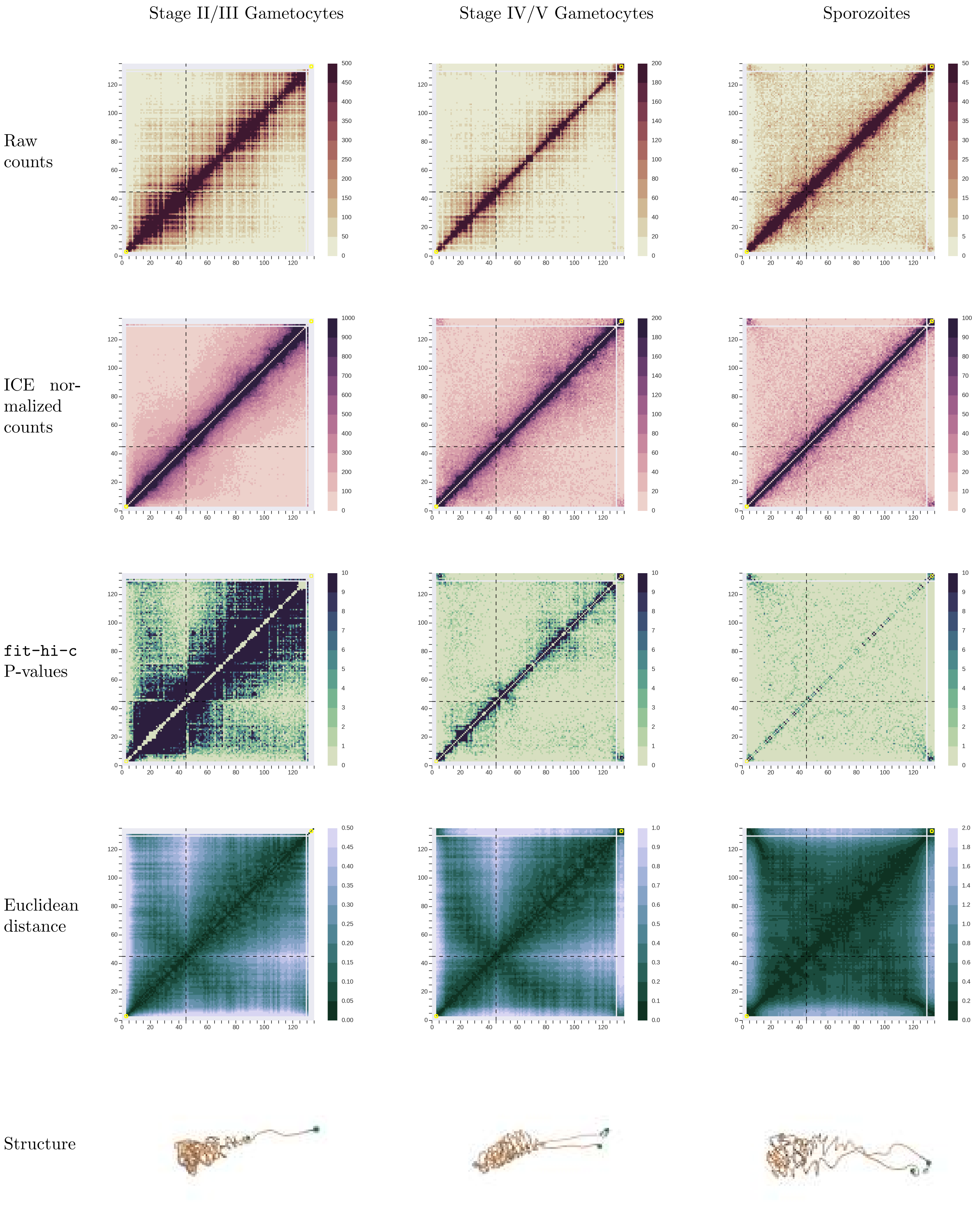

Chromosome 5

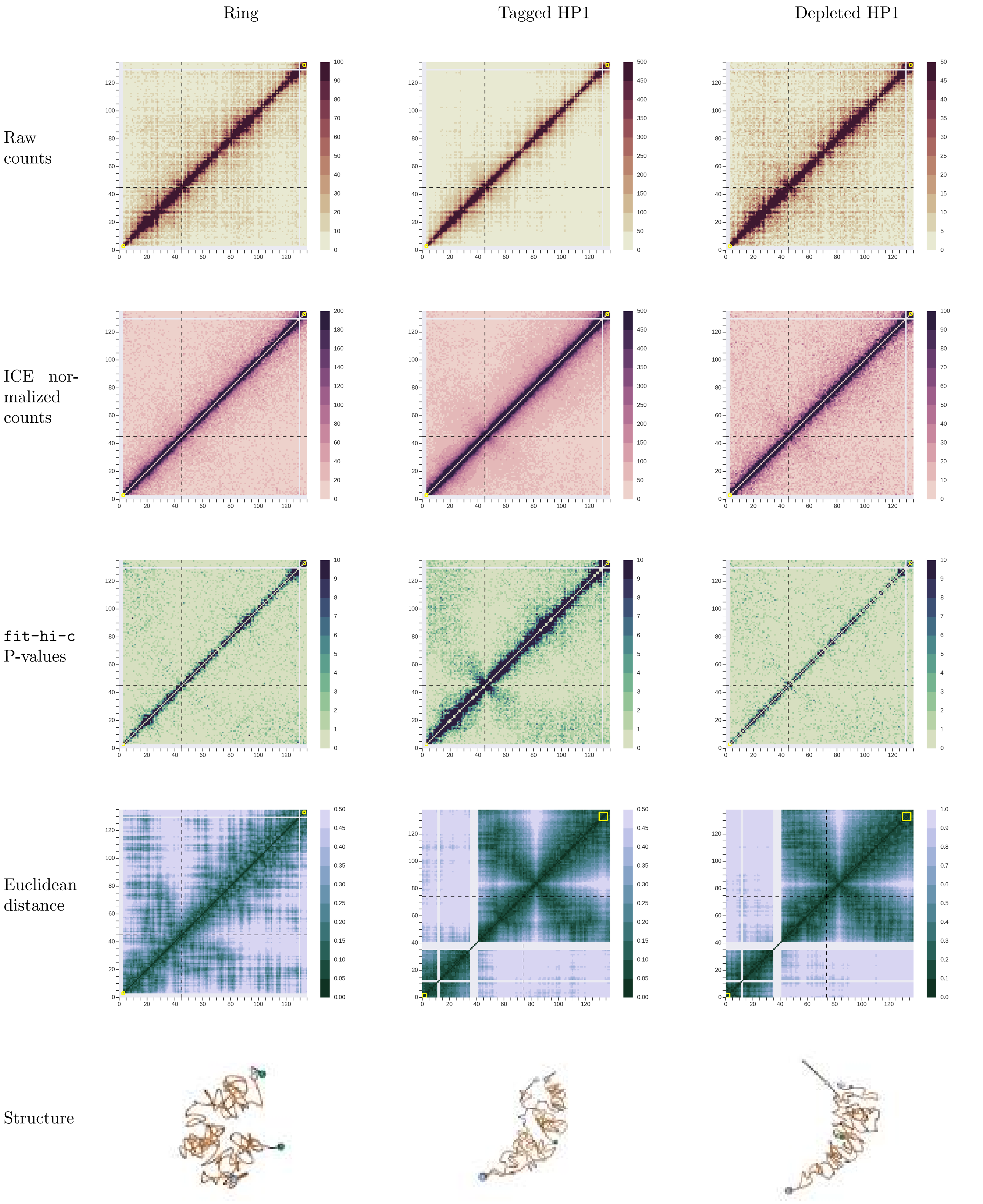

Chromosome 6

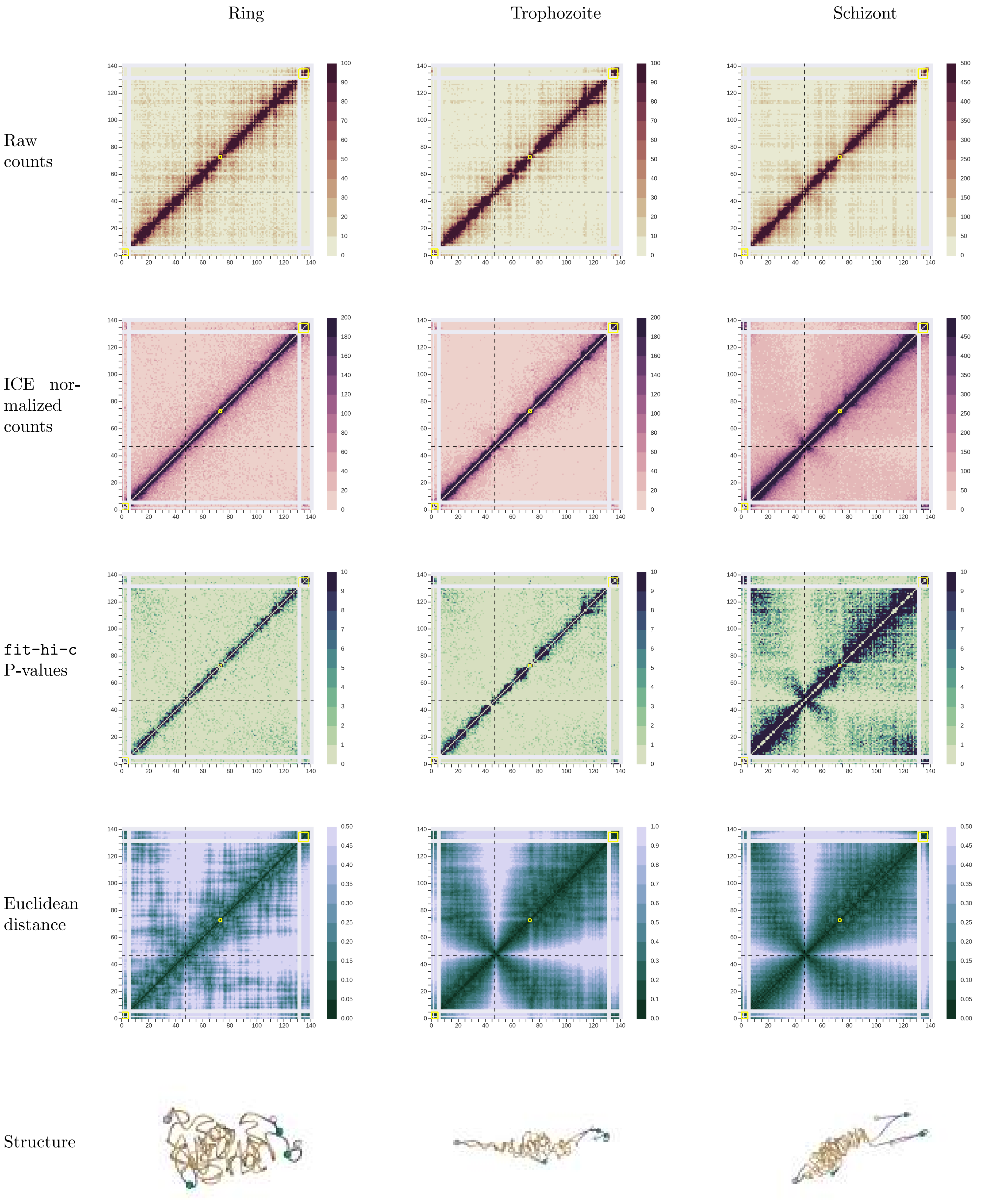

Chromosome 6

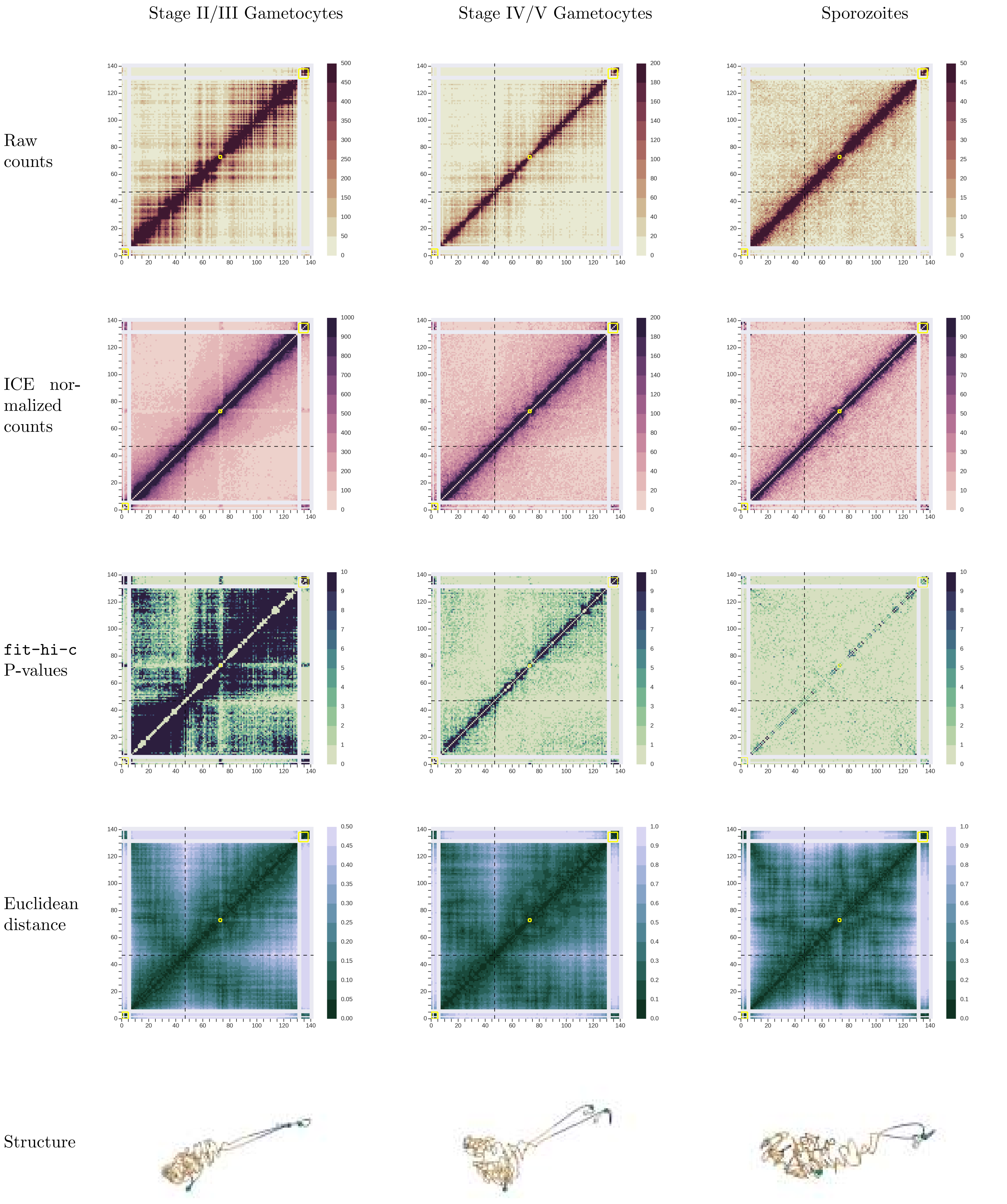

Chromosome 6

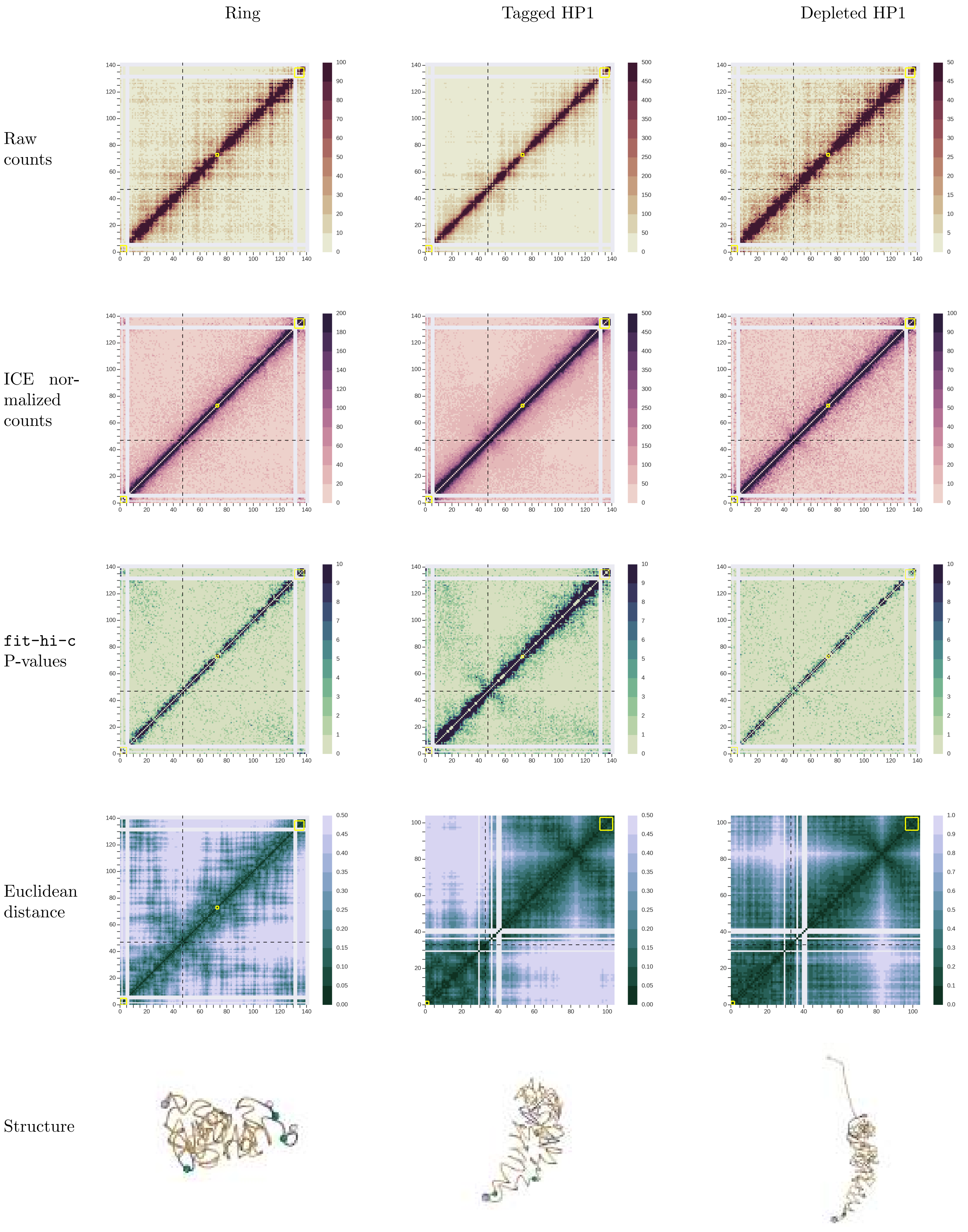

Chromosome 7

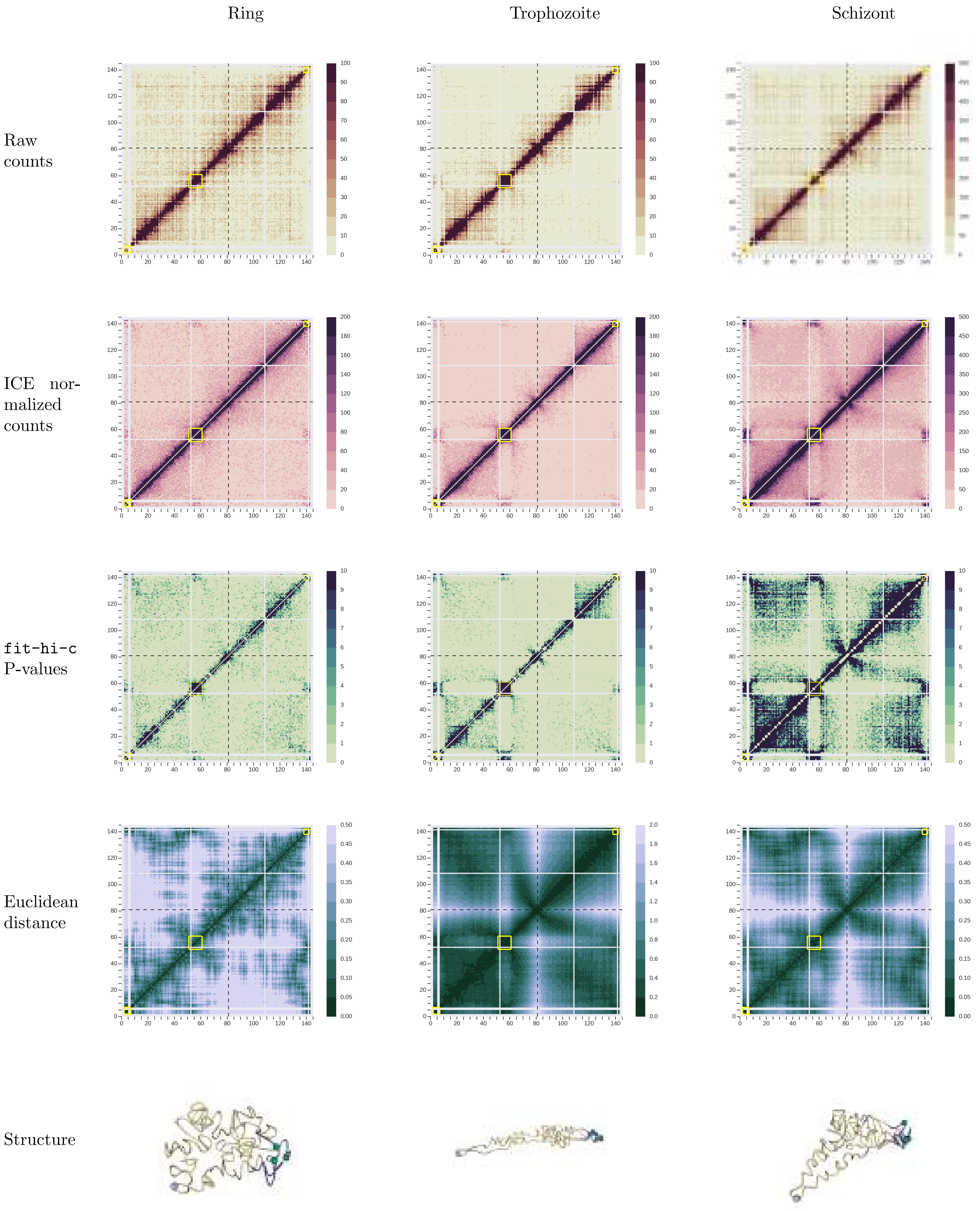

Chromosome 7

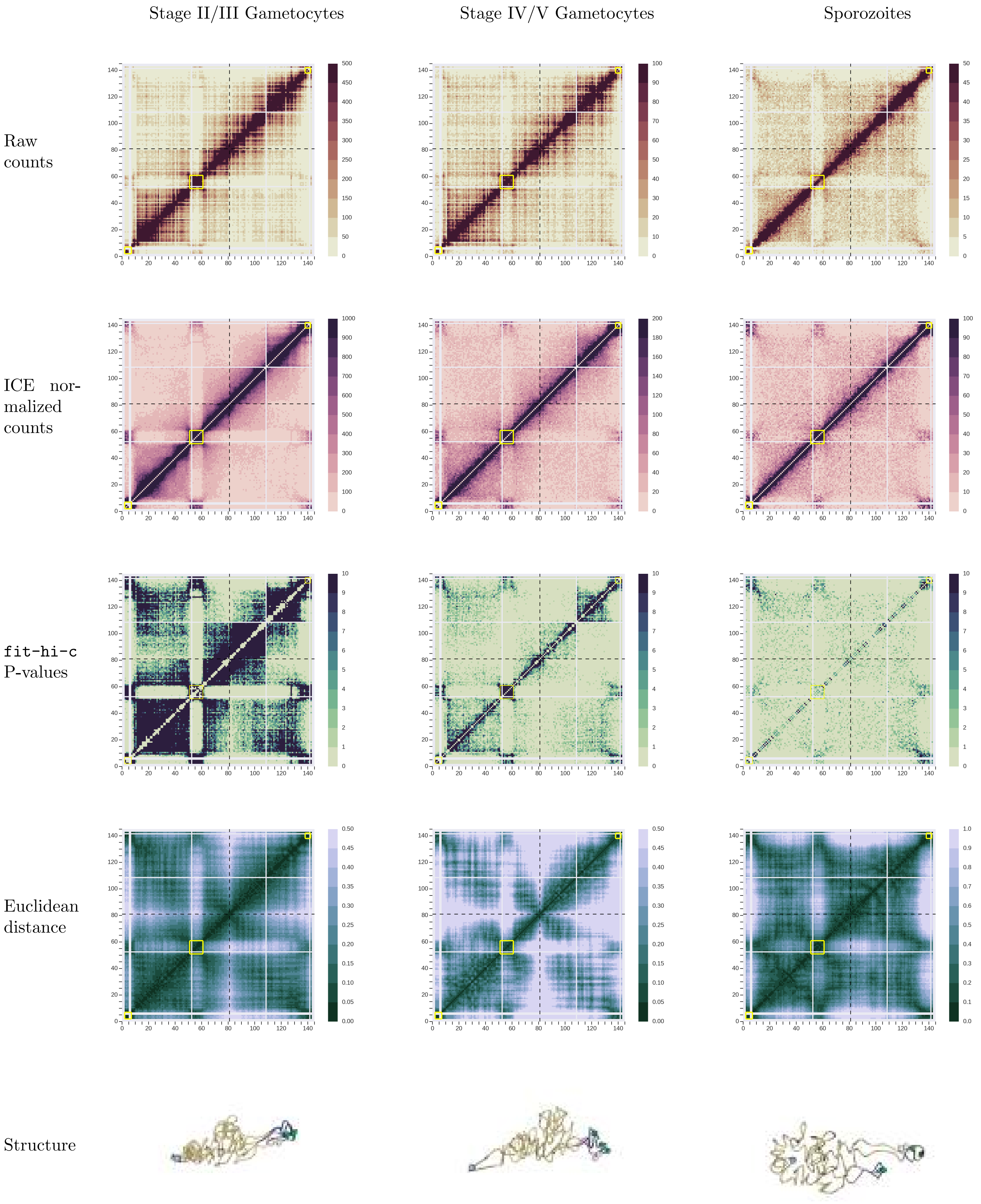

Chromosome 7

Ring | Tagged HP1 | Depleted HP1

Raw counts

ICE normalized counts

`fit-hi-c` P-values

Euclidean distance

Structure

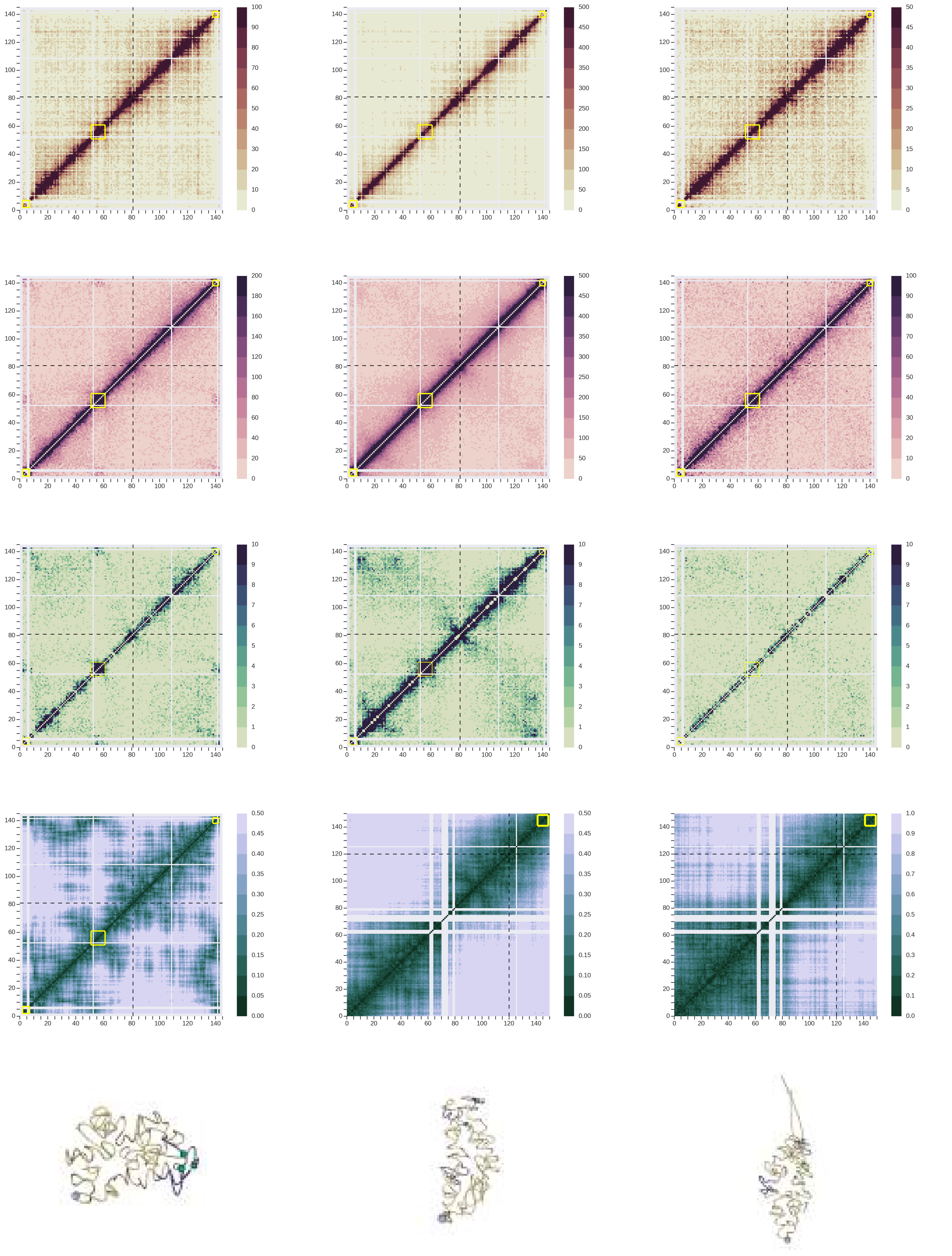

Chromosome 8

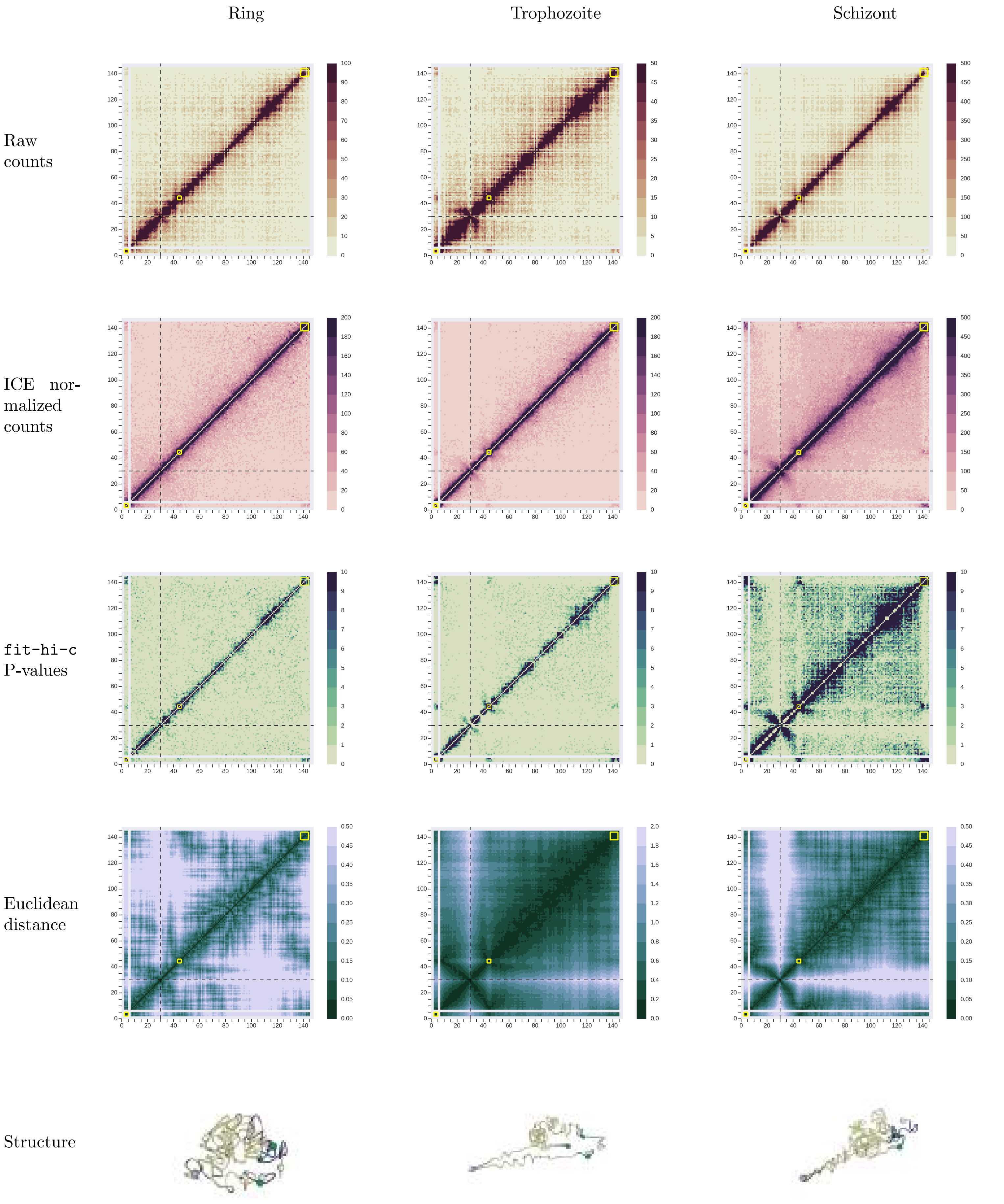

Chromosome 8

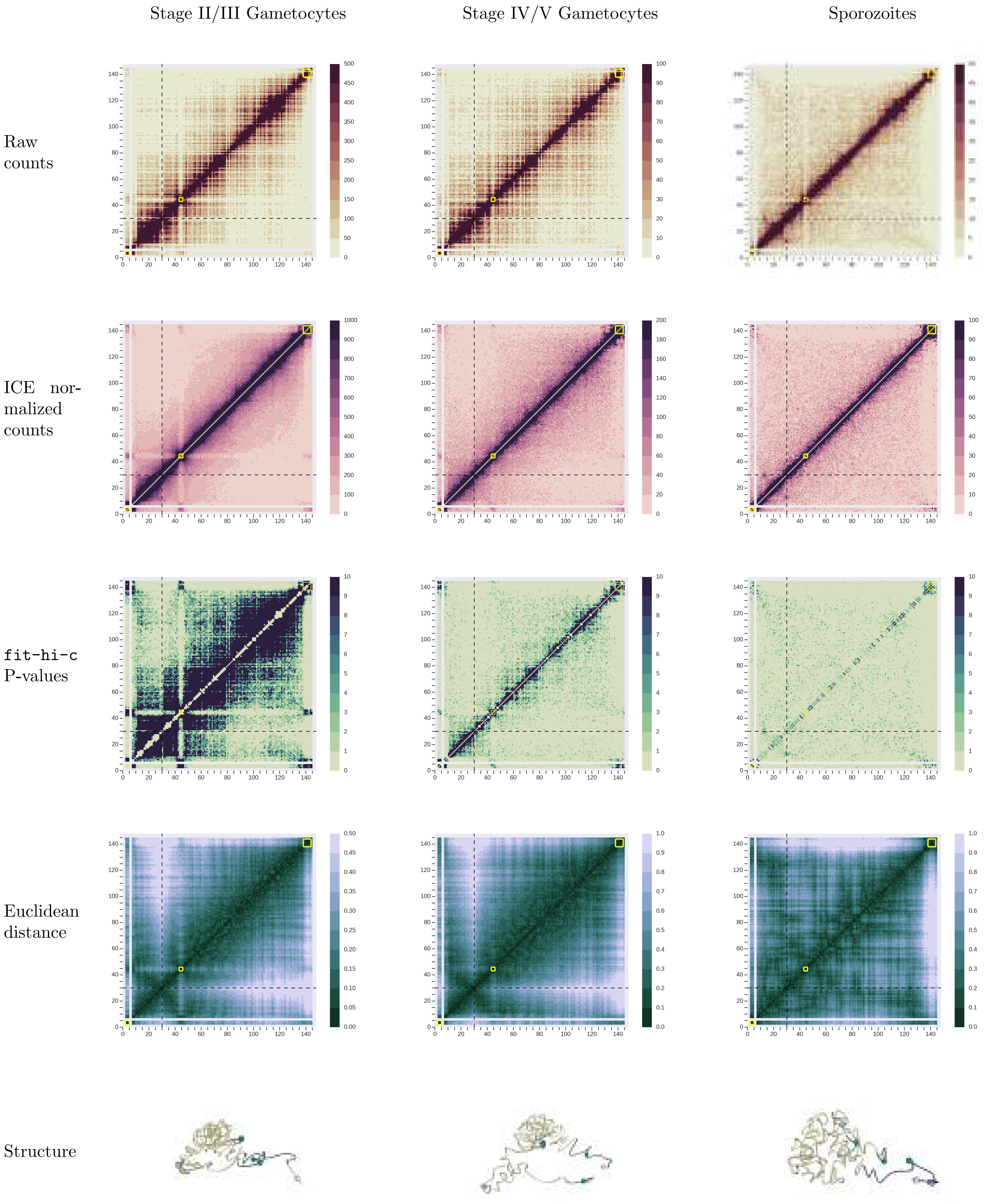

Chromosome 8

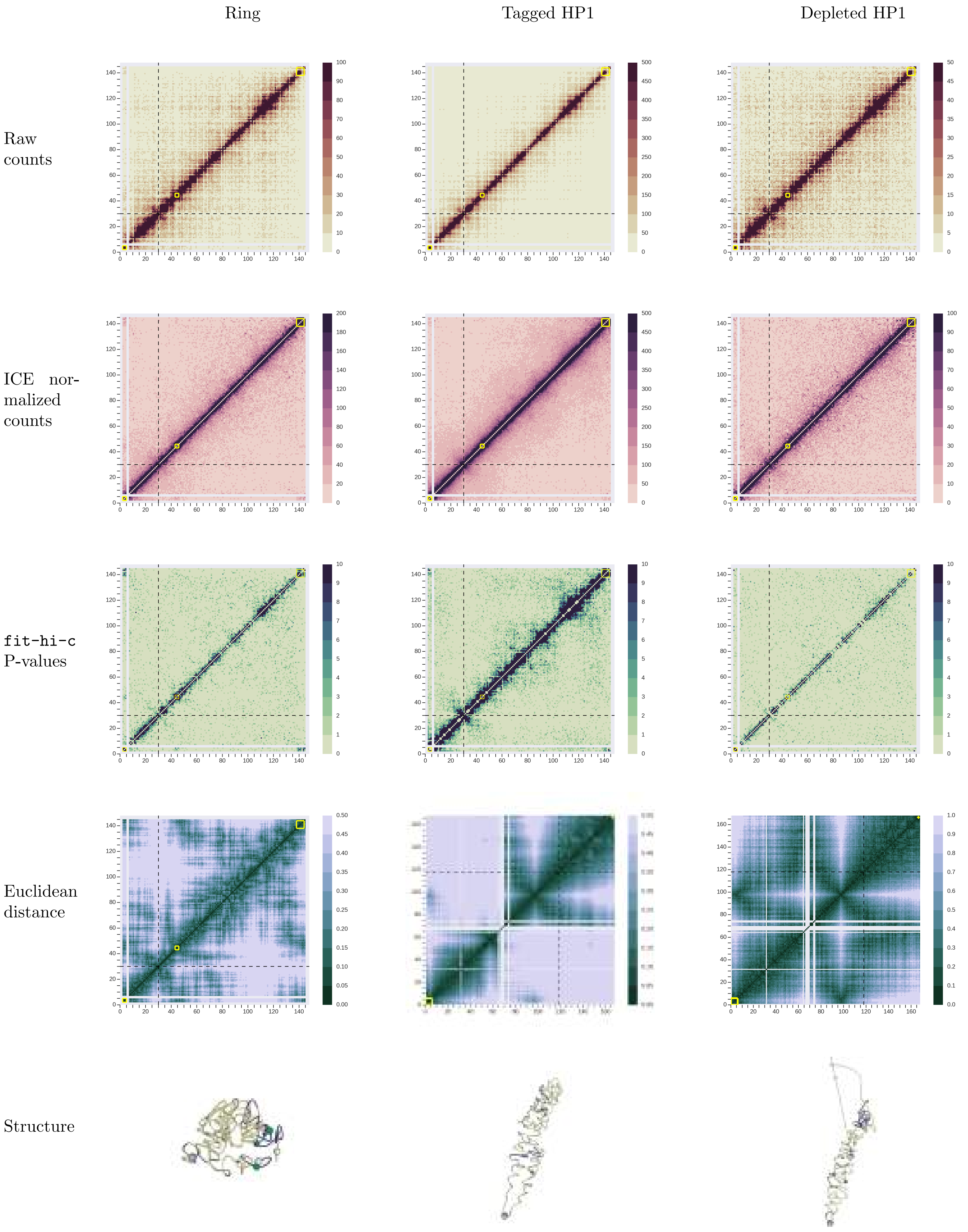

Chromosome 9

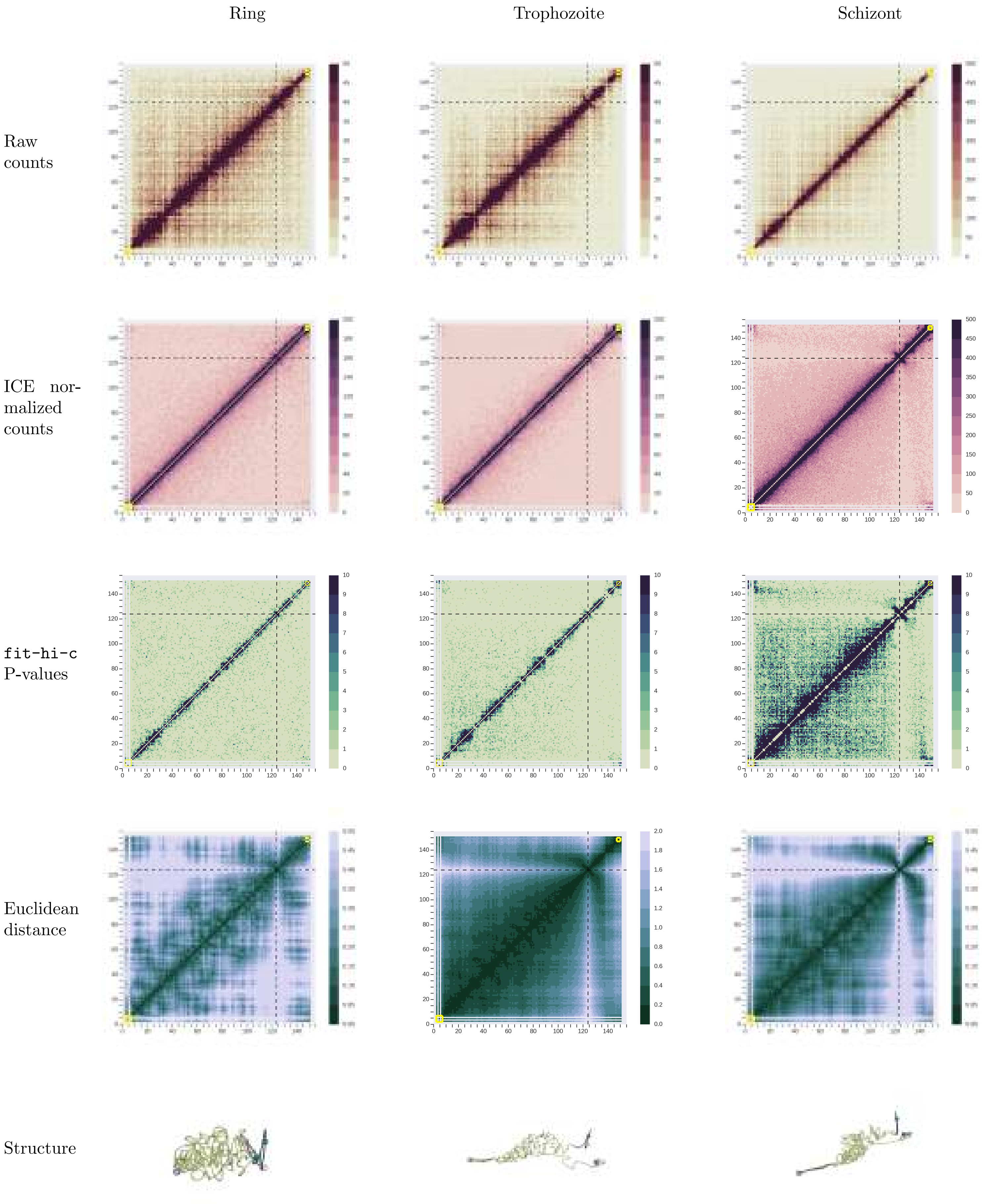

Chromosome 9

Stage II/III Gametocytes — Stage IV/V Gametocytes — Sporozoites

Raw counts

ICE normalized counts

`fit-hi-c` P-values

Euclidean distance

Structure

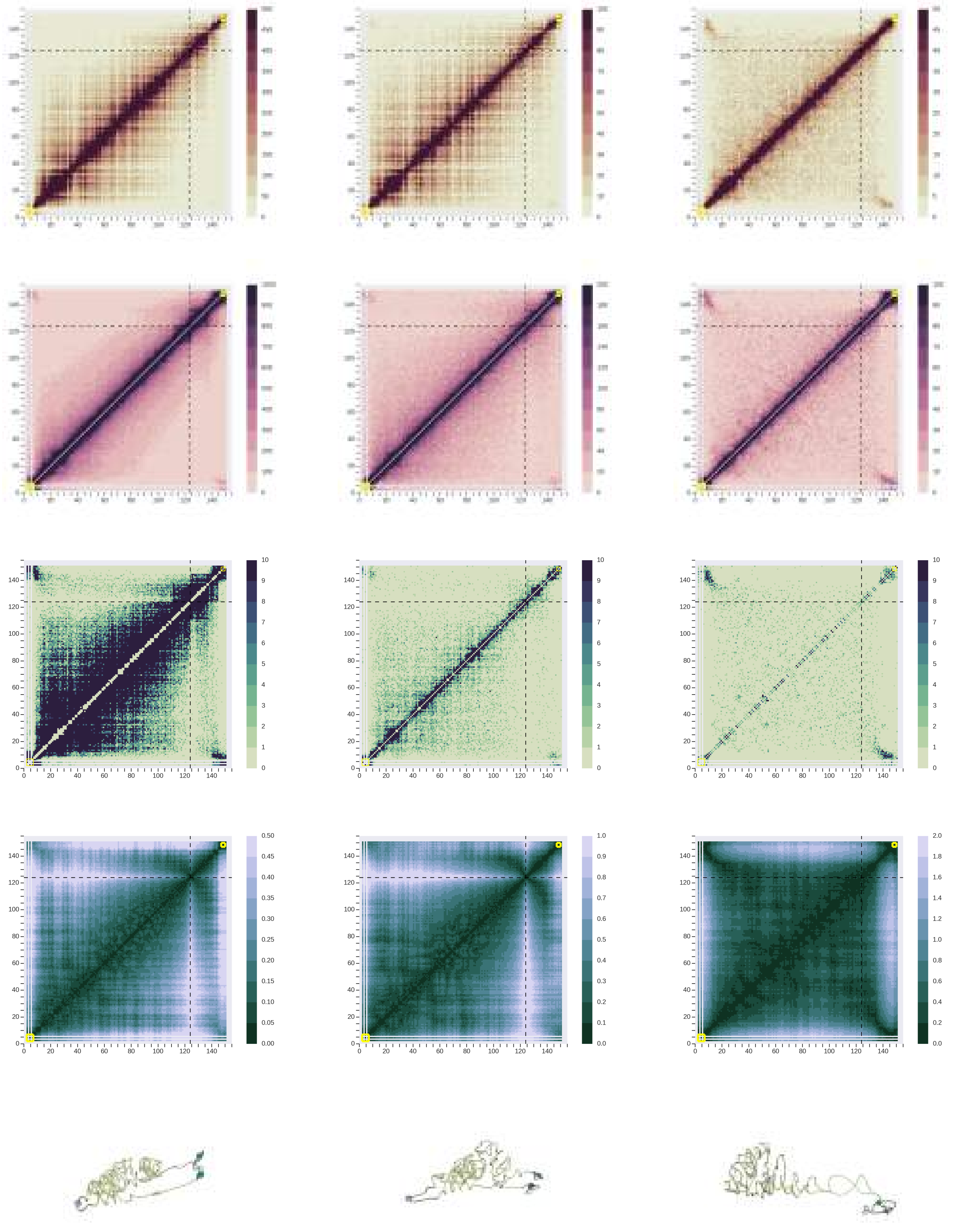

Chromosome 9

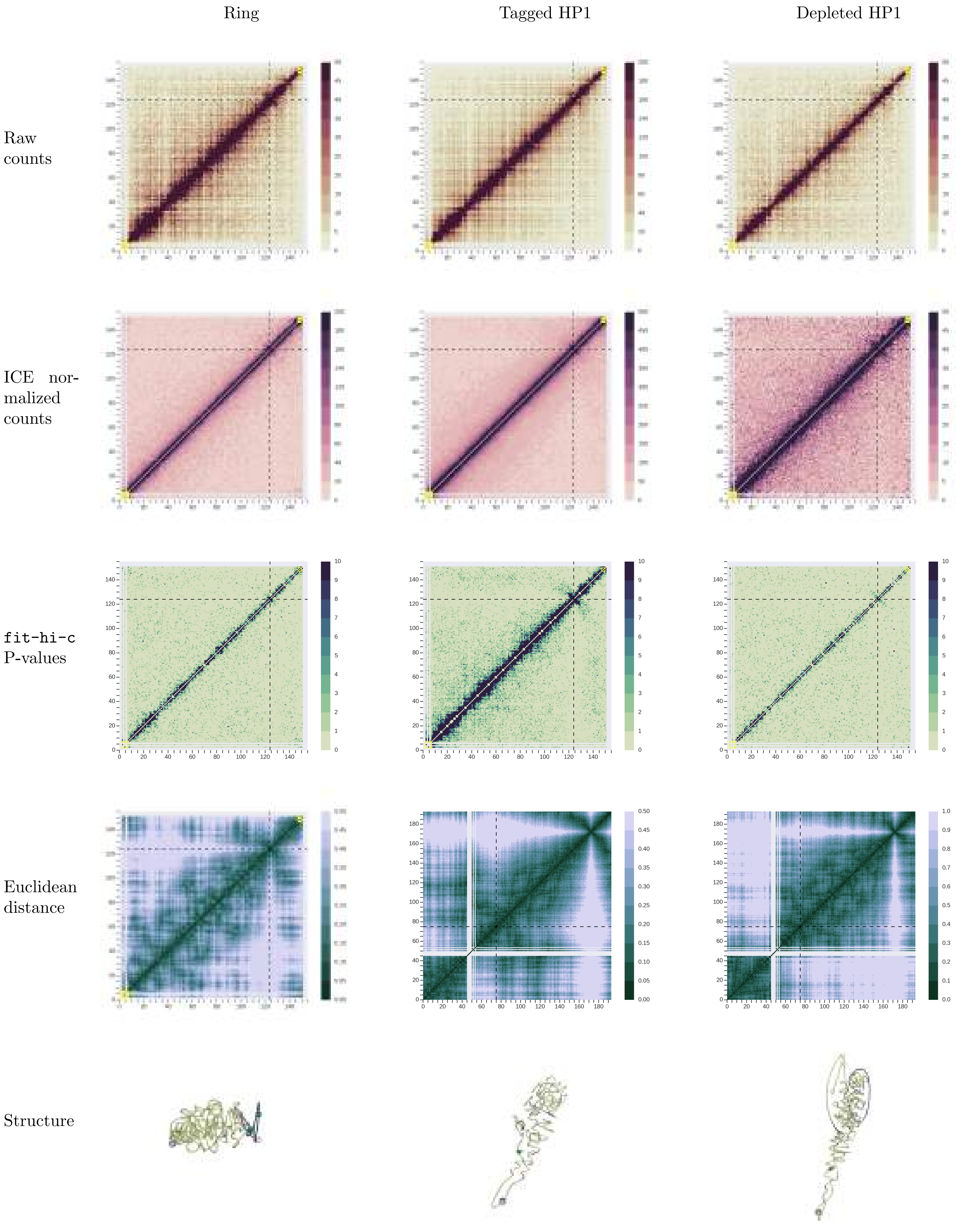

Chromosome 10

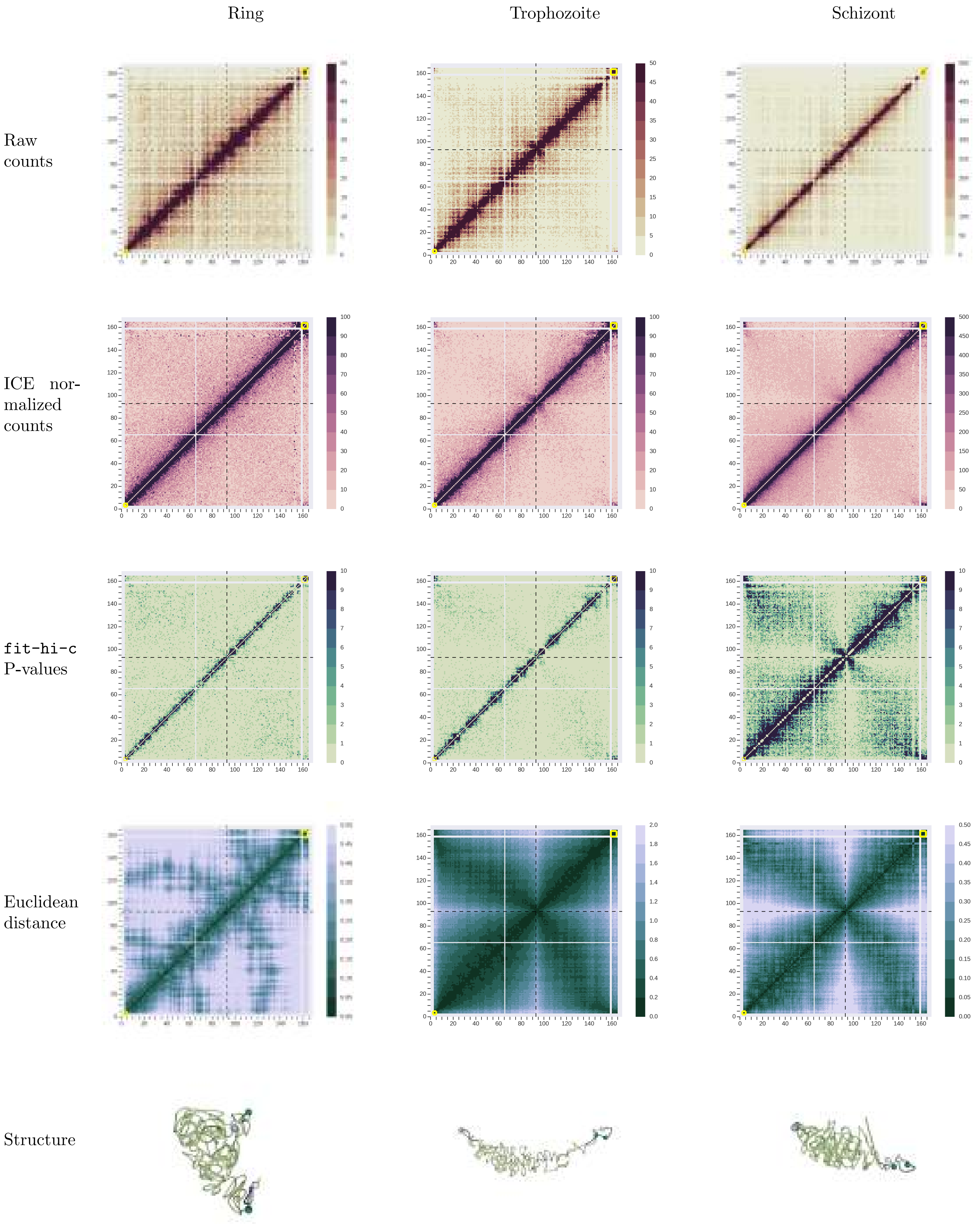

Chromosome 10

Stage II/III Gametocytes | Stage IV/V Gametocytes | Sporozoites

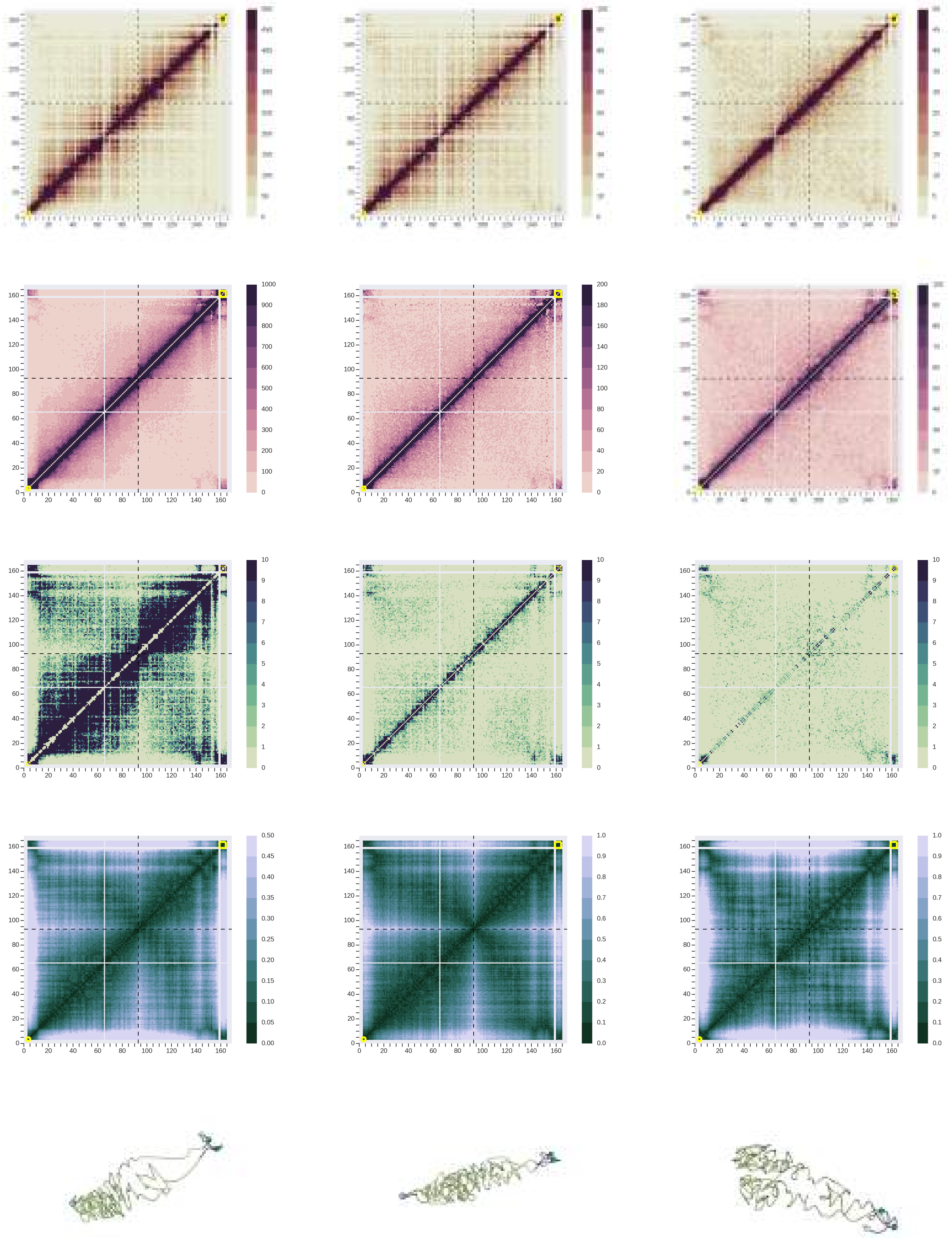

Chromosome 10

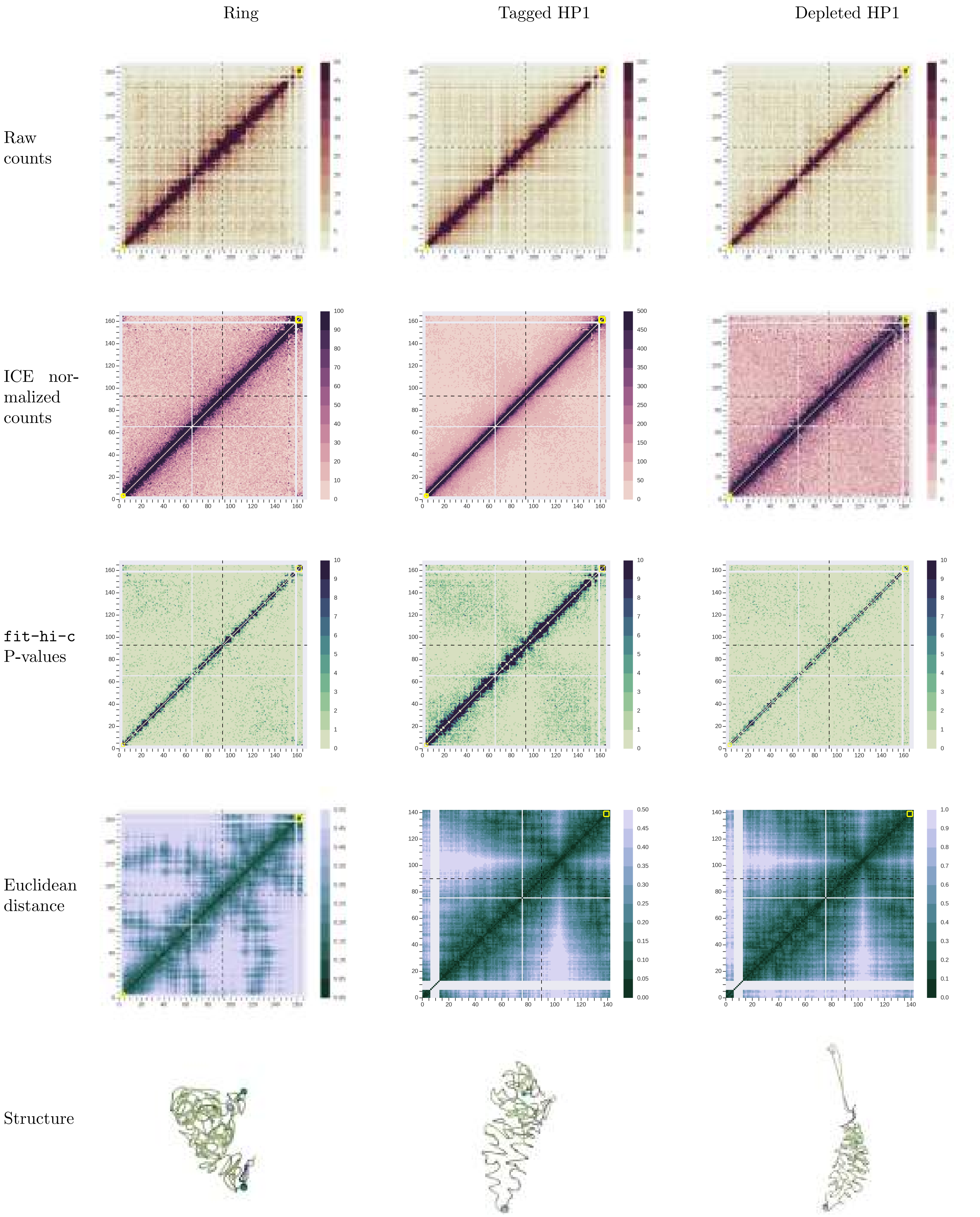

Chromosome 11

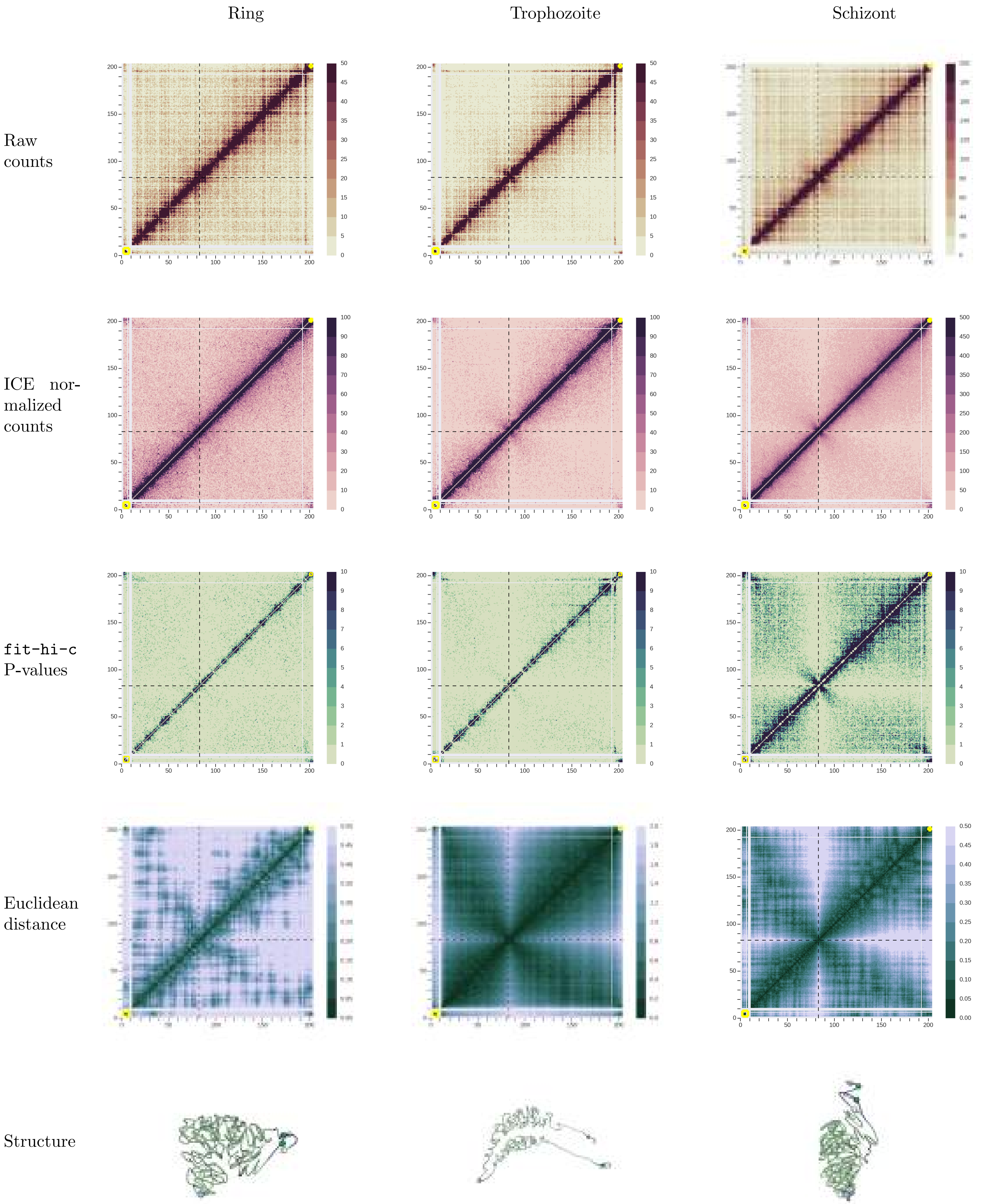

Chromosome 11

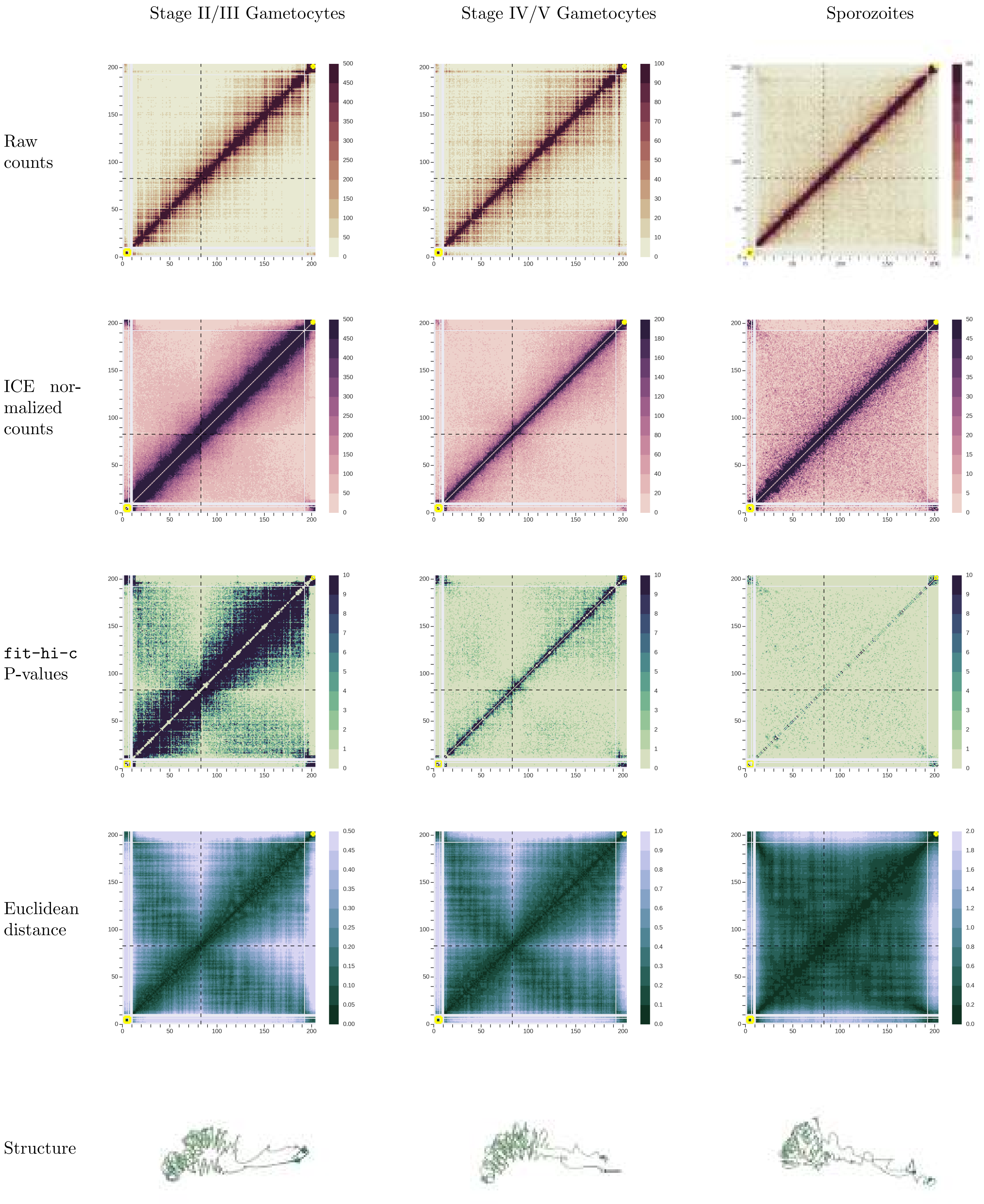

Chromosome 11

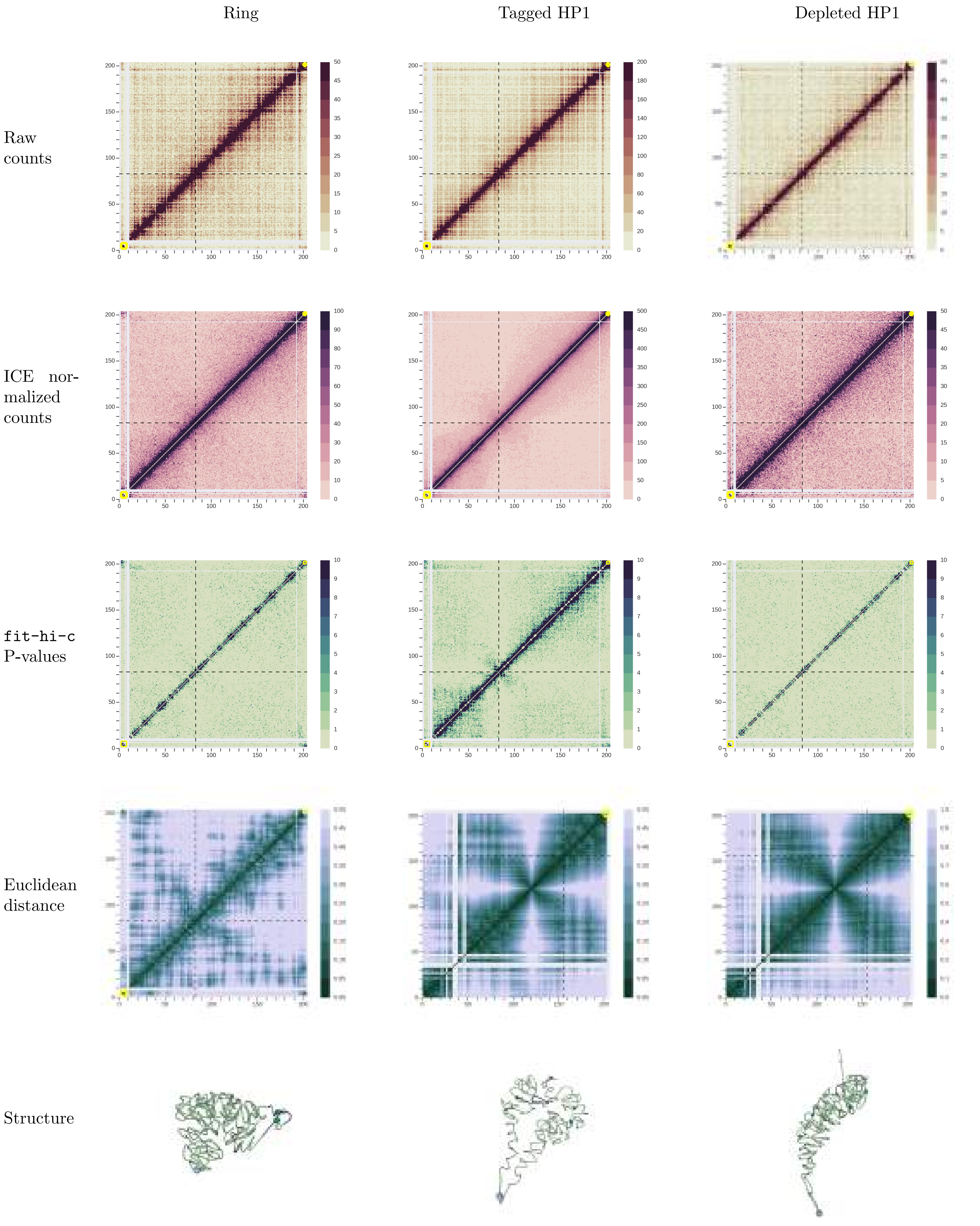

Chromosome 12

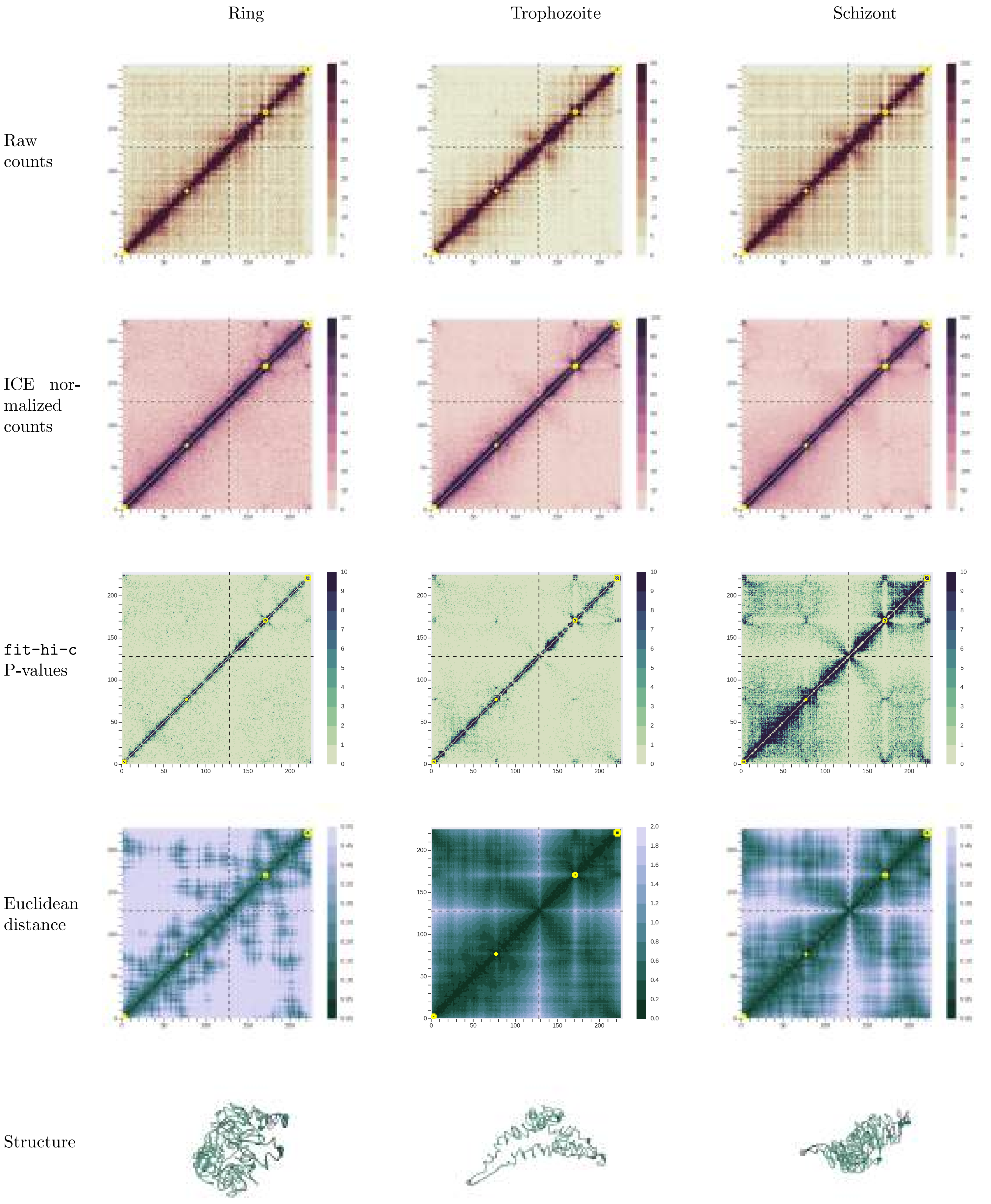

Chromosome 12

Stage II/III Gametocytes | Stage IV/V Gametocytes | Sporozoites

Raw counts

ICE normalized counts

`fit-hi-c` P-values

Euclidean distance

Structure

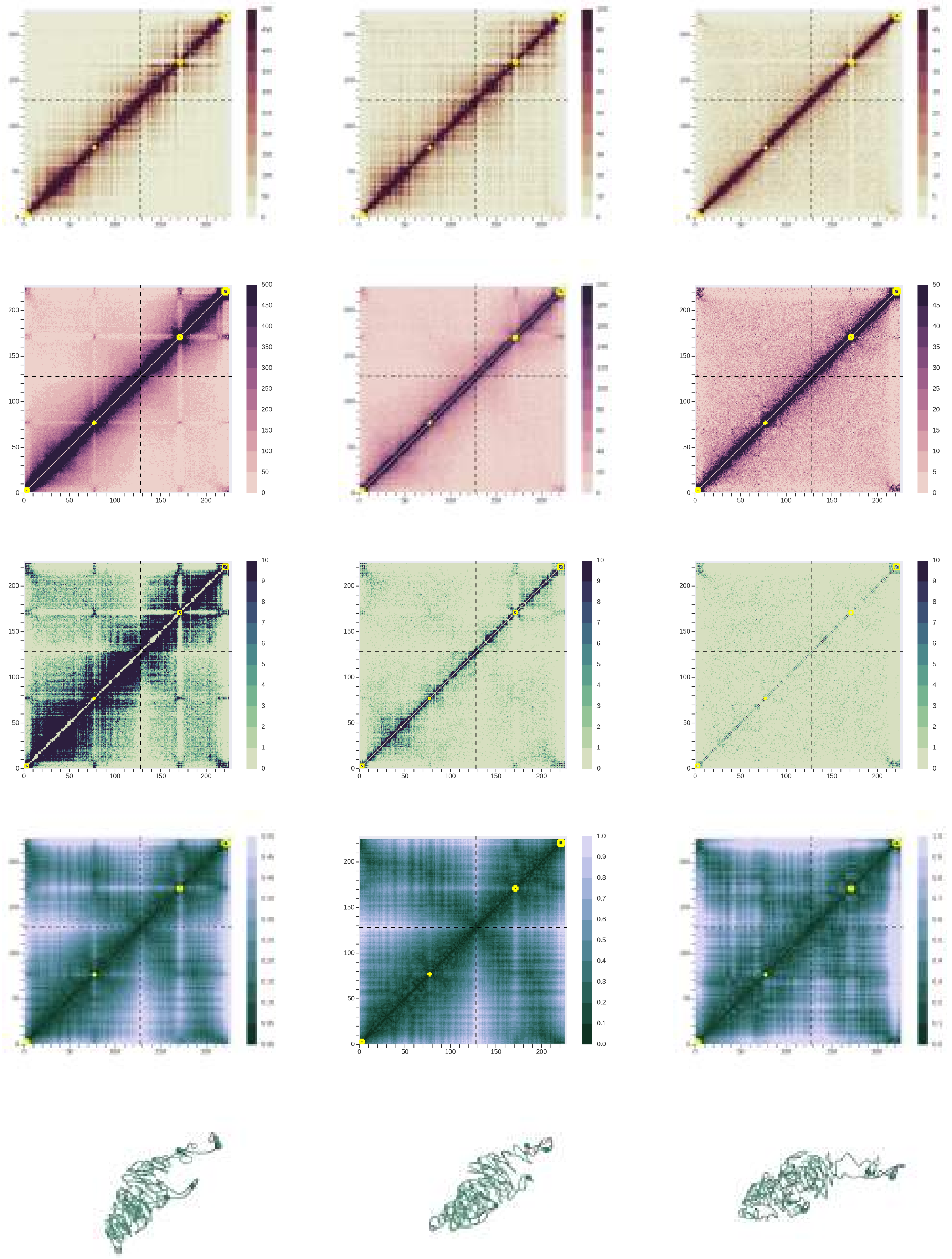

Chromosome 12

Ring | Tagged HP1 | Depleted HP1

Raw counts

ICE normalized counts

`fit-hi-c` P-values

Euclidean distance

Structure

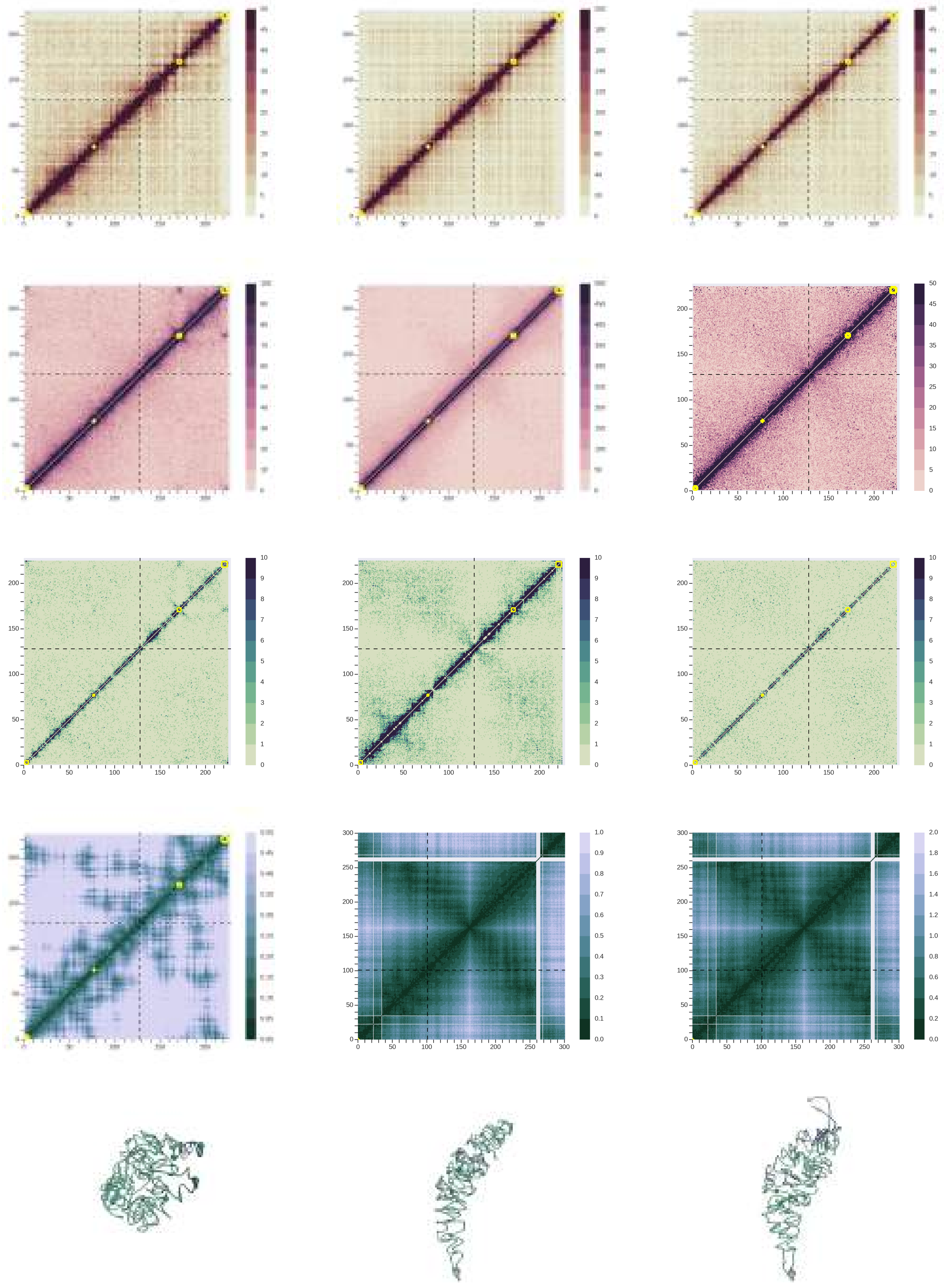

Chromosome 13

Ring

Trophozoite

Schizont

Raw counts

ICE normalized counts

`fit-hi-c` P-values

Euclidean distance

Structure

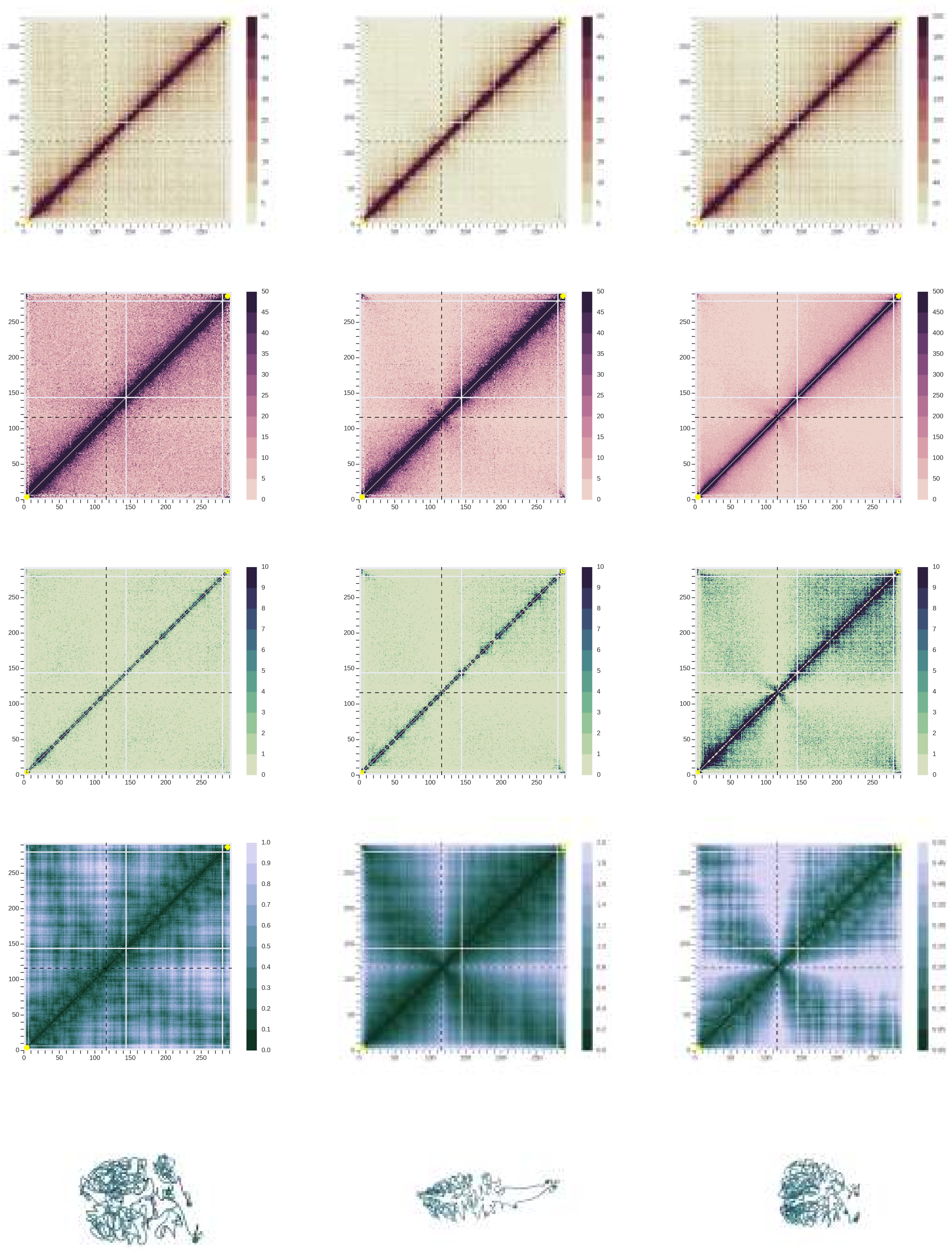

Chromosome 13

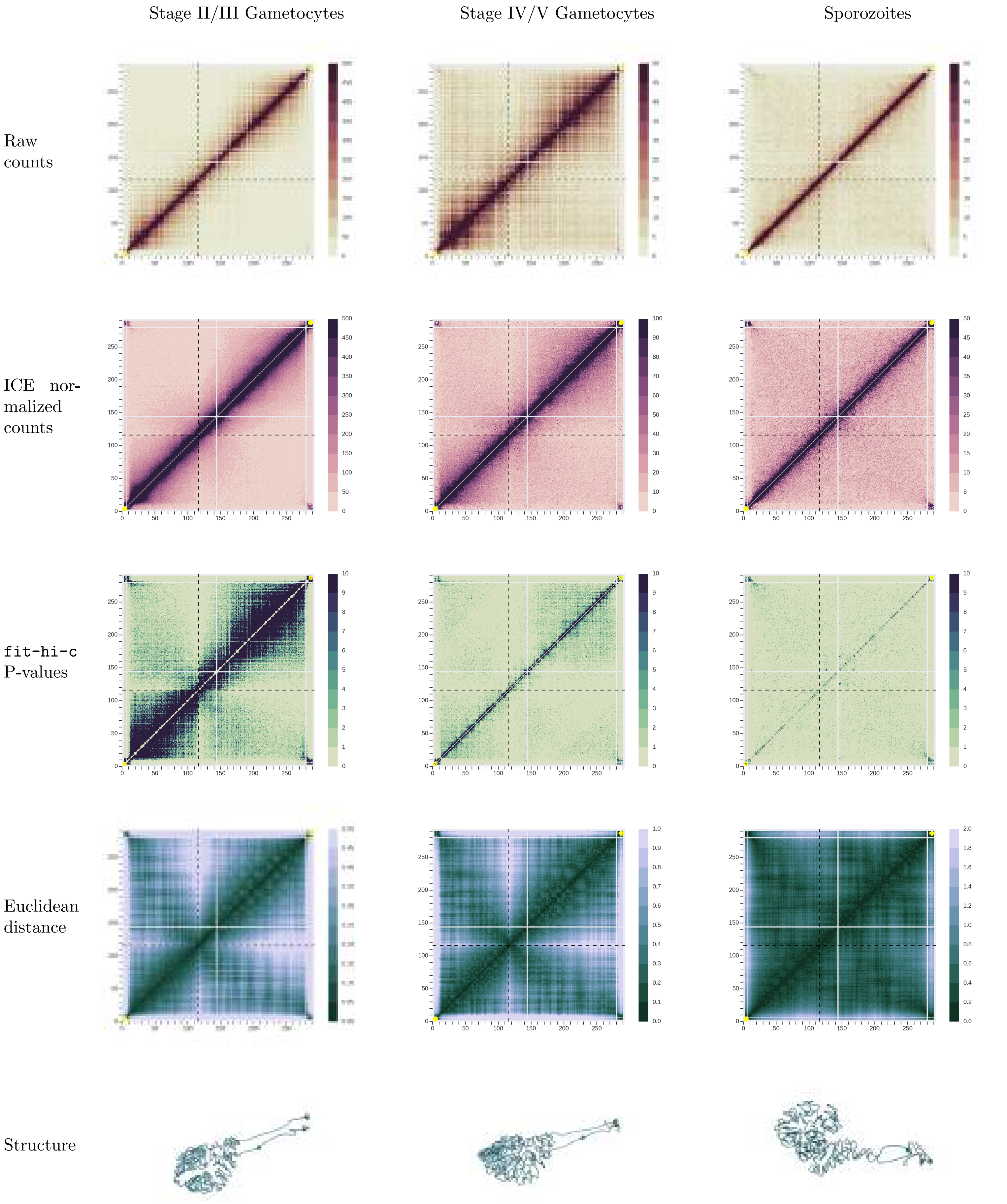

Chromosome 13

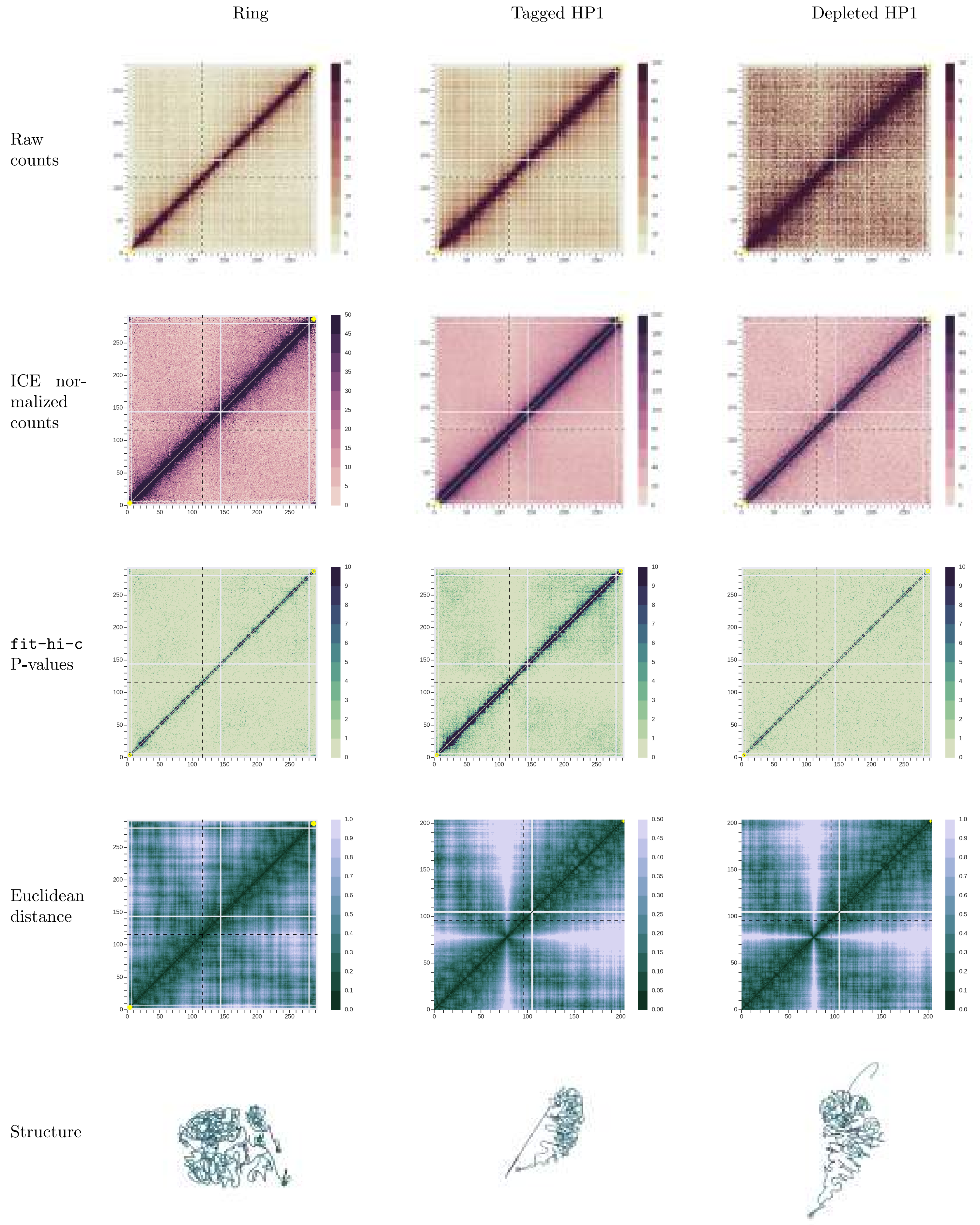

Chromosome 14

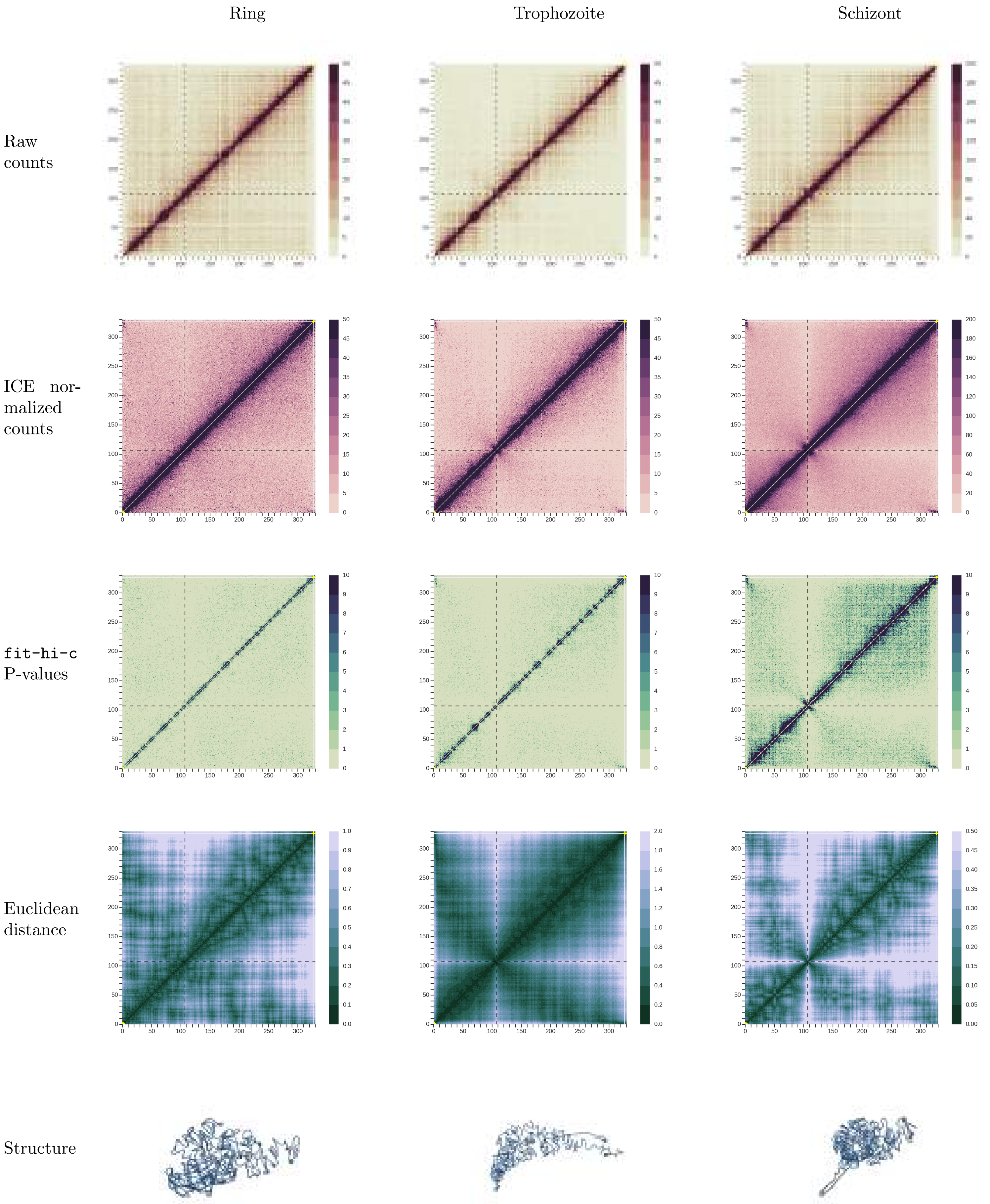

Chromosome 14

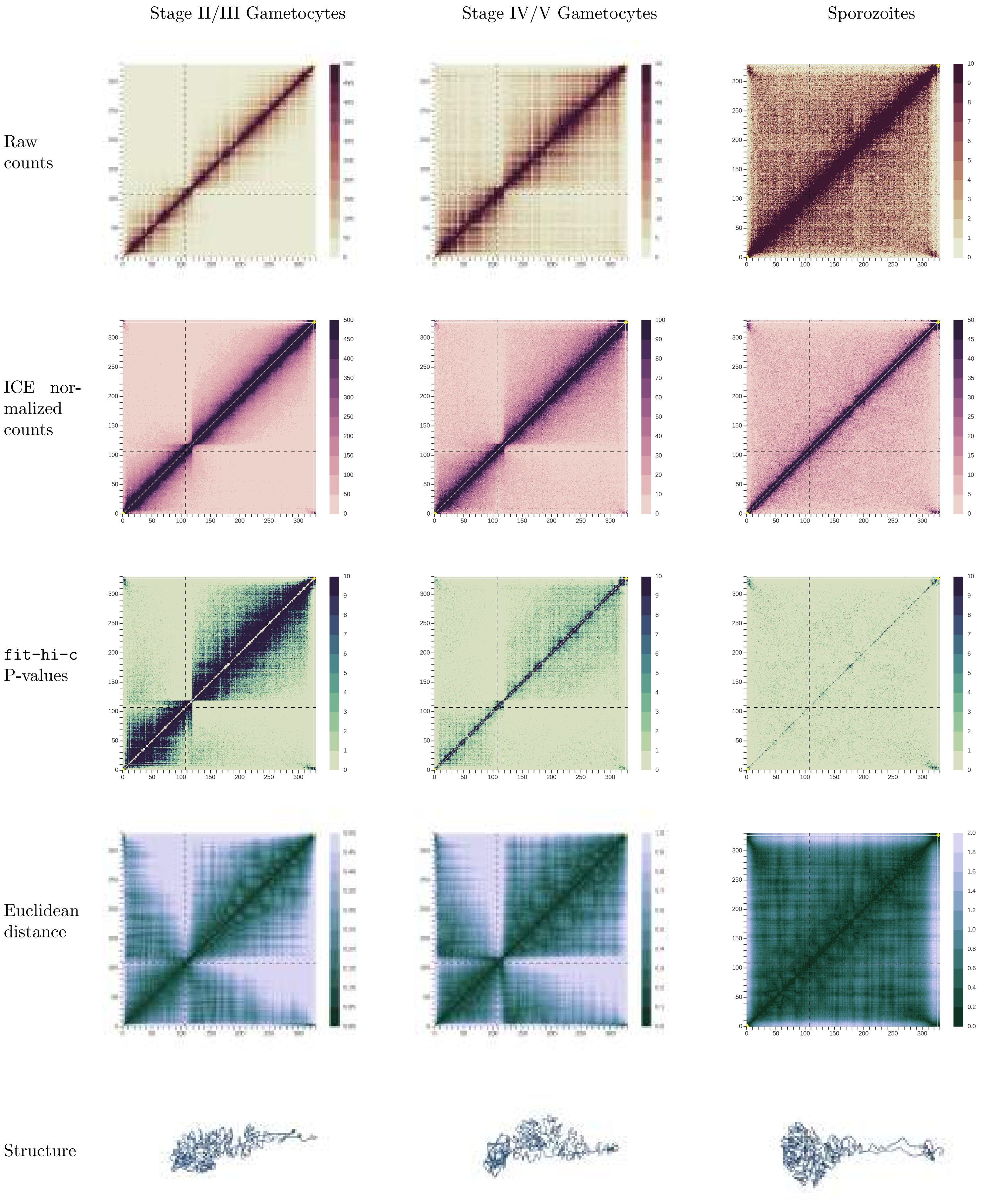

Chromosome 14

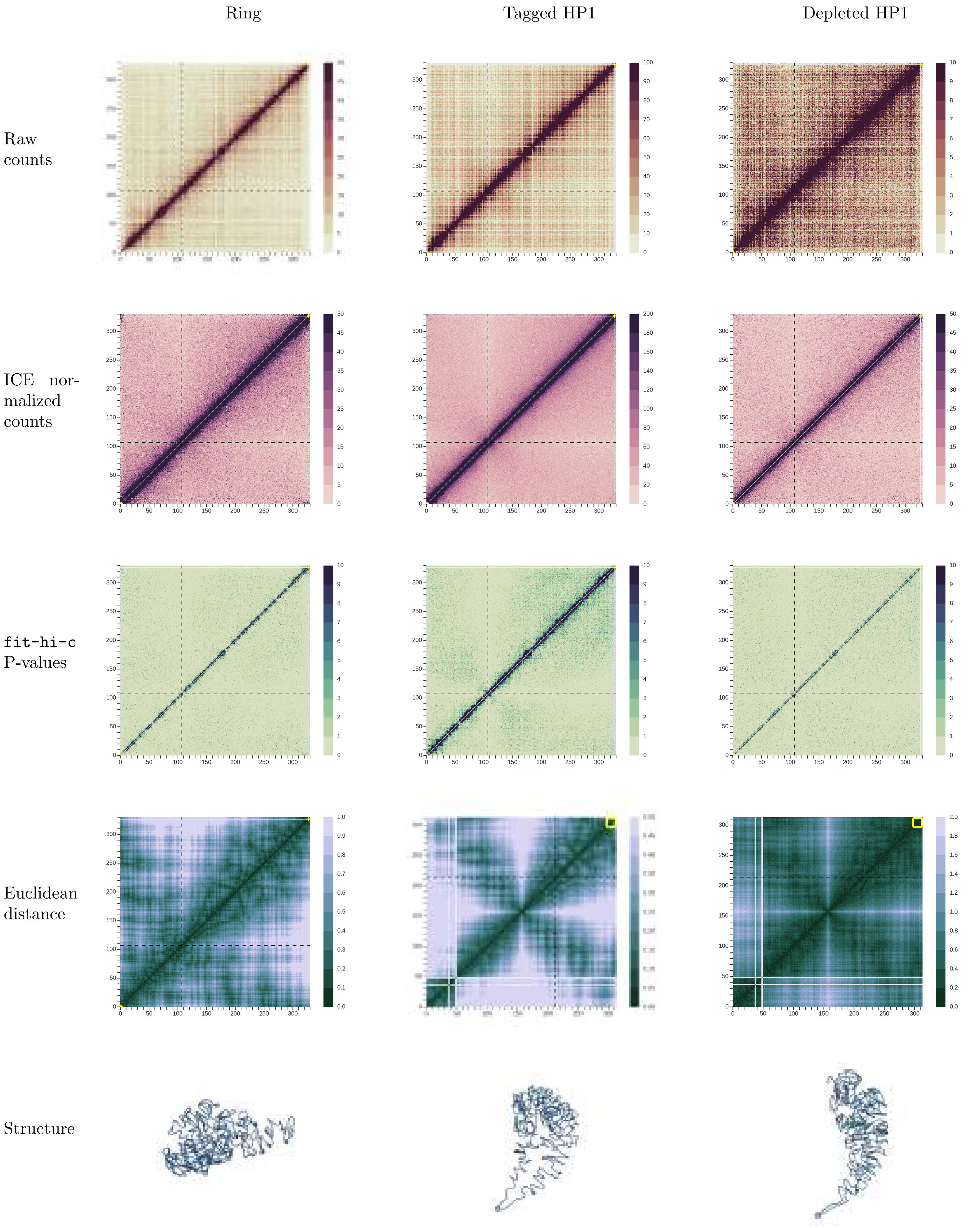

Supplement: Supplementary file 3 — Supplementary Data 1 [file 41467_2018_4295_MOESM3_ESM.pdf]
