# Supplementary material for: Changes in genome organization of parasite-specific gene families during the Plasmodium transmission stages
Source: Nat Commun. 2018 May 15;9:1910. doi: 10.1038/s41467-018-04295-5 (PMC5954139; doi:10.1038/s41467-018-04295-5)

Supplementary Data 2: Chromosome visualizations for *P. vivax* sporozoites. Each pages contains three *P. vivax* chromosomes. Within each page, the top row shows the raw contact count matrices at 10 kb resolution, the second row shows the ICE-normalized contact count matrices at 10 kb resolution, and the third row shows matrices of the fit-hi-c P-values assigned to the contacts. In these three matrices, the location of the centromere is indicated by a dashed black line, *vir* gene clusters are highlighted with a yellow box, and unmappable regions are colored in grey. The fourth row shows the pairwise Euclidean distance matrices derived from the 3D model, and the bottom row shows the inferred configuration in 3D with light blue spheres indicating centromeres, white spheres indicating telomeres and purple regions indicating the location of virulence gene clusters.

P. vivax **Sporozoites**

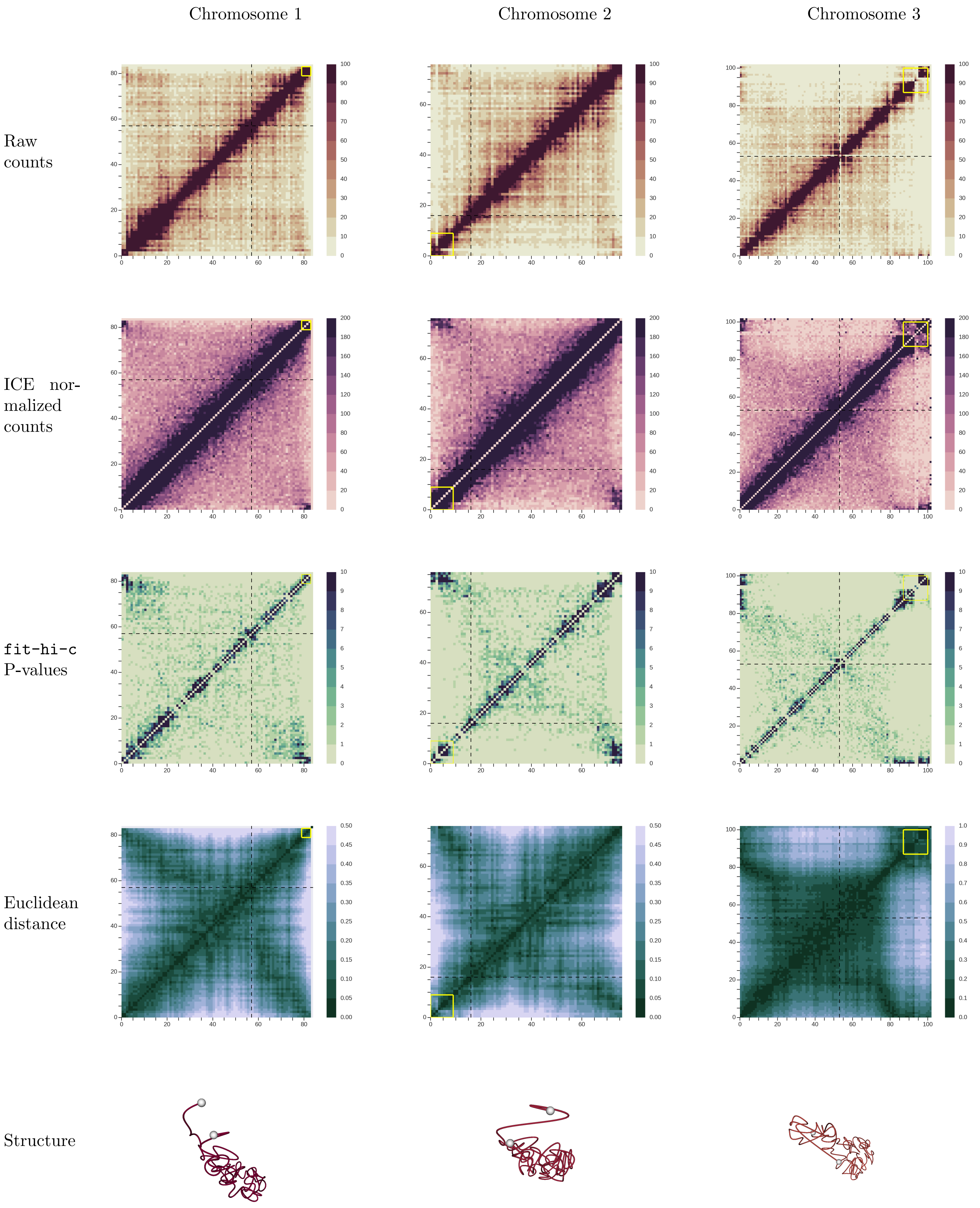

P. vivax **Sporozoites**

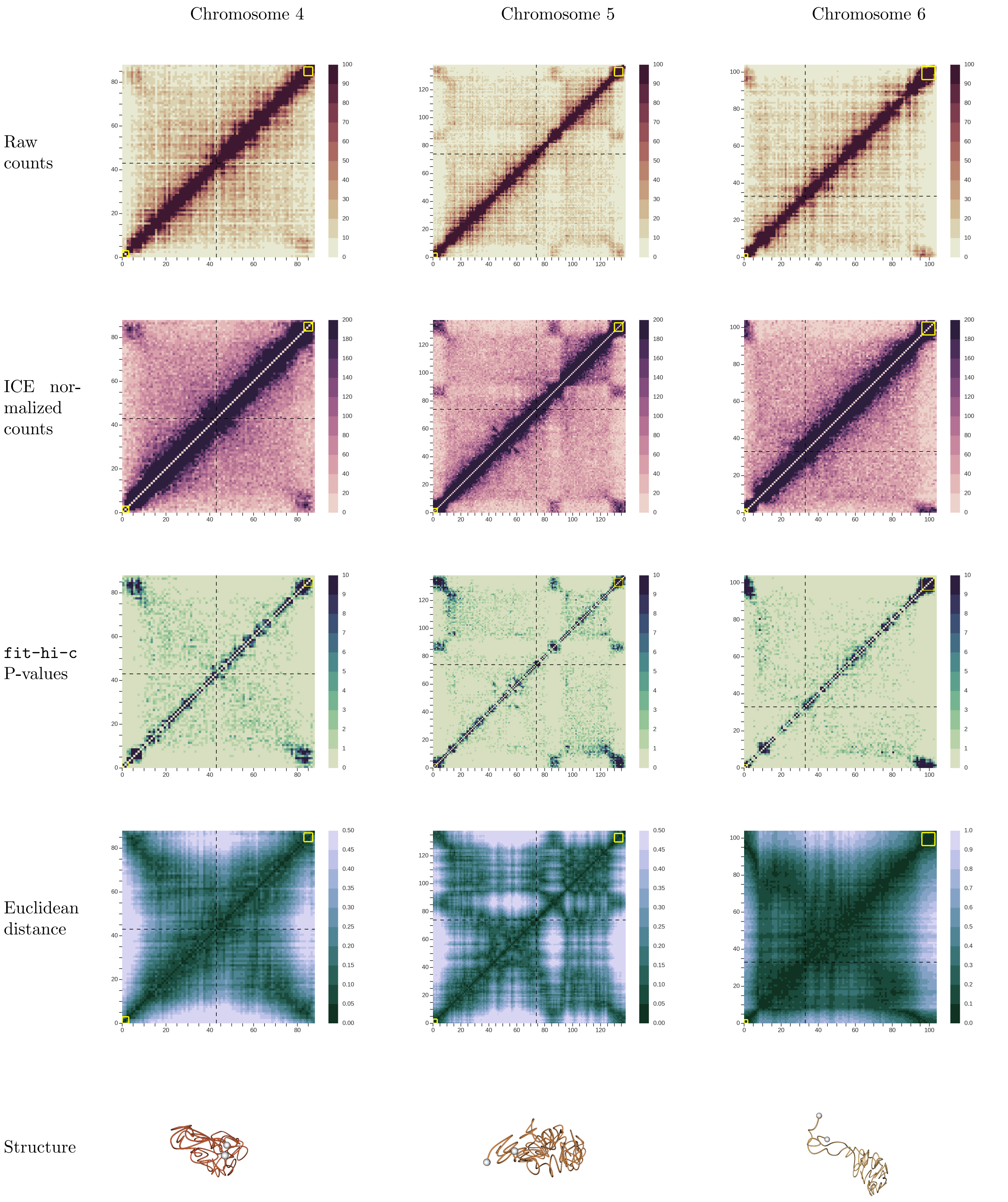

P. vivax Sporozoites

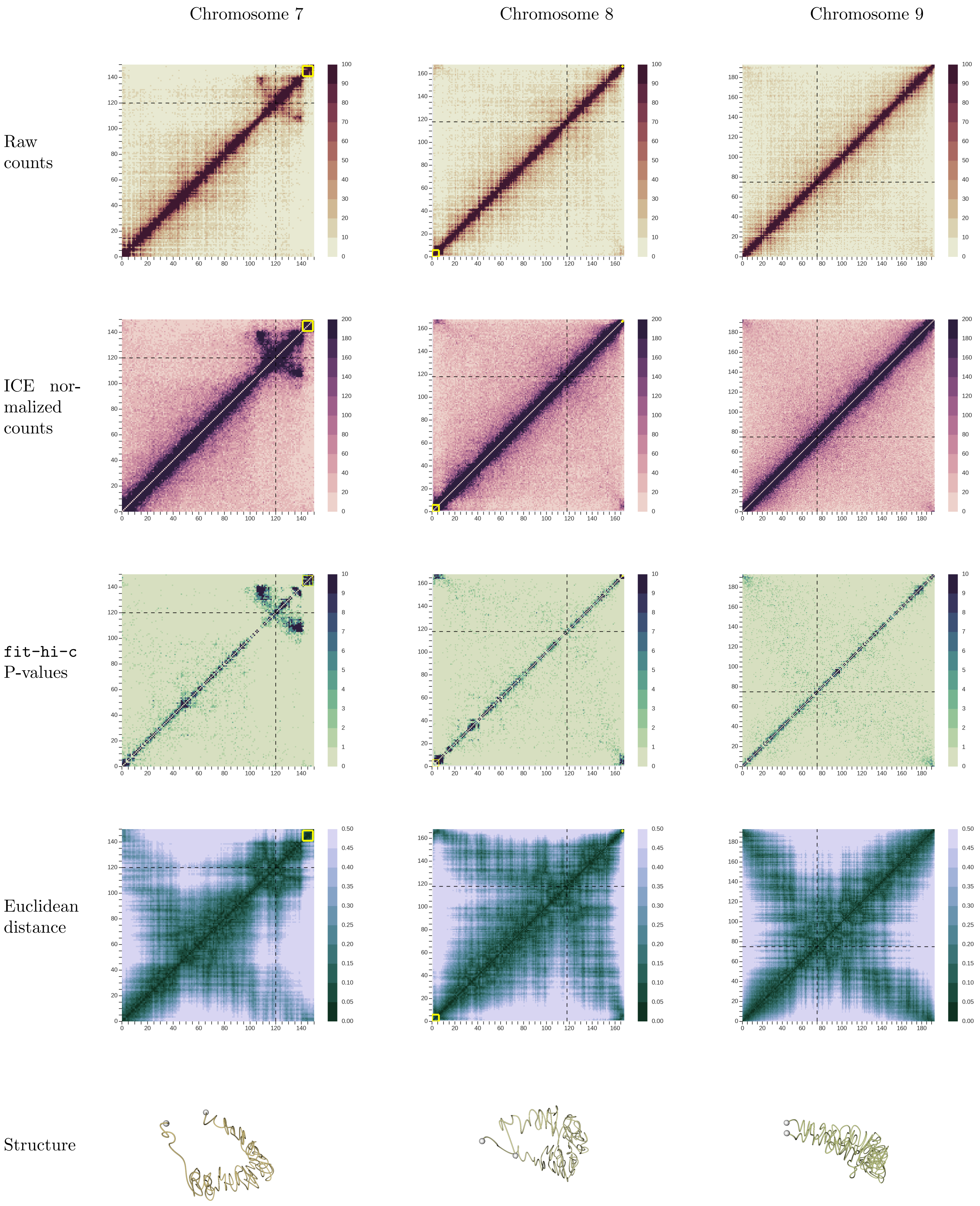

***P. vivax* Sporozoites**

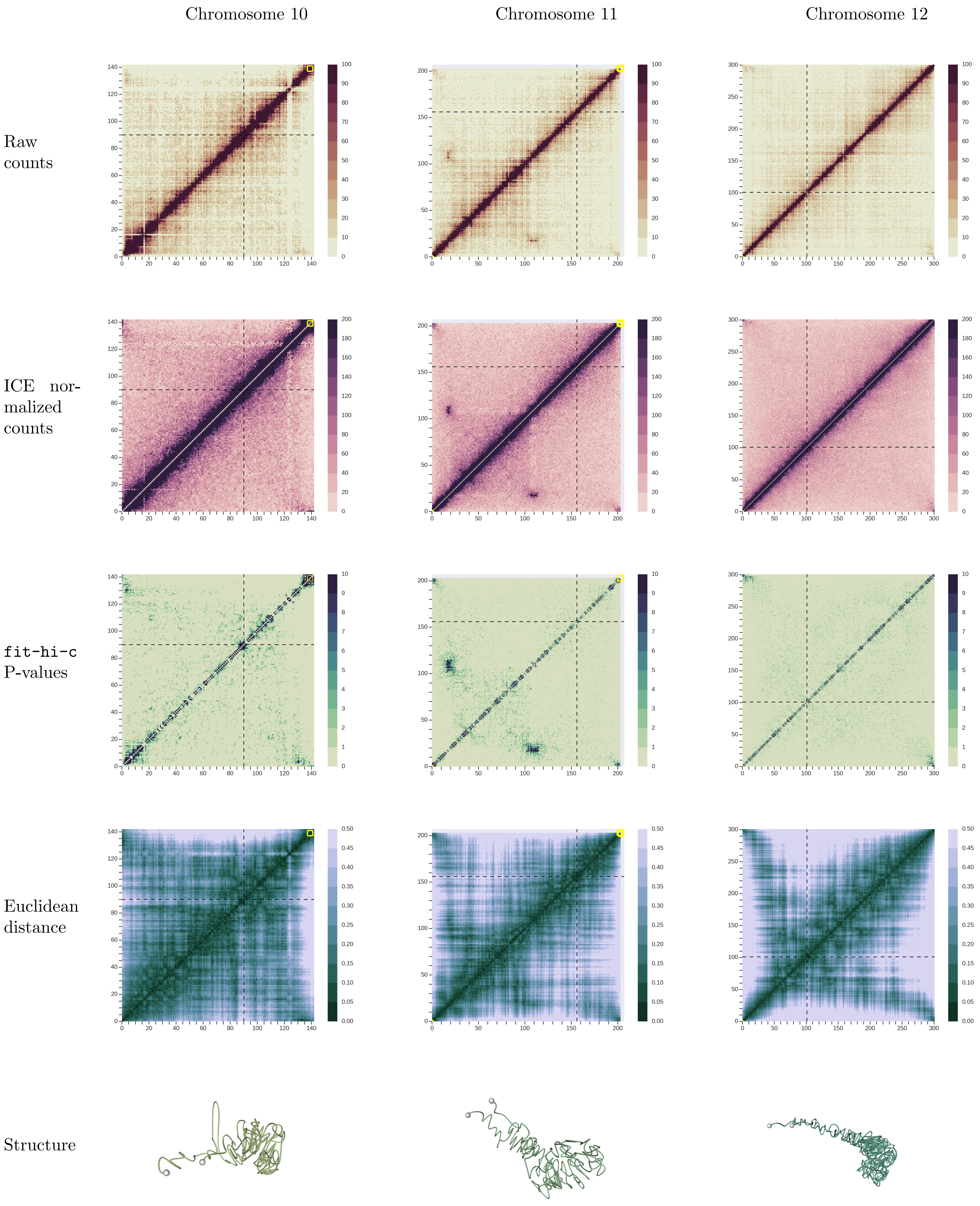

P. vivax Sporozoites

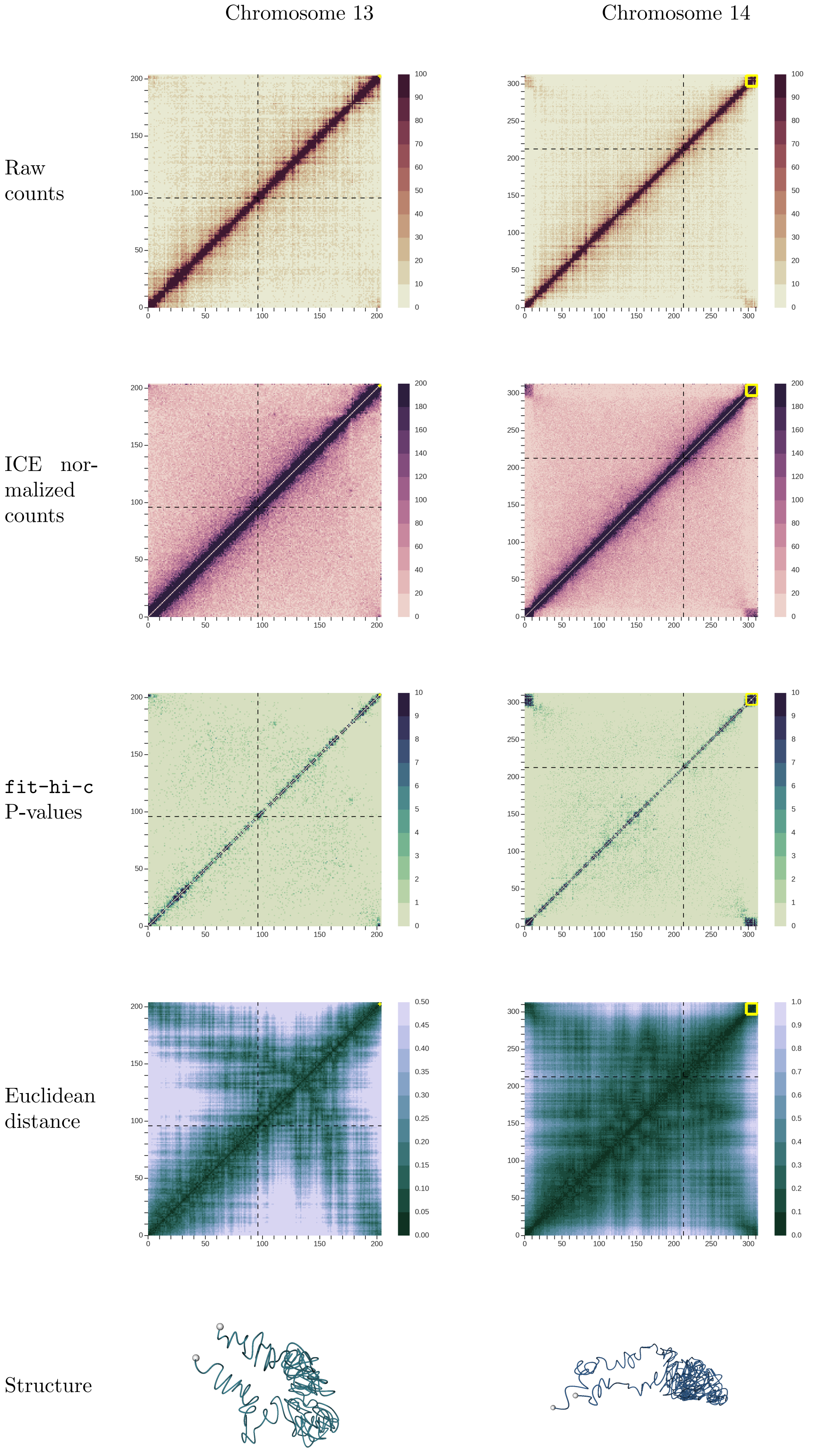

Supplement: Supplementary file 4 — Supplementary Data 2 [file 41467_2018_4295_MOESM4_ESM.pdf]
